# Guidelines on diagnosis and management of gastroesophageal reflux disease in infants, children and adolescents: a joint consensus from Italian pediatric societies (SIP and SIGENP) -part I. diagnosis

**DOI:** 10.1186/s13052-026-02218-5

**Published:** 2026-04-11

**Authors:** Silvia Salvatore, Caterina Strisciuglio, Elena Bozzola, Stefania Cappa, Antonio Corsello, Giovanni Di Nardo, Maurizio Fuoti, Antonino Gulino, Chiara Mameli, Massimiliano Orso, Licia Pensabene, Barbara Polistena, Renato Tambucci, Francesca Vassallo, Claudio Romano, Annamaria Staiano

**Affiliations:** 1 Pediatric Unit, Hospital “F. Del Ponte”, Department of Medicine and Technical Innovation, University of Insubria, Varese, Italy; 2 Department of Woman, Child and General and Specialized Surgery, University of Campania “Luigi Vanvitelli”, Via Luigi De Crecchio, 2 - 80138 Naples, Italy; 3 Pediatric Unit, Bambino Gesù Children’s Hospital, IRCCS, 00165 Rome, Italy; 4 Associazione Italiana Neonati Reflussanti (AINER), Rome, Italy; 5 Department of Pediatrics, Istituto Mediterraneo per i Trapianti e Terapie ad Alta Specializzazione - IRCCS ISMETT, UPMC, Palermo, Italy; 6 Department of Clinical-Surgical, Diagnostic and Pediatric Sciences, University of Pavia, Pavia, Italy; 7 Department of Neurosciences, Mental Health and Sensory Organs (NESMOS), Sapienza University of Rome, Pediatric Unit, Pediatric Gastroenterology and Endoscopy Division, Sant’Andrea University Hospital, Rome, Italy; 8 Pediatric Gastroenterology and Endoscopy Unit, Children’s Hospital, ASST Spedali Civili, Brescia, Italy; 9 Family Pediatrician, ASP, Catania, Italy; 10 Department of Pediatrics, V Buzzi Children’s Hospital, Università di Milano, Milan, Italy; 11 Department of Biomedical and Clinical Science, Università di Milano, Milan, Italy; 12 C.R.E.A. Sanità (Centre for Applied Economic Research in Healthcare), Rome, Italy; 13 Pediatric Unit, Department of Medical and Surgical Sciences, University “Magna Graecia” of Catanzaro, 88100 Catanzaro, Italy; 14 University of Rome Tor Vergata, Rome, Italy; 15 Gastroenterology and Nutrition Unit, Bambino Gesù Children’s Hospital IRCCS, Rome, Italy; 16 Department of Integrated Activities for Mothers and Children, General and Specialty Pediatrics Unit, University of Naples “ Federico II”, Naples, Italy; 17 Pediatric Gastroenterology and Cystic Fibrosis Unit, Department of Human Pathology in Adulthood and Childhood “G. Barresi”, University of Messina, 98125 Messina, Italy; 18 Department of Translational Medical Science, Section of Paediatrics, University of Naples “Federico II”, Unit of Pediatrics, A.O.U. “Federico II”, Naples, Italy

**Keywords:** Gastroesophageal reflux, Children, GERD, Proton pump inhibitors, Endoscopy, pH-monitoring, Esophageal impedance, Esophagitis

## Abstract

**Background:**

Gastroesophageal reflux disease (GERD) is a common reason for referral to pediatric and gastroenterology clinics. Still, uncertainty and variability exist in the evaluation, diagnostic approaches and management among different age groups and clinicians. This study aimed to report the results of a Guidelines process and an Italian expert Consensus focusing on the diagnosis of GERD in children to improve the clinical approach to these patients.

**Methods:**

A multidisciplinary board of 16 participants identified eight PICO (Population/Patient/Problem, Intervention, Comparator and Outcome) questions, including definition, signs and symptoms, risk factors, diagnostic tests, treatment and prognosis of gastroesophageal reflux (GER) and GERD in children: four PICO questions were related to the diagnosis of GERD. Four databases (PubMed/Medline, Embase, Web of Science and Google Scholar) were searched from their inception to May 11, 2024, limited to children (0–18 years) and English language. For each PICO question a specific search string was developed. Guidelines, systematic reviews and clinical studies on diagnosis and management in children were considered to formulate evidence-based recommendations. Agreement was defined based on a 9-point Likert scale. A two-round Delphi method was conducted and consensus was defined as ≥ 80% agreement or disagreement. This paper focused on clinical symptoms and diagnosis of GER and GERD in pediatric ages.

**Results:**

123 studies were included that satisfied systematic research criteria. The panel provided 14 recommendations on diagnosis of GER and GERD and practice points for specific questions to clarify some clinical issues.

**Conclusion:**

Symptoms of GER(D) are common in the pediatric population while regurgitation is frequently reported in the first months of life. In infancy symptoms mostly disappear spontaneously or after dietary modification. In infants and children with esophageal or extresophageal persistent symptoms, esophageal pH-MII monitoring and endoscopy are indicated to detect and characterize reflux episodes, phenotypes of GERD and esophagitis. In older children and adolescents reporting typical heartburn or in those with severe neurological impairment, a time-limited empirical trial of PPI can be considered as an initial diagnostic approach, with further testing reserved for non-responders.

**Supplementary Information:**

The online version contains supplementary material available at 10.1186/s13052-026-02218-5.

## Introduction

Gastroesophageal reflux disease (GERD) is defined by the presence of troublesome symptoms or esophageal or extra-esophageal complications due to the reflux of gastric content into the esophagus (GER) [1–3]. GER occurs typically heseveral times a day, especially after meals but when the amount of content of the reflux is abnormal, natural defences are weaker or individual sensitivity is higher, it causes GERD. GER is often silent but may induce a broad spectrum of symptoms including regurgitation, vomiting, irritability, heartburn, epigastric or chest pain, dysphagia, respiratory and sleeping problems, may reduce quality of life and causes esophageal complications in predisposed individuals [1].

The difference between GER, GERD and other conditions is often challenging in pediatric ages because of the lack of a specific symptom of GERD and a gold standard diagnostic test [1]. Moreover, the inability of infants and young children to self-report symptoms, coupled with the reliance on parental interpretation, further complicates the assessment. Over the past two decades, diagnostic techniques for GERD in pediatric populations have expanded and improved, enhancing the identification of infants and children who require treatment and thereby possibly reducing the inappropriate use of acid-suppressant medications [4–7]. Between 2013 and 2018 European and American GERD pediatric guidelines have been published [1, 8, 9] but the diffusion, implementation, clarity and practicability have been considered suboptimal [10–12]. In 2025, the Italian Societies of Gastroenterology, Endoscopy and General Medicine reached a consensus and produced recommendations for the diagnosis and management of GERD in adult patients [2]. Although GERD is recognized as one of the most common clinical conditions, considerable uncertainty remains regarding its optimal diagnostic evaluation and management [2].

In order to assist pediatricians, general practitioners, and gastroenterologists in managing pediatric patients, this work aims to provide a thorough update on the diagnosis of GER and GERD in infants and children. It will also offer current, evidence-based recommendations and practical guidance for the assessment of GERD in pediatric ages.

## Methods

### Participants and structure

A multidisciplinary panel of 16 participants including Italian pediatric gastroenterologists with expertise in the diagnosis and management of pediatric GERD, a family pediatrician, a representative of a patient association (the President of the Italian Association of Neonates with reflux, AINER), members of an external Agency (C.R.E.A. Sanità) with experience in data analysis and applied healthcare research and authors of Italian guidelines on different topics was convened by the Presidents of the Italian Society of Pediatrics (SIP) (A.S.) and of the Italian Society of Pediatric Gastroenterology Hepatology and Nutrition (SIGENP) (C.R.). The Evidence Review Team (ERT) from C.R.E.A. Sanità, composed of experts in evidence synthesis conducted a preliminary search of international guidelines on the diagnosis and management of GER and GERD in children to support the panel in the development of PICO questions. A systematic literature search was performed in PubMed, Embase, Web of Science, and Google Scholar on February 22, 2024 (Additional File 1). The quality of the identified guidelines was assessed using the AGREE II tool [13]. PICO questions from the included guidelines were extracted, analyzed, and presented to the panel, which then used a Delphi process to prioritize them and define a final list of eight key questions including four related to the diagnosis of GER and GERD considered critical to address (Table 1).

**Table 1:** List of clinical questions identified as relevant for this guideline

Questions
1. What is the definition of GER and GERD?
2. What are the signs and symptoms associated with GER and GERD?
3. What are the risk factors for GERD?
4. What is the value of different diagnostic testing for GERD?
5. What is the evidence of effectiveness of pharmacologic treatment for GER and GERD?
6. What is the effectiveness of different non-pharmacologic treatment options for GER and GERD?
7. What is the indication and the effectiveness of different surgical/endoscopic treatment options for GERD?
8. What is the prognosis of GER and GERD and what are prognostic factors?

The panel decided to formulate Questions 1 and 8 as narrative clinical questions, due to their descriptive nature.

Systematic literature searches were conducted in PubMed, Embase, and Web of Science on May 11, 2024. The complete search strategies are provided in Additional File 1. Manual retrieval of additional original studies and reviews from the reference lists of papers identified through the systematic search was also performed by all authors. When considered relevant to the purpose of this document, these studies were included in the corresponding PICO evidence summary.

### Systematic review of international guidelines on GER and GERD

Two reviewers carried out the study selection process independently in two phases. First, titles and abstracts were screened according to predefined inclusion criteria: English-language guidelines focusing on diagnosing GER and GERD in pediatric populations. Subsequently, potentially eligible full-text articles were assessed. Any disagreements between reviewers were resolved through discussion. Data extraction was performed by one reviewer and verified by a second. Three independent reviewers assessed the methodological quality of guidelines through the AGREE II tool. The literature selection process, the list of excluded studies and the reasons for exclusion are illustrated in Additional File 1 (PRISMA 2020 Flow Diagram).

Three guidelines [1, 8, 14] were included in the final analysis. The first document [1] dated 2018, was a clinical practice guideline on pediatric GER and GERD, jointly developed by the North American Society for Pediatric Gastroenterology, Hepatology, and Nutrition (NASPGHAN) and the European Society for Pediatric Gastroenterology, Hepatology, and Nutrition (ESPGHAN). This work represents an update of their previous joint guideline published in 2009 [15]. The second guideline identified was developed by the National Institute for Health and Care Excellence (NICE) in 2015 [8], and updated in 2019 [16]. It addresses the diagnosis and management of GERD in children and young people. The last document [14], published in 2021, was a guideline by the Society of American Gastrointestinal and Endoscopic Surgeons (SAGES), focusing on the surgical treatment of GERD in both adult and pediatric patients.

The methodological quality of the three guidelines varied. The NASPGHAN/ESPGHAN guideline was rated as low quality (AGREE II total score: 55/100; Domain 3 score: 40/100). The SAGES guideline was considered to be of moderate quality (AGREE II total score: 60/100; Domain 3 score: 59/100). The NICE guideline received the highest rating and was considered of good quality (AGREE II total score: 73/100; Domain 3 score: 72/100).

### Systematic reviews on the eight key questions

Eight systematic reviews were conducted, one for each key question. A study protocol for these systematic reviews was registered in the PROSPERO database (CRD420251041380). All reviews were conducted following Cochrane methodology [17], and reported in accordance with the PRISMA 2020 statement [18, 19]. The guideline itself was reported in accordance with the AGREE Reporting Checklist [20].

The details of the PICO inclusion criteria and the complete information about the methods of this guideline are reported in the Additional File 1 (Extended Methods for the Systematic Review).

Studies conducted in any healthcare setting and geographic location were included.

### Review process

The literature selection process was performed independently by pairs of reviewers for each key question. The first selection was based on title and abstract screening. Articles selected in this phase were subsequently assessed for eligibility. In both phases, disagreements were resolved by consensus. The quality of observational studies and systematic reviews was assessed by the JBI checklists [21], while RCTs were evaluated using the Cochrane RoB 2 tool [22]. Data for all key questions were synthesized using GRADE evidence profile tables and narrative summaries. Subgroups of at least two participating authors focused on the different PICOs and topics, reviewed the provided list of documents and related references, produced a written text on the evidence and the summary with recommendations that all authors discussed during the meetings and voted online.

### Certainty assessment

The GRADE (Grading of Recommendations Assessment, Development and Evaluation) approach [23] was applied to assess the certainty of evidence for PICO questions 2 through 7. The certainty of evidence was rated as high, moderate, low, or very low, based on the domains of risk of bias, inconsistency, indirectness, imprecision, and publication bias.

### Consensus process

Recommendations were developed through structured discussion and iterative voting, including their direction and strength. A two-round Delphi process was conducted between April and May 2025. Consensus was defined as ≥ 80% agreement or disagreement (scores 7–9 or scores 1–3 on a 9-point Likert scale).

The strength and direction of each recommendation were also explored through voting, with participants selecting one of the four predefined categories:

- Strong recommendation against
- Weak recommendation against
- Weak recommendation for
- Strong recommendation for

The final consensus on the content and strength of each recommendation was achieved through discussion during the plenary session, where all panel members participated.

A total of 40 recommendations related to PICO questions 2 through 7 were formulated and finalized through this combined process. Fourteen recommendations concerning the symptoms and diagnosis of GER and GERD in infants, children, and adolescents are presented in this paper.

### External review

An external review was conducted to ensure the validity, applicability, and clarity of both the guideline text and the recommendations. The draft guideline was shared with three pediatric external experts on GERD (Osvaldo Borrelli, Yvan Vandenplas, Mario C. Vieira) and with the Presidents of Italian Federation of Societies of Digestive Diseases (FISMAD), of the Italian Pediatric Society of Neonatology (SIN) and of Respiratory Diseases (SIMRI) and of an Association of Italian Family Pediatricians who were not involved in the development process. These included primary care and hospital pediatricians, pediatric gastroenterologists, adult gastroenterologists and representatives from relevant Italian scientific societies.

Reviewers were selected based on their clinical or methodological expertise and institutional role.

They were invited to provide comments and suggestions, focusing on the clarity, relevance, feasibility, and potential impact of each recommendation.

All feedback received was summarized and discussed during a plenary meeting of the guideline panel. Where appropriate, relevant suggestions and criticisms were incorporated into the final version of the recommendations.

### Facilitators and barriers to application

The panel considered facilitators and barriers to the implementation of the guideline recommendations.

Facilitators identified:

- Broad availability of diagnostic tools (e.g., esophageal pH-impedance, endoscopy) in referral centers.
- Existing clinical awareness of GER/GERD in pediatrics.
- High relevance and clarity of the recommendations.
- Multidisciplinary interest in standardized management approaches.

Barriers noted:

- Limited access to specialized tests in some geographic areas.
- Variability in health care professionals’ knowledge of management strategies.
- Potential overuse or misuse of pharmacological treatments.
- Lack of awareness of updated evidence among general practitioners and pediatricians.

Where barriers were identified, recommendations were worded to allow flexibility based on available resources and clinical judgment.

### Implementation tools and advice

**Fig. 1:**
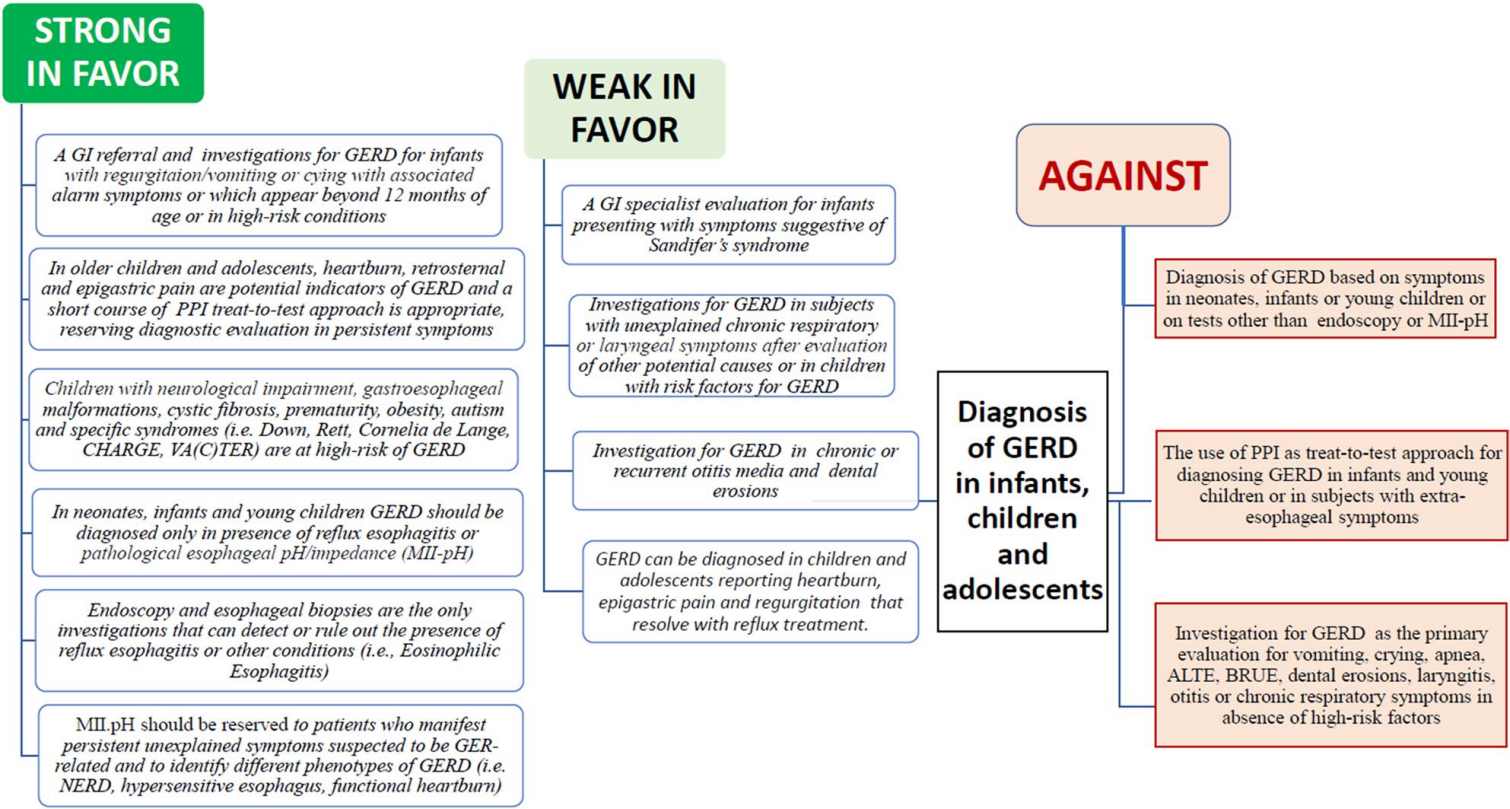
Graphic Summary of Strenght of Recommendations related to the diagnosis of GER and GERD in infants, children and adolescents

**Fig. 2:**
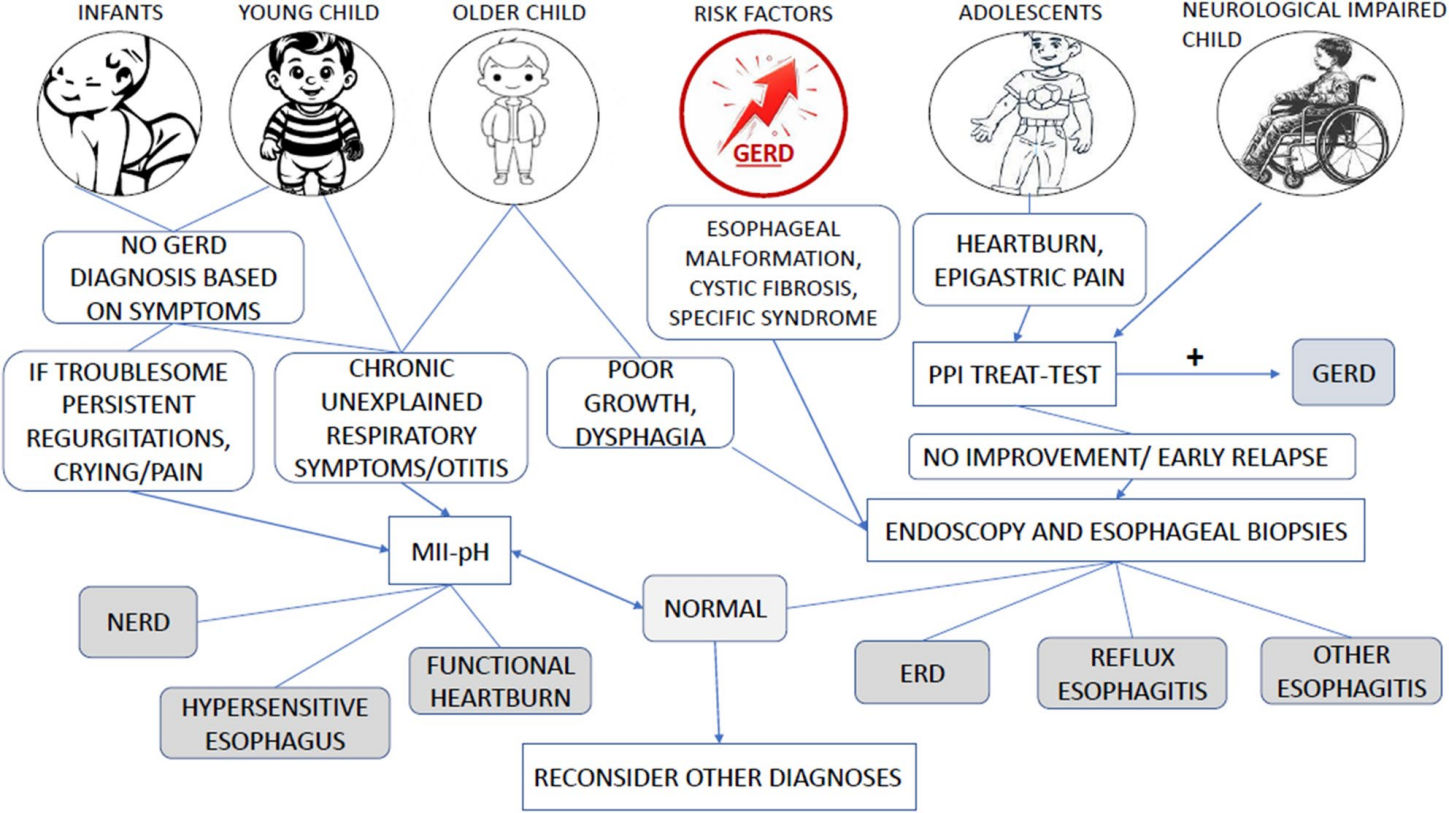
Algorithm of GERD diagnosis according to age, presenting symptoms and associated conditions

To support the practical application of this guideline, a summary table was developed (Table 2) presenting each PICO question alongside the corresponding recommendations and their strength and direction. Two figures (Figs. 1 and 2) were created to represent the diagnostic algorithm for pediatric GERD and strength of recommendations for or against different diagnostic approach in pediatric ages in line with guideline implementation standards [20]. These tools are intended to facilitate quick reference and integration of the guideline content into clinical decision-making.

**Table 2:** Recommendations concerning diagnosis of GER and GER in infants, children and adolescents

Recommendations	Strength of Recommendations
**PICO 2: What are the signs and symptoms associated with GER/GERD?**	
***Recommendation 2.1. Infants with regurgitation***,*** vomiting***,*** and crying*** *In infants*,* regurgitation/vomiting*,* or irritability are common symptoms and often resolve spontaneously by 12–14 months. GERD should be considered if regurgitation/vomiting or irritability are accompanied by alarm signs such as stunted growth*,* hematemesis*,* dysphagia*,* feeding difficulties*,* distress or symptoms persisting beyond the first year of life. In these cases*,* referral to a pediatric gastroenterologist and tailored investigations arerecommended.*	Strong in favor
***Recommendation 2.2. Infants with Apnea/ALTEs/BRUEs*** *Investigation for GERD is not recommended as the primary evaluation for apnea*,* ALTEs*,* or BRUEs unless high-risk factors are present (e.g.*,* neurological impairment*,* multiple episodes*,* prematurity*,* age < 2 months). In such cases*,* referral to a pediatric gastroenterologist is advised.*	Strong in favor
***Recommendation 2.3. Infants with Sandifer’s syndrome*** *For infants presenting with symptoms suggestive of Sandifer’s syndrome (episodic torticollis with neck extension and rotation)*,* a pediatric gastroenterologist evaluation is advised despite the rarity of the condition and limited supporting evidence.*	Weak in favor
***Recommendation*** ***2.4. Children and adolescents - Regurgitation and vomiting*** *Referral to a pediatric gastroenterologist is recommended in recurrent regurgitation/vomiting which persist or appear beyond 12 months of age to identify GERD and differentiate it from other conditions.*	Strong in favor
***Recommendation 2.5. Children and adolescents - Heartburn and epigastric*** ***pain*** *In older children and adolescents*,* symptoms such as heartburn*,* chest pain*,* and epigastric discomfort are considered as indicators of GERD. In this group*,* an empirical course of proton pump inhibitor therapy is generally appropriate*,* with diagnostic evaluation reserved for those who do not achieve adequate symptom resolution*	Strong in favor
***Recommendation 2.6. Dental erosions*** *Routine evaluation of GERD is not advised in children with dental erosions unless accompanied by risk factors or additional signs or symptoms indicative of GERD.*	Weak in favor
***Recommendation 2.7. Respiratory symptoms*** *In children with chronic cough*,* hoarseness*,* or laryngitis we suggest to consider GERD with the presence of heartburn or epigastric pain. Investigations for GERD is advised in subjects with unexplained chronic respiratory or ENT symptoms after exclusion of alternative causes or in children with risk factors for GERD such as cerebral palsy*,* prematurity*,* cystic fibrosis*,* esophageal malformations*, etc.	Weak in favor
***Recommendation 2.8. Wheezing and asthma*** *GERD investigation is not routinely advised in children with wheezing or asthma unless symptoms are refractory to standard asthma management.*	Weak in favor
***Recommendation 2.9. Otitis*** *Investigation for GERD may be considered in cases of chronic or recurrent otitis media when associated with GERD symptoms or risk factors*	Weak in favor
**PICO 3: What are the risk factors of GERD?**	
***Recommendation 3. Risk factors for GERD*** *We recommend considering at high risk of GERD infants and children with neurological impairment*,* neuromotor disorders*,* gastroesophageal malformations*,* cystic fibrosis*,* prematurity*,* obesity and specific syndromes (i.e. Down*,* Rett*,* Cornelia de Lange*,* CHARGE*,* VA(C)TERL*,* Sandifer*,* Autism Spectrum Disorders)”*	Strong in favor
**PICO 4: What is the value of different diagnostic testing for GERD in infants and children?**	
***Recommendation 4.1. Neonates***,*** Infants and Young children*** *The panel recommends diagnosingf GERD in neonates*,* infants and young children only when reflux esophagitis is detected by endoscopy or esophageal biopsy or when esophageal pH/impedance (PH-MII) monitoring is abnormal*	Strong in favor
***Recommendation 4.2. Older children and adolescents*** *GERD can be diagnosed in older children and adolescents who complain of heartburn*,* epigastric pain*,* and regurgitation that resolve with reflux treatment.*	Weak in favor
***Recommendation 4.3. Endoscopy and esophageal biopsies*** *The panel recommends considering endoscopy with esophageal biopsies as the only investigation capable of confirming or excluding reflux esophagitis and other esophageal conditions (i.e.*,* Eosinophilic Esophagitis)*	Strong in favor
***Recommendation 4.4. Esophageal pH-impedance (PH-MII)*** *The panel recommends reserving PH-MII to patients with persistent unexplained symptoms suspected to be GER-related and to identify distinct GERD phenotypes (i.e. NERD*,* hypersensitive esophagus*,* functional heartburn)*	Strong in favor

### Updating procedure

The panel has established a plan to review and update the guideline every five years, or earlier if substantial new evidence becomes available that may warrant changes to current recommendations.

## Results

### Literature searches

Each PICO had specific search strategies and identified a different number of studies. The following supplementary materials are available:

- Additional File 1: Search strategies; PRISMA flow diagrams; list of excluded studies with reasons for their exclusion; Extended Methods for the Systematic Review;
- Additional File 2: Characteristics of included studies tables;
- Additional File 3: Quality assessment tables;
- Additional File 4: GRADE Evidence Profile tables.

In total, the panel formulated 40 recommendations, all of which achieved consensus (with > 80% of panelists scoring 7–9); 26 were rated as strong in favor and 14 as weak in favor. With regard to the diagnosis of GER and GERD, 14 recommendations were developed, of which 8 were rated as strong in favor and 6 as weak in favor. (Table 2).

### Definition of GER and GERD

**Key Question 1**: **What is the definition of GER and GERD in infants**,** children**,** and adolescents?**

### Study selection

The search strategies for this question are detailed in Additional File 1. In the identification phase, 1,464 articles were retrieved from the databases. After removing 162 duplicates, 1,302 records remained for screening. During the initial screening phase, 1,263 articles were excluded—including 20 further duplicates—as they did not meet the predefined inclusion criteria for this question.

A total of 39 articles were selected for full-text assessment. Of these, 32 were excluded. Reasons for exclusion included: study focus not relevant to GER/GERD, inappropriate study design, or absence of an explicit disease definition.

### Summary of evidence

Definitions of GER and GERD were extracted from the 7 articles included [1, 16, 24–28] whose characteristics are summarized in Additional File 2. These definitions were found to be broadly consistent across studies. Based on clinical expert judgment, the following working definitions were selected:

*GER is the passage of gastric contents into the esophagus with or without regurgitation and vomiting.*

*GERD occurs when GER leads to troublesome symptoms that affect daily functioning and/or results in complications.*

These definitions were reported in 2006 by the Montreal consensus group [3] and later adopted by pediatric gastroenterology societies such as ESPGHAN and NASPGHAN [1, 15], AAP [9] and NICE guideline [8, 16].

Based on chemical content detected by pH-monitoring, GER can be classified as acid, when pH is less than 4, non-acid (also called weakly acid) if pH is between 4 and 7 or weakly alkaline reflux when pH is greater than 7 [1].

The definition of GERD in the pediatric population is subject to several limitations. In clinical practice, distinguishing GER from GERD remains problematic due to the broad spectrum and variability of reflux symptoms, the limited capacity of younger children to report them, and the subjective interpretation of what constitutes ‘troublesome’ symptoms by children, parents, and healthcare professionals. No alternative definitions have been proposed, and validation studies on this definition are lacking. In this guideline, every effort was made to use the terms GER and GERD strictly according to their meanings.

### Clinical presentation

**PICO Question 2 (Q2)**: **What are the signs and symptoms indicative of GERD?**

### Infants

### Recommendation 2.1. Infants with regurgitation, vomiting, and irritability

*In infants*,* regurgitation/vomiting*,* or irritability are common symptoms and often resolve spontaneously by 12–14 months. GERD should be considered if regurgitation/vomiting or irritability are accompanied by alarm signs such as stunted growth*,* hematemesis*,* dysphagia*,* feeding difficulties*,* distress or symptoms persisting beyond the first year of life. In these cases*,* referral to a pediatric gastroenterologist and tailored investigations are recommended.*

AGREEMENT (scores 7–9:100%); Strength of Recommendation (SOR): 90.9% strong in favor; 9.1% weak in favor.

### Recommendation 2.2. Infants with Apnea/ALTEs/BRUEs

*Investigation for GERD is not recommended as the primary evaluation for apnea*,* ALTEs*,* or BRUEs unless high-risk factors are present (e.g.*,* multiple episodes*,* prematurity*,* or being under two months old). In such cases*,* referral to a pediatric gastroenterologist is advised.*

AGREEMENT (scores 7–9:100%); SOR: 100% strong in favor.

### Recommendation 2.3. Infants with Sandifer’s syndrome

*For infants presenting with symptoms suggestive of Sandifer’s syndrome (episodic torticollis with neck extension and rotation)*,* a pediatric gastroenterologist evaluation is advised despite the rarity of the condition and limited literature evidence.*

AGREEMENT (scores 7–9:100%); SOR: 41.67% strong in favor; 58.33% weak in favor.

### Children and adolescents

#### Recommendation 2.4. Children and adolescents - Regurgitation and vomiting

*Referral to a pediatric gastroenterologist is recommended in recurrent regurgitation/vomiting which persist or appear beyond 12 months of age to identify GERD and differentiate it from other conditions.*

AGREEMENT (scores 7–9:90.9%; scores 4–6:9.1%); SOR: 91.67% strong in favor; 8.33% weak in favor.

### Recommendation 2.5. Children and adolescents - Heartburn and epigastric pain

*In older children and adolescents*,* symptoms such as heartburn*,* chest pain*,* and epigastric discomfort are considered as indicators of GERD. In this group*,* an empirical course of proton pump inhibitor therapy is generally appropriate*,* when used with caution*,* with diagnostic evaluation reserved for those who do not achieve adequate symptom resolution*.

AGREEMENT (scores 7–9:100%); SOR: 91.67% strong in favor; 8.33% weak in favor.

### Study selection

The literature search identified 4,717 records (1,349 from PubMed, 1,646 from Embase and 1,722 from Web of Science). After removing duplicates, 3,871 records were screened by title and abstract, and 3,750 were excluded for not meeting the inclusion criteria or being duplicates. One hundred seventeen full-text articles were assessed for eligibility, of which 59 studies [29–87] were included in the final analysis. The PRISMA flow diagram and the list of excluded studies, along with the reasons for exclusion, are provided in Additional File 1.

### Study characteristics

Among the included studies, 24 were case series [29–32, 34, 35, 39, 40, 42, 44, 46–49, 53–55, 57, 62, 63, 67, 72, 73, 81], 20 were cohort studies [33, 38, 41, 45, 50, 56, 58, 61, 65, 68–71, 75, 79, 82–85, 87], 7 were cross-sectional studies [37, 43, 60, 64, 74, 77, 86], one was a case-control study [66], and 7 were systematic reviews [36, 51, 52, 59, 76, 78, 80]. The studies were published between 1983 and 2023. Most studies were conducted in Europe (30.5%), North America (28.8%), and Asia (18.6%). Among primary observational studies, the median sample size was 86 patients (interquartile range: 91).

The characteristics of the included studies are detailed in Additional File 2.

### Summary of evidence

The clinical presentation of GERD varies considerably with age, which complicates the differentiation between physiologic GER and pathologic GERD, particularly in infants [76, 88]. Nevertheless, establishing an accurate diagnosis and distinguishing GERD from other conditions that may mimic reflux is crucial to guide appropriate management and prevent unnecessary pharmacological therapy. GERD may present with recurrent regurgitation/vomiting, growth faltering, excessive irritability in infants, hematemesis, dysphagia, heartburn or chest pain in children and adolescent. In addition to these gastrointestinal manifestations, a spectrum of extraesophageal manifestations has been attributed to GERD, most prominently respiratory disorders such as apnea or apparent life-threatening events (ALTEs)/brief resolved unexplained events (BRUEs) in infants, recurrent wheezing/asthma, chronic cough, recurrent otitis media, aspiration pneumonia and dental erosions. Importantly, no single symptom reliably predicts the presence of GERD, underscoring the challenge of distinguishing GER from GERD and other conditions in pediatric populations.

The significant variation in the definitions of GERD, diagnostic criteria and the outcome measures used to evaluate treatment effectiveness across different studies is worth noting. These outcomes range from symptom resolution to decreased reflux episodes during pH-MII monitoring or the healing of esophagitis. This variability complicates the comparison of findings between studies.

### Infants

Regurgitation, vomiting, and irritability are very common in healthy infants. Approximately 70% of otherwise healthy infants experience regurgitation, and about 25% regurgitate multiple times per day, particularly during the first six months of life. In the majority of cases (≈ 95%), these symptoms resolve spontaneously without therapeutic intervention by 12 months of age, as reported in both Italian and international cohorts [1, 33, 76]. As a result, neither regurgitation/vomiting, nor irritability is sufficient to diagnose GERD. However, GERD is often considered in infants with these nonspecific symptoms. The likelihood of GERD increases when these manifestations are accompanied by warning features such as impaired growth, persistent and severe irritability or inconsolable crying, feeding difficulties, hematemesis, dysphagia, or regurgitation persisting beyond the first year of life [1, 8, 15, 16]. In such cases, referral to a pediatric gastroenterologist and consideration of targeted diagnostic evaluation are warranted. In selected infants, regurgitation/vomiting, crying, feeding difficulties and poor growth may result from cow’s milk allergy, particularly when eczema or respiratory symptoms coexist [89]. A simple questionnaire based on 5 clinical items (CoMiSS) can help clinicians to identify infants who have symptoms related to cow’s milk [90].

GER has also long been implicated as a potential contributing factor in infant apnea [78, 91, 92], Apparent Life-Threatening Events (ALTEs) [93, 94] and Brief Resolved Unexplained Events (BRUEs) [30, 69]. However, the available evidence is inconsistent, relying on heterogeneous diagnostic methodologies, and has not demonstrated clear benefit of pharmacological GERD therapy in these contexts. After completing an initial pediatric assessment based on specific guidelines, referral to a pediatric gastroenterologist for further evaluation should be reserved for high-risk infants, such as those with neurological complications, recurrent episodes, prematurity, or under the age of two months.

Features of Sandifer’s syndrome (episodic torticollis with neck extension and rotation) have been historically associated with GERD and cited in prior GERD guidelines. However, the current literature addressing this relationship remains limited, largely due to the rarity of the condition.

Alarm and “Red flag” symptoms and signs for secondary GERD or other conditions are summarized in Table 3.

**Table:** 

Practice point:
There is no pathognomonic sign or symptom of GERD in infants. Regurgitation during the first months of life is frequent, may cause infant distress and is often related to overfeeding
A comprehensive evaluation to exclude alternative conditions and to identify alarm features is essential in infants with persistent or atypical symptoms

**Table 3:** Alarm and “Red flag” symptoms and signs for secondary GERD or other conditions (modified by Rosen 2018 [1] and Gonzalez Ayerbe 2019 [95])

Medical history	Physical Examination
Regurgitation in the first week of life or after 12 months of life	Abnormal abdominal, respiratory or neurological findings
Forceful vomiting with bile or blood or with nocturnal or cyclic episodes	Bulging fontanel or abnormal increase of head circumference
Chronic or bloody diarrhea	Delayed psychomotor development
Urinary problems	Abnormal muscle tone or position
Seizures	Lethargy or excessive irritability
Bolus impaction or dysphagia	Failure to thrive/weight loss
Recurrent pneumonia	Abnormal vital signs

### Children – adolescents

#### Summary of evidence

After 12 months of age, recurrent regurgitation and vomiting should prompt evaluation for GERD and alternative diagnoses. In young children, non-specific symptoms such as food refusal, dysphagia, or sleep disturbances may be considered, while in those older than 8 years and in adolescents, heartburn, chest and epigastric pain are the most suggestive symptoms of GERD. However, other conditions including eosinophilic esophagitis, functional heartburn and dyspepsia can present with similar symptoms and need a specific diagnostic work-up.

### Extra-esophageal symptoms and signs

#### Dental erosions

***Recommendation 2.6.*** *Routine evaluation of GERD is not advised in children with dental erosions unless accompanied by risk factors or additional signs or symptoms indicative of GERD.*

AGREEMENT (scores 7–9:100%); SOR: 75% strong in favor; 25% weak in favor.

#### Summary of evidence

Various studies have assessed the contribution of GERD to dental erosions in children, with conflicting results [51, 52]. Dental health outcomes are influenced by multiple confounders, including oral hygiene, dietary habits, age, and medication use, which complicate interpretation. Current evidence is therefore insufficient to recommend a referral to a gastroenterologist solely for dental erosions in the absence of additional GERD-related symptoms, signs, or risk factors.

#### Chronic respiratory and ENT symptoms

***Recommendation 2.7.*** *In children with chronic cough*,* hoarseness*,* or laryngitis we suggest to consider GERD with the presence of heartburn or epigastric pain. Investigations for GERD is advised in subjects with unexplained chronic respiratory or ENT symptoms after exclusion of alternative causes or in children with risk factors for GERD such as cerebral palsy*,* prematurity*,* cystic fibrosis*,* esophageal malformations*, etc.

AGREEMENT (scores 7–9:90.9%; scores 4–6:9.1%); SOR: 72.7% strong in favor; 27.3% weak in favor.

***Recommendation 2.8***. *GERD investigation is not routinely advised in children with wheezing or asthma unless symptoms are refractory to standard asthma management.*

AGREEMENT (scores 7–9:100%); SOR: 75% strong in favor; 25% weak in favor.

***Recommendation 2.9***. *Investigation for GERD may be considered in cases of chronic or recurrent otitis media and other GERD-related symptoms or risk factors*.

AGREEMENT (scores 7–9:100%); SOR: 58.3% strong in favor; 41.7% weak in favor.

### Summary of evidence

GERD has long been considered a causal or predisposing factor for some extra-esophageal manifestations including chronic cough, recurrent wheezing and asthma, recurrent pneumonia, recurrent otitis media, and laryngeal abnormalities [96].

GER may lead to signs and symptoms of tissue injury within the oropharynx, larynx, and respiratory tract related to the frequency, duration or content of the reflux or because of individual inefficient defensive mechanisms or hypersensitivity. Airway inflammation may result in chronic cough and symptoms of laryngeal dysfunction (hoarseness, croup, stridor) in susceptible individuals.

Evidence linking GERD to chronic cough and laryngeal symptoms in children remains conflicting [36, 97, 98]. Associations between recurrent wheezing or asthma and GER have been reported [54, 70, 99] but causality remains uncertain and treatment of GERD has not improved asthma outcomes in different cohorts of pediatric patients [96, 100]. It is worth noting that chronic cough itself may predispose to GER by increasing intra-abdominal pressure and negative intrathoracic pressure, while GER can exacerbate cough reflex sensitivity, airway inflammation, and—in severe cases—microaspiration. The prevalence of GERD varies considerably, depending on the population included and diagnostic tests applied. Before assessing GERD or starting acid suppressant treatment, in the absence of heartburn, numerous potential etiologies for chronic cough and laryngeal inflammation should be considered and evaluated, as highlighted in guidelines produced by expert pediatric pneumologists [44, 83, 87, 96, 101].

In children with unexplained or persistent symptoms, the role of both acid and non-acid reflux can be detected only by PH-MII monitoring and may impact on these conflicting results, treatment approach and outcomes. Time-relation between cough and reflux can objectively established by combined pH-impedance manometry [102] although this investigation is not widely available and requires specific expertise.

- *Recurrent pneumonia*: In vulnerable children with neuromotor disabilities, swallowing dysfunction, inefficient cough reflex or airway malformations, refluxate can penetrate into the airways leading to aspiration pneumonia. As with other respiratory disorders, the prevalence of GERD in this clinical setting varies widely. We suggest considering diagnostic testing for both swallowing abnormalities and reflux in children with recurrent pneumonia.
- *Otitis media*: Chronic or recurrent otitis media with effusion has been linked to GER and pharyngeal reflux in infants and children [8, 16, 103]. Proposed mechanisms include reflux-related nasopharyngeal inflammation or direct refluxate entry into the middle ear via immature eustachian tube anatomy. Although pepsin detection in middle-ear aspirates has been reported, its clinical relevance remains debated. Evidence for therapeutic benefit of GERD management in this setting is currently limited.

**Table:** 

Practice points
• The contribution of GERD to extraesophageal symptoms in children remains incompletely defined, largely due to heterogeneity in diagnostic criteria and study populations. • GERD should be considered in selected children with gastroesophageal malformations, neurological impairment, dental erosions, recurrent pneumonia, bronchial hyperreactivity, recurrent wheezing, difficult to treat asthma, adenoid and tonsillar hypertrophy, laryngeal abnormalities.
• A full diagnostic work-up and exclusion of alternative etiologies are essential, and GERD investigation should be performed only in cases refractory to conventional management. • Empirical PPI therapy is not recommended as a diagnostic test for GERD in infants and children with isolated extra-esophageal symptoms.

### PICO Question 3: What are the risk factors for GERD?

**Recommendation 3.1**. *We recommend considering at high risk of GERD infants and children with neurological impairment*,* neuromotor disorders*,* gastroesophageal malformations (i.e. esophageal atresia*,* tracheoesophageal fistula*,* diaphragmatic hernia)*,* cystic fibrosis*,* prematurity*,* obesity and specific syndromes (i.e. Down*,* Rett*,* Cornelia de Lange*,* CHARGE*,* VA(C)TERL*,* Sandifer*,* Autism Spectrum Disorders)”*.

AGREEMENT (scores 7–9:100%); SOR: 91.67% strong in favor; 8.33% weak in favor.

### Study selection

The literature search yielded 4,201 records (1,169 from PubMed, 1,712 from Embase, and 1,320 from Web of Science). Following removal of duplicates, two independent reviewers (LG and CLG) screened 3,603 records, of which 3,558 were excluded. After full-text evaluation of the remaining 45 studies, 18 articles [30, 47, 104–118] met the eligibility criteria. The PRISMA flow diagram is provided in Additional File 1. A list of excluded studies, along with reasons for exclusion, is also presented in Additional File 1.

### Study characteristics

Among the 18 included studies, 12 were cross-sectional studies [30, 40, 47, 105–108, 110, 113, 115–117], 4 were cohort studies [104, 109, 112, 114], one case control study [111] and one systematic review [118]. Publication years ranged from 1993 to 2021. Seven studies specifically examined risk factors for GER/GERD in infants [30, 47, 106, 107, 111–113], while the remaining focused on children older than 12 months. The most frequently investigated risk factor was prematurity [30, 47, 107, 108, 111, 113], followed by overweight and obesity [109, 110, 114] and asthma [117, 118]. A comprehensive summary of study characteristics is provided in Additional File 2.

### Summary of evidence

A wide range of risk factors have been linked to GER, including prematurity, mechanical ventilation, airway and gastroesophageal malformations (i.e. esophageal atresia, tracheoesophageal fistula, diaphragmatic hernia), neurodevelopmental disorders, neuromotor impairment, chronic respiratory symptoms, cystic fibrosis, obesity, and selected genetic syndromes (i.e. Down, Cornelia de Lange, Rett, CHARGE, VA(C)TERL Prematurity, particularly gestational age < 32 weeks or when complicated by severe respiratory, neurological, or infectious comorbidities, is associated with increased GER symptoms during early infancy [113, 119]. Multiple factors contribute to the predisposition of preterm infants to GER, including the short length of the esophagus, frequent milk feeding, prolonged supine position, delayed gastric emptying, LES immaturity and transient relaxations. Moreover, mechanical ventilation may predispose to GERD mostly by impairing LES pressure and esophageal clearance [104, 120]. Cough episodes may also exacerbate GER events by increasing abdominal pressure while nasogastric tube does not appear to significantly increase GER or acid exposure [112]. Different congenital gastroesophageal malformations and more specifically esophageal atresia, tracheoesophageal fistula, diaphragmatic hernia and gastroschisis are frequently associated with long-term sequelae, including GERD [121–124]. Post-surgical sequelae—such as anatomical alterations of the gastroesophageal junction, abnormal LES tone, altered abdominal–thoracic pressure dynamics, and esophageal dysmotility—further increase risk. A multidisciplinary approach, structured follow-up with periodic investigations and carefully managed transitional care are essential to ensure optimal care throughout pediatric ages into adulthood [121–124]. The frequency and severity of GERD in children with neurological impairment is high as symptoms may be hampered by communication difficulties and overlapping comorbidities that may lead to pain and vomiting [125]. Early-onset neurological impairment, cerebral palsy, abnormal EEG results, anti-epileptic treatment, mitochondrial disease, severe mental retardation (IQ < 35), vomiting, rumination and hematemesis represent risk factors for severe GERD [126, 127]. In addition, anxiety or depression can increase individual perception and sensitivity of reflux symptoms. Conversely, GERD-related symptoms may exacerbate sleep disturbances, behavioral issues, and quality of life deterioration [116]. GERD is reported in 1/3 to 1/2 of patients with cystic fibrosis (CF). Both GERD and the use of acid-suppressive therapy have been implicated in adverse pulmonary outcomes, including impaired lung function and increased exacerbations. Nonetheless, GERD can also occur as a consequence of respiratory problems, frequent and persistent cough, delayed gastric emptying, dysunction of lower esophageal sphincter dysfunction and impaired esophageal motility of CF patients [128–130]. High body mass index and obesity are also associated with elevated GERD risk, though pediatric evidence remains limited and primarily based on questionnaire-derived symptom reporting rather than endoscopic findings [76, 131, 132].

Although comprehensive discussion of each condition is beyond the scope of this guideline, the working group highlighted the need to enhance the quality of care for these complex pediatric populations. A dedicated section with specific practice points is therefore included in the subsequent part of this guideline.

**Table:** 

Practice point
Children with recurrent regurgitation beyond infancy, severe neurological impairment, prematurity, congenital gastroesophageal malformations, cystic fibrosis, obesity, or specific genetic syndromes
should undergo careful evaluation and ongoing monitoring for GERD

#### Diagnostic testing

**PICO Question 4**: **What is the value of different diagnostic testing for GERD in infants and children?**

***Recommendations 4.1.*** *The panel recommends diagnosing GERD in neonates*,* infants and young children only when reflux esophagitis is detected by endoscopy or esophageal biopsy or when esophageal pH/impedance (PH-MII) monitoring is abnormal*.

AGREEMENT (scores 7–9:100%); SOR: 100% strong in favor.

***Recommendations 4.2.*** *GERD can be diagnosed in older children and adolescents who complain of heartburn*,* epigastric pain*,* and regurgitation that resolve with reflux treatment.*

AGREEMENT (scores 7–9:91.67%; scores 4–6:8.33%); SOR: 75% strong in favor; 25% weak in favor.

#### Recommendations 4.3 The panel recommends endoscopy with esophageal biopsies as the sole investigation capable of detecting or excluding reflux esophagitis or alternative conditions (i.e., Eosinophilic Esophagitis)

AGREEMENT (scores 7–9:100%); SOR: 90.9% strong in favor; 9.1% weak in favor.

### Recommendations 4.4 The panel recommends reserving PH-MII to patients with persistent unexplained symptoms suspected to be GER-related and to identify distinct GERD phenotypes (i.e. NERD, hypersensitive esophagus, functional heartburn)

AGREEMENT (scores 7–9:90.9%; scores 4–6:8.1%); SOR: 81.8% strong in favor; 18.2% weak in favor.

### Study selection

The systematic review identified 4,036 records (1,390 from PubMed, 1,570 from Embase, and 1,076 from Web of Science). After removal of duplicates, 3,648 records were evaluated by two reviewers (MO and LG), who excluded 3,549. Following full-text analysis of the remaining records resulted in the inclusion of, 39 publications [77, 133–170] were selected. The Prisma flow diagram is available in Additional File 1. A list of excluded studies and the reasons for exclusion is also provided in Additional File 1.

### Study characteristics

Of the 39 included studies, 26 were diagnostic accuracy investigations, 6 were cross-sectional studies [77, 134, 137, 141, 145, 157], 6 were cohort studies [133, 148, 154, 159, 160, 170], and one was a case series [164]. The papers were published between 1980 and 2022. Thirteen focused on GER/GERD in infants [133, 135, 139, 142, 146, 152, 153, 155, 161, 163, 164, 167, 168], whereas the remainder were conducted in pediatric populations > 12 months of age. There was a wide variability of investigations for GERD across the studies including 24 h esophageal pH-(MII) monitoring (the one most commonly performed), upper gastrointestinal contrast series, ultrasound, scintigraphy, upper endoscopy with biopsies, salivary analysis (acid detection, salivary pepsin test, radionuclide salivagram, ELISA pepsin score), Lipid-laden macrophage index, and electric impedance tomography. A comprehensive table of study characteristics is provided in Additional File 2.

### Summary of evidence

European and American pediatric GERD guidelines emphasize that diagnosis in children should rely on either (i) symptom-based criteria (heartburn or epigastric pain in children > 8 years capable of reliable reporting) or (ii) objective evidence of pathological reflux on PH-MII monitoring or reflux-related esophagitis [1, 9, 171]. Several studies demonstrate that, in infants and young children, no symptom, cluster of symptoms or questionnaire reliably predict the response to acid-suppressive therapy or correlates with the findings on endoscopy or PH-MII [1, 9, 171]. Endoscopy (with esophageal biopsies) and PH-MII -monitoring are complementary diagnostic investigations that provide different information.

Endoscopy is the only test that allows the direct visual examination of the esophageal mucosa, the identification of macroscopic lesions associated with GERD and the possibility to perform biopsies that can detect and characterize esophagitis. It should be performed in selected patients with esophageal malformation or surgical intervention (i.e. esophageal atresia), failure to thrive, hematemesis, unexplained anaemia or dysphagia and in persistent gastroesophageal symptoms.

PH-MII monitoring provides quantitative assessment of reflux episodes (acidic, weakly acidic, or alkaline), characterization of reflux events and their temporal association with symptoms [158, 172–174]. Advances in automated analysis and the establishment of different pediatric [175–177] have improved its diagnostic utility. In addition, expert manual reading of tracings and evaluation of esophageal baseline values may provide indirect information about the possible risk of esophagitis or motility disorders, aiding in the selection of patients for endoscopy or manometry [160, 178, 179].

By contrasts, using pH-monitoring without the impedance detects only acid reflux, limiting diagnostic accuracy.

Previous guidelines and position papers have reported a detailed description of these two investigations, indications and interpretation of the different parameters [174, 178].

Other diagnostic modalities—including oropharyngeal pH-monitoring, esophageal ultrasound, and barium contrast studies—exhibit low sensitivity and specificity, lack standardized reference values, and are not recommended for pediatric GERD diagnosis [1, 9, 171, 180–182]. Similarly, the diagnosis of GERD cannot be established based on the results of scintigraphy [183], salivary pepsin [184], laryngeal fibroscopy [97, 98]. Notably, manometry is reserved for patients with suspected motility disorders that can predispose or result from GERD [1].

#### Infants

In infants, multiple studies and systematic reviews have demonstrated a poor correlation between symptoms such as crying/fussiness, regurgitation, apnoea/desaturation, laryngeal alteration, chronic cough, feeding or sleep disorders and the presence of esophagitis or pathological esophageal acid reflux as detected by esophageal pH(MII) monitoring [88, 92, 185, 186]. The Infant Gastroesophageal Reflux Questionnaire (I-GERQ) and its revised version (I-GERQ-R) are the only validated symptom-based tools [187] but their sensitivity and specificity for GERD objectively diagnosed by endoscopy and/or pH(MII)-monitoring are suboptimal [161, 188]. Hence, empirical PPI treatment in this age group is not supported by evidence of efficacy and is also discouraged due to possible adverse effects, including an increased risk of gastrointestinal and respiratory infections in infants and young children and necrotizing enterocolitis in neonates [189, 190].

In neonates and infants with recurrent unexplained desaturation/apnoea or ALTE/BRUE a combined polysomnography PH-MII is recommended to assess reflux episodes, their temporal association with symptoms and type of reflux (acidic, weakly acidic or alkaline) in order to identify infants who need reflux treatment [93].

#### Children and adolescents

According to ESPGHAN-NASPGHAN and NICE guidelines, an initial 2–4 weeks empirical trial of PPI as a diagnostic test for GERD is recommended only in children who complain of heartburn or epigastric pain. Endoscopy should be performed in patients who fail to respond to treatment to rule out and identify the type of esophagitis [1, 171]. If the endoscopy and biopsies are normal, PH-MII may help differentiating among non-erosive reflux disease (NERD: children with pathological esophageal acid exposure), hypersensitive esophagus (children with positive symptom-reflux association), and functional heartburn (children with complete normal investigation) [1]. These different clinical phenotypes have been reported in both adults and in children, and their diferentiation is relevant for guiding individualized management strategies [191].

In children who do not report heartburn, investigation with endoscopy and/or PH-MII is recommended before starting treatment with acid suppressants. In children with severe neurological disability, an empirical treatment with PPI is suggested, given the high prevalence of severe GERD and the difficulty in reporting symptoms [125]. However, in this high-risk population for complications, it is recommended to carefully monitor improvement and perform investigations in case of persistent manifestations [125].

Combined esophageal manometry with PH-MII is currently technically feasible and may provide objective evidence of a cough-reflux association or predict response to surgery [102]. However, this complex investigation is limited in availability in many centers due to high costs of equipment and the requirement for specialized expertise. For patients being considered for surgical treatment, a complete work-up assessment for GERD, anatomical malformation and motility disorders is essential.

This guideline clarifies the essential role of endoscopy with biopsies and pH-MII in diagnosing and characterizing GERD in infants and children, incorporates extra-esophageal manifestations and at-risk conditions into the diagnostic algorithm, and provides clinically oriented practice points approved by a multidisciplinary committee. Tests recommended to diagnose GERD in neonates, infants and children are summarized in Table 4 and in Fig. 1.

**Table 4:** Tests recommended to diagnose GERD in neonates, infants and children

Tests	Indication	Strengths	Limitations
24 h esophageal pH-monitoring	To identify pathological acid GERD	Provides quantitative assessment of esophageal acid exposure during both day and night. Allows evaluation of temporal associations between reflux episodes and symptom occurrence during the recording period.	Involves costs related to pH-monitoring equipment and disposable catheters. Detects only acid reflux, without information on non-acid reflux or esophageal mucosal status. No information about non -acid reflux and esophageal mucosa.
24 h esophageal pH-MII monitoring	To identify pathological acid and non-acid GERD	Measures both acid and non-acid reflux over 24 h. Assesses temporal association between symptoms and both acid and non-acid reflux episodes. Identifies swallowing events, mean bolus clearance time, proximal extent of reflux, and the gas/liquid composition of reflux. Provides mean esophageal impedance values. Enables differentiation of distinct non-erosive reflux disease (NERD) phenotypes.	Associated with equipment and catheter costs. Requires substantial expertise for accurate manual interpretation of tracings. Pediatric normative reference values are limited. Does not provide information on esophageal mucosal integrity.
Upper endoscopy	To identify and characterize esophagitis	Allows direct visualization of the esophageal mucosa and assessment of macroscopic lesions. Enables histological evaluation through biopsy, essential for diagnosing reflux esophagitis and differentiating other conditions such as eosinophilic esophagitis. Provides information on gastric mucosa, presence of hiatal hernia, and duodenal abnormalities.	Requires specialized expertise, particularly in infants and children. Necessitates general anesthesia in pediatric patients. Does not provide data on temporal symptom–reflux association or on the chemical composition of refluxate. Interpretation of esophagitis subtypes requires expert pathological evaluation.
Esophageal biopsies	To identify and characterize histological esophagitis	It enables the identification of the presence and severity of reflux esophagitis, as well as other forms of esophagitis, such as eosinophilic esophagitis	Requires endoscopic examination with appropriate sampling, in addition to specialized expertise for accurate histopathological characterization of the esophagitis subtype. Does not provide information regarding the composition of the refluxate.

**Table:** 

Practice points
• In neonates, infants and young children, GERD should be diagnosed only in the presence of reflux esophagitis detected by endoscopy and esophageal biopsies or when esophageal pH/impedance monitoring is abnormal
• In children and adolescents, GERD can be diagnosed when heartburn and documented response to reflux treatment is reported.
• In children with severe neurological impairment an empirical PPI test can be considered reserving investigations when no improvement is reported.
• Endoscopy is the only investigation that can detect and characterize esophagitis.
• PH-MII should be reserved for patients who manifest persistent unexplained symptoms suspected to be reflux-related and is helpful to identify different phenotypes of GERD.

## Conclusions

This guideline provides 14 evidence-based recommendations and practice points derived from updated systematic reviews and expert consensus, to support clinicians properly diagnosing GER and GERD in the pediatric population. In infants, daily episodes of regurgitation are frequent in the first months of life and, in most cases, resolve spontaneously or following dietary modification. No single symptom is specific for GERD in infants and young children. Furthermore, apnoea, chronic cough, respiratory infections, recurrent wheezing, asthma, laryngitis, dental erosions, otitis are rarely caused by GERD.

Contrary to the 2018 ESPGHAN–NASPGHAN algorithm in infants, this guideline does not recommend the empirical use of PPIs and, in this age group, reserves this treatment for reflux esophagitis or acid GERD documented by pH-MII. In older children and adolescents reporting typical heartburn, or in those with severe neurological impairment, a time-limited empirical trial of PPI therapy may be considered as an initial diagnostic approach, with further testing reserved for non-responders. For children with persistent esophageal or extra-esophageal symptoms, esophageal pH-MII monitoring and endoscopy are indicated to detect and characterize reflux, phenotypes of GERD and esophagitis. A high index of suspicion of GERD should be maintained in infants and children with esophageal malformations, severe neurological impairment, cystic fibrosis or specific syndromes. These subjects require multidisciplinary evaluation that prompt action to confirm or exclude GERD, enabling timely management and prevention or early identification of complications.

**Appendix 1:** Guideline Development Group

Name	Role	Area of Expetise	Institution	City, Country
Barbara Polistena	Evidence Review Team	Methodology / Evidence Synthesis	C.R.E.A. Sanità (Centre for Applied Economic Research in Healthcare)	Rome, Italy
Massimiliano Orso	Evidence Review Team	Methodology / Evidence Synthesis	C.R.E.A. Sanità (Centre for Applied Economic Research in Healthcare)	Rome, Italy
Liliana Guadagni	Evidence Review Team	Methodology / Evidence Synthesis	Department of Surgical and Biomedical Sciences, University of Perugia	Perugia, Italy
Claudio Lo Giudice	Evidence Review Team	Methodology / Evidence Synthesis	C.R.E.A. Sanità (Centre for Applied Economic Research in Healthcare)	Rome, Italy
Ferdinando Verneau	Evidence Review Team	Methodology / Evidence Synthesis	C.R.E.A. Sanità (Centre for Applied Economic Research in Healthcare)	Rome, Italy
Gaetano Caforio	Evidence Review Team	Methodology / Evidence Synthesis	C.R.E.A. Sanità (Centre for Applied Economic Research in Healthcare)	Rome, Italy

All panel members participated in at least one plenary meeting and contributed to the formulation, discussion, and/or voting of recommendations. Members of the Evidence Review Team conducted literature searches, data extraction, and GRADE assessments.

## Supplementary Information

Below is the link to the electronic supplementary material.

Supplementary Material 1

Supplementary Material 2

Supplementary Material 3

Supplementary Material 4

Supplementary Material 5

## Data Availability

Not applicable.
