# Supplementary material for: Guidelines on diagnosis and management of gastroesophageal reflux disease in infants, children and adolescents: a joint consensus from Italian pediatric societies (SIP and SIGENP) -part I. diagnosis
Source: Ital J Pediatr. 2026 Apr 11;52:91. doi: 10.1186/s13052-026-02218-5 (PMC13185181; doi:10.1186/s13052-026-02218-5)
Supplement: Supplementary file 1 — Supplementary Material 1 [file 13052_2026_2218_MOESM1_ESM.docx]

# Additional File 1

## Search strategy

### Search strategy to identify guidelines and consensus statements on gastroesophageal reflux (GER) and gastroesophageal reflux disease (GERD) in infants, children, and adolescents

|  | PubMed, 22/02/2024 |  |
| --- | --- | --- |
| Search | Query | Results |
| #1 | guideline[Title] OR guidelines[Title] OR consensus[Title] OR recommendation[Title] OR recommendations[Title] OR "guideline"[Publication Type] OR "practice guideline"[Publication Type] OR "consensus development conference"[Publication Type] | 194,681 |
| #2 | gastroesophageal reflux[Title/Abstract] OR gastro esophageal reflux[Title/Abstract] OR gastro-oesophageal reflux[Title/Abstract] OR GERD[Title/Abstract] OR GORD[Title/Abstract] | 30,097 |
| #3 | child OR children OR pediatric OR pediatrics OR paediatric OR paediatrics OR infant OR infants OR newborn OR newborns OR adolescent OR adolescents OR juvenile OR youth OR toddler OR toddlers OR kid OR kids OR baby OR babies OR teen OR teens OR preteen OR preteens OR neonate OR neonates OR (allchild[Filter] OR newborn[Filter] OR allinfant[Filter] OR infant[Filter] OR child[Filter] OR adolescent[Filter] OR preschoolchild[Filter]) | 5,464,813 |
| #4 | #1 AND #2 AND #3 | 126 |
| #5 | #1 AND #2 AND #3 Filters: English, from 2000 - 2024 | 97 |

|  | Embase, 22/02/2024 |  |
| --- | --- | --- |
| Search | Query | Results |
| #1 | guideline:ti OR guidelines:ti OR consensus:ti OR recommendation:ti OR recommendations:ti | 224,578 |
| #2 | (gastroesophageal reflux):ti,ab OR (gastro esophageal reflux):ti,ab OR (gastro-oesophageal reflux):ti,ab OR GERD:ti,ab OR GORD:ti,ab | 50,728 |
| #3 | child OR children OR pediatric OR pediatrics OR paediatric OR paediatrics OR infant OR infants OR newborn OR newborns OR adolescent OR adolescents OR juvenile OR youth OR toddler OR toddlers OR kid OR kids OR baby OR babies OR teen OR teens OR preteen OR preteens OR neonate OR neonates | 6,294,944 |
| #4 | #1 AND #2 AND #3 | 145 |
| #5 | #1 AND #2 AND #3 AND [english]/lim AND [2000-2024]/py | 110 |

|  | Web of Science, 22/02/2024 |  |
| --- | --- | --- |
| Search | Query | Results |
| #1 | TI=(guideline OR guidelines OR consensus) | 165,848 |
| #2 | TS=(gastroesophageal reflux OR gastro esophageal reflux OR gastro-oesophageal reflux OR GERD OR GORD) | 41,240 |
| #3 | TS=(child OR children OR pediatric OR pediatrics OR paediatric OR paediatrics OR infant OR infants OR newborn OR newborns OR adolescent OR adolescents OR juvenile OR youth OR toddler OR toddlers OR kid OR kids OR baby OR babies OR teen OR teens OR preteen OR preteens OR neonate OR neonates) | 3,118,367 |
| #4 | #1 AND #2 AND #3 | 103 |
| #5 | (#1 AND #2 AND #3) AND PY=(2000-2024) AND LA=(English) | 93 |

| Google Scholar, 22/02/2024 | | |
| --- | --- | --- |
| Search | Query | Results |
| #1 | (guideline OR guidelines OR consensus) AND (gastroesophageal reflux OR gastro esophageal reflux OR gastro-oesophageal reflux OR GERD OR GORD) AND (child OR children OR pediatric OR pediatrics OR paediatric OR paediatrics OR infant OR infants OR newborn OR newborns OR adolescent OR adolescents OR juvenile OR youth OR toddler OR toddlers OR kid OR kids OR baby OR babies OR teen OR teens OR preteen OR preteens OR neonate OR neonates) | first 50 results |

### PICO 1– What is the definition of gastroesophageal reflux (GER) and gastroesophageal reflux disease (GERD) in infants, children, and adolescents?

| PubMed, 11/05/2024 | | |
| --- | --- | --- |
| Search | Query | Results |
| #1 | gastroesophageal reflux OR gastro esophageal reflux OR gastrooesophageal reflux OR gastro oesophageal reflux OR GER OR GERD OR GOR OR GORD OR (gastric[Title/Abstract] AND (acid[Title/Abstract] OR reflux[Title/Abstract])) OR (reflux[Title/Abstract] AND (oesophagitis[Title/Abstract] OR esophagitis[Title/Abstract])) OR (erosive[Title/Abstract] AND (oesophag*[Title/Abstract] OR esophag*[Title/Abstract])) OR (regurgitation[Title/Abstract] NOT (aortic[Title/Abstract] OR mitral[Title/Abstract] OR tricuspid[Title/Abstract] OR valve[Title/Abstract] OR valvular[Title/Abstract] OR paravalvular[Title/Abstract] OR pulmonary [Title/Abstract])) OR "acid reflux"[Title/Abstract] | 95,567 |
| #2 | child OR children OR pediatric OR pediatrics OR paediatric OR paediatrics OR infant OR infants OR infancy OR newborn OR newborns OR adolescent OR adolescents OR juvenile OR youth OR toddler OR toddlers OR kid OR kids OR boy OR boys OR girl OR girls OR baby OR babies OR teen OR teens OR preteen OR preteens OR teenager OR teenagers OR pubescen* OR prepubescen* OR neonate OR neonates OR (allchild[Filter] OR newborn[Filter] OR allinfant[Filter] OR infant[Filter] OR child[Filter] OR adolescent[Filter] OR preschoolchild[Filter]) | 6,804,298 |
| #3 | definition OR taxonomy OR terminology OR glossary | 1,624,417 |
| #4 | #1 AND #2 AND #3 | 1,134 |
| #5 | #1 AND #2 AND #3 AND english[Filter] | 998 |

| Embase, 11/05/2024 | | |
| --- | --- | --- |
| Search | Query | Results |
| #1 | 'gastroesophageal reflux'/exp OR 'gastroesophageal reflux':ti,ab,kw OR 'gastro esophageal reflux':ti,ab,kw OR 'gastrooesophageal reflux':ti,ab,kw OR 'gastro oesophageal reflux':ti,ab,kw OR ger:ti,ab,kw OR gerd:ti,ab,kw OR gor:ti,ab,kw OR gord:ti,ab,kw OR (gastric:ti,ab,kw AND (acid:ti,ab,kw OR reflux:ti,ab,kw)) OR (reflux:ti,ab,kw AND (oesophagitis:ti,ab,kw OR esophagitis:ti,ab,kw)) OR (erosive:ti,ab,kw AND (oesophag*:ti,ab,kw OR esophag*:ti,ab,kw)) OR (regurgitation:ti,ab,kw NOT (aortic:ti,ab,kw OR mitral:ti,ab,kw OR tricuspid:ti,ab,kw OR valve:ti,ab,kw OR valvular:ti,ab,kw OR paravalvular:ti,ab,kw OR pulmonary:ti,ab,kw)) OR 'acid reflux':ti,ab,kw | 143,723 |
| #2 | child:ti,ab,kw OR children:ti,ab,kw OR pediatric:ti,ab,kw OR pediatrics:ti,ab,kw OR paediatric:ti,ab,kw OR paediatrics:ti,ab,kw OR infant:ti,ab,kw OR infants:ti,ab,kw OR infancy:ti,ab,kw OR newborn:ti,ab,kw OR newborns:ti,ab,kw OR adolescent:ti,ab,kw OR adolescents:ti,ab,kw OR juvenile:ti,ab,kw OR youth:ti,ab,kw OR toddler:ti,ab,kw OR toddlers:ti,ab,kw OR kid:ti,ab,kw OR kids:ti,ab,kw OR boy:ti,ab,kw OR boys:ti,ab,kw OR girl:ti,ab,kw OR girls:ti,ab,kw OR baby:ti,ab,kw OR babies:ti,ab,kw OR teen:ti,ab,kw OR teens:ti,ab,kw OR preteen:ti,ab,kw OR preteens:ti,ab,kw OR teenager:ti,ab,kw OR teenagers:ti,ab,kw OR pubescen*:ti,ab,kw OR prepubescen*:ti,ab,kw OR neonate:ti,ab,kw OR neonates:ti,ab,kw | 3,532,866 |
| #3 | definition* OR taxonom* OR terminolog* OR glossar* | 475,000 |
| #4 | #1 AND #2 AND #3 | 309 |
| #5 | #1 AND #2 AND #3 AND [english]/lim | 280 |

| Web of Science, 11/05/2024 | | |
| --- | --- | --- |
| Search | Query | Results |
| #1 | TS=(gastroesophageal reflux OR gastro esophageal reflux OR gastrooesophageal reflux OR gastro oesophageal reflux OR GER OR GERD OR GOR OR GORD OR (gastric AND (acid OR reflux)) OR (reflux AND (oesophagitis OR esophagitis)) OR (erosive AND (oesophag* OR esophag*)) OR (regurgitation NOT (aortic OR mitral OR tricuspid OR valve OR valvular OR paravalvular OR pulmonary )) OR "acid reflux") | 97,816 |
| #2 | TS=(child OR children OR pediatric OR pediatrics OR paediatric OR paediatrics OR infant OR infants OR infancy OR newborn OR newborns OR adolescent OR adolescents OR juvenile OR youth OR toddler OR toddlers OR kid OR kids OR boy OR boys OR girl OR girls OR baby OR babies OR teen OR teens OR preteen OR preteens OR teenager OR teenagers OR pubescen* OR prepubescen* OR neonate OR neonates) | 3,331,552 |
| #3 | ALL=(definition* OR taxonom* OR terminolog* OR glossar*) | 827,822 |
| #4 | #1 AND #2 AND #3 | 189 |
| #5 | #1 AND #2 AND #3 and English (Languages) | 179 |

### PICO 2– What are the signs and symptoms indicative of GER and GERD in infants, children, and adolescents?

| PubMed, 11/05/2024 | | |
| --- | --- | --- |
| Search | Query | Results |
| #1 | gastroesophageal reflux OR gastro esophageal reflux OR gastrooesophageal reflux OR gastro oesophageal reflux OR GER OR GERD OR GOR OR GORD OR (gastric[Title/Abstract] AND (acid[Title/Abstract] OR reflux[Title/Abstract])) OR (reflux[Title/Abstract] AND (oesophagitis[Title/Abstract] OR esophagitis[Title/Abstract])) OR (erosive[Title/Abstract] AND (oesophag*[Title/Abstract] OR esophag*[Title/Abstract])) OR (regurgitation[Title/Abstract] NOT (aortic[Title/Abstract] OR mitral[Title/Abstract] OR tricuspid[Title/Abstract] OR valve[Title/Abstract] OR valvular[Title/Abstract] OR paravalvular[Title/Abstract] OR pulmonary [Title/Abstract])) OR "acid reflux"[Title/Abstract] | 95,570 |
| #2 | child OR children OR pediatric OR pediatrics OR paediatric OR paediatrics OR infant OR infants OR infancy OR newborn OR newborns OR adolescent OR adolescents OR juvenile OR youth OR toddler OR toddlers OR kid OR kids OR boy OR boys OR girl OR girls OR baby OR babies OR teen OR teens OR preteen OR preteens OR teenager OR teenagers OR pubescen* OR prepubescen* OR neonate OR neonates OR (allchild[Filter] OR newborn[Filter] OR allinfant[Filter] OR infant[Filter] OR child[Filter] OR adolescent[Filter] OR preschoolchild[Filter]) | 6,804,615 |
| #3 | ((sign[Title/Abstract] OR signs[Title/Abstract] OR symptom[Title/Abstract] OR symptoms[Title/Abstract] OR complaint[Title/Abstract] OR complaints[Title/Abstract]) OR ((clinical[Title/Abstract] AND (manifestation[Title/Abstract] OR manifestations[Title/Abstract] OR feature[Title/Abstract] OR features[Title/Abstract] OR finding[Title/Abstract] OR findings[Title/Abstract] OR aspect[Title/Abstract] OR aspects[Title/Abstract] OR marker[Title/Abstract] OR markers[Title/Abstract])))) OR (heartburn[Title/Abstract] OR "difficulty swallowing"[Title/Abstract] OR nausea[Title/Abstract] OR "chronic cough"[Title/Abstract] OR bloating[Title/Abstract] OR "refusing to feed"[Title/Abstract] OR regurgitation[Title/Abstract] OR (("upper abdominal"[Title/Abstract] OR chest[Title/Abstract]) AND pain[Title/Abstract]) OR "sore throat"[Title/Abstract] OR wheezing[Title/Abstract] OR dysphagia[Title/Abstract] OR laryngitis[Title/Abstract] OR vomiting[Title/Abstract] OR "weight loss"[Title/Abstract] OR pneumonia[Title/Abstract] OR "dental erosion"[Title/Abstract] OR "dental erosions"[Title/Abstract] OR "otitis media"[Title/Abstract] OR BRUE[Title/Abstract] OR "Brief Resolved Unexplained Event"[Title/Abstract] OR ALTE[Title/Abstract] OR "Apparent Life-Threatening Event"[Title/Abstract] OR apnea[Title/Abstract] OR apnoea[Title/Abstract] OR Sandifer[Title/Abstract]) | 3,309,727 |
| #4 | #1 AND #2 AND #3 | 9,992 |
| #5 | #1 AND #2 AND #3 AND ((clinicalstudy[Filter] OR meta-analysis[Filter] OR multicenterstudy[Filter] OR observationalstudy[Filter] OR randomizedcontrolledtrial[Filter] OR systematicreview[Filter]) AND (humans[Filter]) AND (english[Filter])) | 1,349 |

| Embase, 11/05/2024 | | |
| --- | --- | --- |
| Search | Query | Results |
| #1 | 'gastroesophageal reflux'/exp OR 'gastroesophageal reflux':ti,ab,kw OR 'gastro esophageal reflux':ti,ab,kw OR 'gastrooesophageal reflux':ti,ab,kw OR 'gastro oesophageal reflux':ti,ab,kw OR ger:ti,ab,kw OR gerd:ti,ab,kw OR gor:ti,ab,kw OR gord:ti,ab,kw OR (gastric:ti,ab,kw AND (acid:ti,ab,kw OR reflux:ti,ab,kw)) OR (reflux:ti,ab,kw AND (oesophagitis:ti,ab,kw OR esophagitis:ti,ab,kw)) OR (erosive:ti,ab,kw AND (oesophag*:ti,ab,kw OR esophag*:ti,ab,kw)) OR (regurgitation:ti,ab,kw NOT (aortic:ti,ab,kw OR mitral:ti,ab,kw OR tricuspid:ti,ab,kw OR valve:ti,ab,kw OR valvular:ti,ab,kw OR paravalvular:ti,ab,kw OR pulmonary:ti,ab,kw)) OR 'acid reflux':ti,ab,kw | 143,750 |
| #2 | child:ti,ab,kw OR children:ti,ab,kw OR pediatric:ti,ab,kw OR pediatrics:ti,ab,kw OR paediatric:ti,ab,kw OR paediatrics:ti,ab,kw OR infant:ti,ab,kw OR infants:ti,ab,kw OR infancy:ti,ab,kw OR newborn:ti,ab,kw OR newborns:ti,ab,kw OR adolescent:ti,ab,kw OR adolescents:ti,ab,kw OR juvenile:ti,ab,kw OR youth:ti,ab,kw OR toddler:ti,ab,kw OR toddlers:ti,ab,kw OR kid:ti,ab,kw OR kids:ti,ab,kw OR boy:ti,ab,kw OR boys:ti,ab,kw OR girl:ti,ab,kw OR girls:ti,ab,kw OR baby:ti,ab,kw OR babies:ti,ab,kw OR teen:ti,ab,kw OR teens:ti,ab,kw OR preteen:ti,ab,kw OR preteens:ti,ab,kw OR teenager:ti,ab,kw OR teenagers:ti,ab,kw OR pubescen*:ti,ab,kw OR prepubescen*:ti,ab,kw OR neonate:ti,ab,kw OR neonates:ti,ab,kw | 3,532,866 |
| #3 | sign:ti,ab,kw OR signs:ti,ab,kw OR symptom:ti,ab,kw OR symptoms:ti,ab,kw OR complaint:ti,ab,kw OR complaints:ti,ab,kw OR (clinical:ti,ab,kw AND (manifestation:ti,ab,kw OR manifestations:ti,ab,kw OR feature:ti,ab,kw OR features:ti,ab,kw OR finding:ti,ab,kw OR findings:ti,ab,kw OR aspect:ti,ab,kw OR aspects:ti,ab,kw OR marker:ti,ab,kw OR markers:ti,ab,kw)) OR heartburn:ti,ab,kw OR 'difficulty swallowing':ti,ab,kw OR nausea:ti,ab,kw OR 'chronic cough':ti,ab,kw OR bloating:ti,ab,kw OR 'refusing to feed':ti,ab,kw OR regurgitation:ti,ab,kw OR (('upper abdominal':ti,ab,kw OR chest:ti,ab,kw) AND pain:ti,ab,kw) OR 'sore throat':ti,ab,kw OR wheezing:ti,ab,kw OR dysphagia:ti,ab,kw OR laryngitis:ti,ab,kw OR vomiting:ti,ab,kw OR 'weight loss':ti,ab,kw OR pneumonia:ti,ab,kw OR 'dental erosion':ti,ab,kw OR 'dental erosions':ti,ab,kw OR 'otitis media':ti,ab,kw OR brue:ti,ab,kw OR 'brief resolved unexplained event':ti,ab,kw OR alte:ti,ab,kw OR 'apparent life-threatening event':ti,ab,kw OR apnea:ti,ab,kw OR apnoea:ti,ab,kw OR sandifer:ti,ab,kw | 4,875,143 |
| #4 | #1 AND #2 AND #3 | 10,087 |
| #5 | #1 AND #2 AND #3 AND [english]/lim AND [humans]/lim AND ('case control study'/de OR 'cohort analysis'/de OR 'cross sectional study'/de OR 'longitudinal study'/de OR 'meta analysis'/de OR 'multicenter study'/de OR 'observational study'/de OR 'prospective study'/de OR 'retrospective study'/de OR 'systematic review'/de) AND ([article]/lim OR [article in press]/lim OR [review]/lim) | 1,646 |

| Web of Science, 11/05/2024 | | |
| --- | --- | --- |
| Search | Query | Results |
| #1 | TS=(gastroesophageal reflux OR gastro esophageal reflux OR gastrooesophageal reflux OR gastro oesophageal reflux OR GER OR GERD OR GOR OR GORD OR (gastric AND (acid OR reflux)) OR (reflux AND (oesophagitis OR esophagitis)) OR (erosive AND (oesophag* OR esophag*)) OR (regurgitation NOT (aortic OR mitral OR tricuspid OR valve OR valvular OR paravalvular OR pulmonary )) OR "acid reflux") | 97,816 |
| #2 | TS=(child OR children OR pediatric OR pediatrics OR paediatric OR paediatrics OR infant OR infants OR infancy OR newborn OR newborns OR adolescent OR adolescents OR juvenile OR youth OR toddler OR toddlers OR kid OR kids OR boy OR boys OR girl OR girls OR baby OR babies OR teen OR teens OR preteen OR preteens OR teenager OR teenagers OR pubescen* OR prepubescen* OR neonate OR neonates) | 3,331,552 |
| #3 | TS=(((sign OR signs OR symptom OR symptoms OR complaint OR complaints) OR ((clinical AND (manifestation OR manifestations OR feature OR features OR finding OR findings OR aspect OR aspects OR marker OR markers)))) OR (heartburn OR "difficulty swallowing" OR nausea OR "chronic cough" OR bloating OR "refusing to feed" OR regurgitation OR (("upper abdominal" OR chest) AND pain) OR "sore throat" OR wheezing OR dysphagia OR laryngitis OR vomiting OR "weight loss" OR pneumonia OR "dental erosion" OR "dental erosions" OR "otitis media" OR BRUE OR "Brief Resolved Unexplained Event" OR ALTE OR "Apparent Life-Threatening Event" OR apnea OR apnoea OR Sandifer)) | 3,792,936 |
| #4 | #1 AND #2 AND #3 | 5,759 |
| #5 | #1 AND #2 AND #3 AND English (Languages) AND Article or Review Article (Document Types) AND TS=(observational OR prospective OR retrospective OR follow-up study OR longitudinal OR cohort OR cross-sectional OR case-control) | 1,722 |

### PICO 3 - What are the risk factors for GERD in infants, children, and adolescents?

| PubMed, 11/05/2024 | | |
| --- | --- | --- |
| Search | Query | Results |
| #1 | gastroesophageal reflux OR gastro esophageal reflux OR gastrooesophageal reflux OR gastro oesophageal reflux OR GER OR GERD OR GOR OR GORD OR (gastric[Title/Abstract] AND (acid[Title/Abstract] OR reflux[Title/Abstract])) OR (reflux[Title/Abstract] AND (oesophagitis[Title/Abstract] OR esophagitis[Title/Abstract])) OR (erosive[Title/Abstract] AND (oesophag*[Title/Abstract] OR esophag*[Title/Abstract])) OR (regurgitation[Title/Abstract] NOT (aortic[Title/Abstract] OR mitral[Title/Abstract] OR tricuspid[Title/Abstract] OR valve[Title/Abstract] OR valvular[Title/Abstract] OR paravalvular[Title/Abstract] OR pulmonary [Title/Abstract])) OR "acid reflux"[Title/Abstract] | 95,570 |
| #2 | child OR children OR pediatric OR pediatrics OR paediatric OR paediatrics OR infant OR infants OR infancy OR newborn OR newborns OR adolescent OR adolescents OR juvenile OR youth OR toddler OR toddlers OR kid OR kids OR boy OR boys OR girl OR girls OR baby OR babies OR teen OR teens OR preteen OR preteens OR teenager OR teenagers OR pubescen* OR prepubescen* OR neonate OR neonates OR (allchild[Filter] OR newborn[Filter] OR allinfant[Filter] OR infant[Filter] OR child[Filter] OR adolescent[Filter] OR preschoolchild[Filter]) | 6,804,615 |
| #3 | risk factor OR risk factors OR risk assessment OR (risk[Title/Abstract] AND (assess*[Title/Abstract] OR factor[Title/Abstract] OR factors[Title/Abstract])) OR premature birth OR (pre-term[Title/Abstract] OR preterm[Title/Abstract] OR premature*[Title/Abstract]) OR developmental disabilities OR (neurodevelopmental[Title/Abstract] AND disorder*[Title/Abstract]) OR neurodisabilit*[Title/Abstract] OR brain injuries OR "Brain Damage, Chronic"[Mesh] OR ((cerebral[Title/Abstract] OR brain[Title/Abstract]) AND pals*[Title/Abstract]) OR (spastic[Title/Abstract] AND dipleg*[Title/Abstract]) OR "Nervous System Diseases"[Mesh] OR "Disabled Children"[Mesh] | 5,347,583 |
| #4 | "Esophagitis"[Mesh] | 13,800 |
| #5 | "Family"[Mesh] OR "Family Health"[Mesh] OR "Genetic Predisposition to Disease"[Mesh] OR ((family[Title/Abstract] OR familial[Title/Abstract]) AND history[Title/Abstract]) | 641,020 |
| #6 | #4 AND #5 | 143 |
| #7 | "Esophagitis/genetics"[Mesh] | 274 |
| #8 | #6 OR #7 | 366 |
| #9 | "Hernia, Diaphragmatic"[Mesh] OR ((diaphrag*[Title/Abstract] OR bochdalek[Title/Abstract] OR morgagni[Title/Abstract] OR hiat*[Title/Abstract]) AND hernia*[Title/Abstract]) OR "Esophageal Atresia"[Mesh] OR ((esophag*[Title/Abstract] OR oesophag*[Title/Abstract]) AND atresia[Title/Abstract]) OR "Age Factors"[Mesh] OR "Age of Onset"[Mesh] OR (age[Title/Abstract] AND onset[Title/Abstract]) OR "Obesity"[Mesh] OR "Obesity, Morbid"[Mesh] OR "Body Weight"[Mesh] OR "Overweight"[Mesh] OR ((obese[Title/Abstract] OR obesity[Title/Abstract] OR over[Title/Abstract]) AND weight[Title/Abstract]) OR "Alcohol Drinking"[Mesh] OR alcohol*[Title/Abstract] OR "Smoking"[Mesh] OR (smok*[Title/Abstract] OR cigar*[Title/Abstract] OR tabacco[Title/Abstract]) OR "chronic lung disease of infancy"[Title/Abstract] OR ((bronchopulmonary[Title/Abstract] OR lung*[Title/Abstract]) AND dysplasia*[Title/Abstract]) OR (chronic[Title/Abstract] AND (lung[Title/Abstract] OR pulmonary[Title/Abstract] OR pneumopathy[Title/Abstract])) | 2,137,795 |
| #10 | ("Chronic Disease"[Mesh] AND "Lung"[Mesh]) | 17,684 |
| #11 | "Heart Defects, Congenital"[Mesh] | 172,611 |
| #12 | (congenital[Title/Abstract] AND (heart[Title/Abstract] OR cardiac[Title/Abstract]) AND (disease*[Title/Abstract] OR distress*[Title/Abstract] OR failure[Title/Abstract])) | 52,591 |
| #13 | #10 OR #11 OR #12 | 212,235 |
| #14 | #1 AND #2 AND (#3 OR #8 OR #9 OR #13) | 8,191 |
| #15 | #14 AND english[Filter] AND humans[Filter] AND (clinicalstudy[Filter] OR comparativestudy[Filter] OR meta-analysis[Filter] OR observationalstudy[Filter] OR systematicreview[Filter]) | 1,169 |

| Embase, 11/05/2024 | | |
| --- | --- | --- |
| Search | Query | Results |
| #1 | 'gastroesophageal reflux'/exp OR 'gastroesophageal reflux':ti,ab,kw OR 'gastro esophageal reflux':ti,ab,kw OR 'gastrooesophageal reflux':ti,ab,kw OR 'gastro oesophageal reflux':ti,ab,kw OR ger:ti,ab,kw OR gerd:ti,ab,kw OR gor:ti,ab,kw OR gord:ti,ab,kw OR (gastric:ti,ab,kw AND (acid:ti,ab,kw OR reflux:ti,ab,kw)) OR (reflux:ti,ab,kw AND (oesophagitis:ti,ab,kw OR esophagitis:ti,ab,kw)) OR (erosive:ti,ab,kw AND (oesophag*:ti,ab,kw OR esophag*:ti,ab,kw)) OR (regurgitation:ti,ab,kw NOT (aortic:ti,ab,kw OR mitral:ti,ab,kw OR tricuspid:ti,ab,kw OR valve:ti,ab,kw OR valvular:ti,ab,kw OR paravalvular:ti,ab,kw OR pulmonary:ti,ab,kw)) OR 'acid reflux':ti,ab,kw | 143,723 |
| #2 | child:ti,ab,kw OR children:ti,ab,kw OR pediatric:ti,ab,kw OR pediatrics:ti,ab,kw OR paediatric:ti,ab,kw OR paediatrics:ti,ab,kw OR infant:ti,ab,kw OR infants:ti,ab,kw OR infancy:ti,ab,kw OR newborn:ti,ab,kw OR newborns:ti,ab,kw OR adolescent:ti,ab,kw OR adolescents:ti,ab,kw OR juvenile:ti,ab,kw OR youth:ti,ab,kw OR toddler:ti,ab,kw OR toddlers:ti,ab,kw OR kid:ti,ab,kw OR kids:ti,ab,kw OR boy:ti,ab,kw OR boys:ti,ab,kw OR girl:ti,ab,kw OR girls:ti,ab,kw OR baby:ti,ab,kw OR babies:ti,ab,kw OR teen:ti,ab,kw OR teens:ti,ab,kw OR preteen:ti,ab,kw OR preteens:ti,ab,kw OR teenager:ti,ab,kw OR teenagers:ti,ab,kw OR pubescen*:ti,ab,kw OR prepubescen*:ti,ab,kw OR neonate:ti,ab,kw OR neonates:ti,ab,kw | 3,532,866 |
| #3 | 'risk factor' OR 'risk factors' OR 'risk assessment' OR (risk:ti,ab,kw AND (assess*:ti,ab,kw OR factor:ti,ab,kw OR factors:ti,ab,kw)) OR 'premature birth' OR (pre-term:ti,ab,kw OR preterm:ti,ab,kw OR premature*:ti,ab,kw) OR 'developmental disabilities' OR (neurodevelopmental:ti,ab,kw AND disorder*:ti,ab,kw) OR neurodisabilit*:ti,ab,kw OR 'brain injuries' OR 'chronic brain disease'/exp OR ((cerebral:ti,ab,kw OR brain:ti,ab,kw) AND pals*:ti,ab,kw) OR (spastic:ti,ab,kw AND dipleg*:ti,ab,kw) OR 'neurologic disease'/exp OR 'disabled child'/exp | 4,907,362 |
| #4 | 'esophagitis'/exp | 46,686 |
| #5 | 'family'/exp OR 'family health'/exp OR 'genetic predisposition'/exp OR ((family:ti,ab,kw OR familial:ti,ab,kw) AND history:ti,ab,kw) | 1,005,504 |
| #6 | #4 AND #5 | 913 |
| #7 | 'esophagitis'/exp AND 'genetics'/exp | 294 |
| #8 | #6 OR #7 | 1,174 |
| #9 | 'diaphragm hernia'/exp OR ((diaphrag*:ti,ab,kw OR bochdalek:ti,ab,kw OR morgagni:ti,ab,kw OR hiat*:ti,ab,kw) AND hernia*:ti,ab,kw) OR 'esophagus atresia'/exp OR ((esophag*:ti,ab,kw OR oesophag*:ti,ab,kw) AND atresia:ti,ab,kw) OR 'age factors' OR 'onset age'/exp OR (age:ti,ab,kw AND onset:ti,ab,kw) OR 'obesity'/exp OR 'morbid obesity'/exp OR 'body weight'/exp OR overweight OR ((obese:ti,ab,kw OR obesity:ti,ab,kw OR over:ti,ab,kw) AND weight:ti,ab,kw) OR 'drinking behavior'/exp OR alcohol*:ti,ab,kw OR 'smoking'/exp OR (smok*:ti,ab,kw OR cigar*:ti,ab,kw OR tabacco:ti,ab,kw) OR "chronic lung disease of infancy":ti,ab,kw OR ((bronchopulmonary:ti,ab,kw OR lung*:ti,ab,kw) AND dysplasia*:ti,ab,kw) OR (chronic:ti,ab,kw AND (lung:ti,ab,kw OR pulmonary:ti,ab,kw OR pneumopathy:ti,ab,kw)) | 3,127,444 |
| #10 | 'chronic disease'/exp AND 'lung'/exp | 2,968 |
| #11 | 'congenital heart malformation'/exp | 182,020 |
| #12 | (congenital:ti,ab,kw AND (heart:ti,ab,kw OR cardiac:ti,ab,kw) AND (disease*:ti,ab,kw OR distress*:ti,ab,kw OR failure:ti,ab,kw)) | 83,446 |
| #13 | #10 OR #11 OR #12 | 231,879 |
| #14 | #1 AND #2 AND (#3 OR #8 OR #9 OR #13) | 9,194 |
| #15 | #14 AND [english]/lim AND [humans]/lim AND ([article]/lim OR [article in press]/lim OR [review]/lim) AND ('cohort analysis'/de OR 'comparative study'/de OR 'cross sectional study'/de OR 'longitudinal study'/de OR 'meta analysis'/de OR 'observational study'/de OR 'prospective study'/de OR 'retrospective study'/de OR 'systematic review'/de) | 1,712 |

|  | Web of Science, 11/05/2024 |  |
| --- | --- | --- |
| Search | Query | Results |
| #1 | TS=(gastroesophageal reflux OR gastro esophageal reflux OR gastrooesophageal reflux OR gastro oesophageal reflux OR GER OR GERD OR GOR OR GORD OR (gastric AND (acid OR reflux)) OR (reflux AND (oesophagitis OR esophagitis)) OR (erosive AND (oesophag* OR esophag*)) OR (regurgitation NOT (aortic OR mitral OR tricuspid OR valve OR valvular OR paravalvular OR pulmonary )) OR "acid reflux") | 97,816 |
| #2 | TS=(child OR children OR pediatric OR pediatrics OR paediatric OR paediatrics OR infant OR infants OR infancy OR newborn OR newborns OR adolescent OR adolescents OR juvenile OR youth OR toddler OR toddlers OR kid OR kids OR boy OR boys OR girl OR girls OR baby OR babies OR teen OR teens OR preteen OR preteens OR teenager OR teenagers OR pubescen* OR prepubescen* OR neonate OR neonates) | 3,331,552 |
| #3 | TS=(risk factor OR risk factors OR risk assessment OR (risk AND (assess* OR factor OR factors)) OR premature birth OR (pre-term OR preterm OR premature*) OR developmental disabilities OR (neurodevelopmental AND disorder*) OR neurodisabilit* OR brain injuries OR chronic brain damage OR ((cerebral OR brain) AND pals*) OR (spastic AND dipleg*) OR nervous system diseases OR disabled children) | 2,998,107 |
| #4 | TS=(esophagitis) | 20,290 |
| #5 | TS=(family OR genetic predisposition OR ((family OR familial) AND history)) | 1,846,981 |
| #6 | #4 AND #5 | 333 |
| #7 | TS=(esophagitis AND genetics) | 64 |
| #8 | #6 OR #7 | 380 |
| #9 | TS=(diaphragmatic hernia OR ((diaphrag* OR bochdalek OR morgagni OR hiat*) AND hernia*) OR esophageal atresia OR ((esophag* OR oesophag*) AND atresia) OR age factors OR "age of onset" OR (age AND onset) OR obesity OR morbid obesity OR body weight OR overweight OR ((obese OR obesity OR over) AND weight) OR alcohol drinking OR alcohol* OR smoking OR smok* OR cigar* OR tabacco OR "chronic lung disease of infancy" OR ((bronchopulmonary OR lung*) AND dysplasia*) OR (chronic AND (lung OR pulmonary OR pneumopathy))) | 3,193,081 |
| #10 | TS=(chronic disease AND lung) | 67,792 |
| #11 | TS=(congenital heart defects) | 24,754 |
| #12 | TS=((congenital AND (heart OR cardiac) AND (disease* OR distress* OR failure))) | 57,435 |
| #13 | #10 OR #11 OR #12 | 132,011 |
| #14 | #1 AND #2 AND (#3 OR #8 OR #9 OR #13) | 3,949 |
| #15 | #14 AND English (Languages) AND Article or Review Article (Document Types) AND TS=(observational OR prospective OR retrospective OR follow-up study OR longitudinal OR cohort OR cross-sectional OR case-control) | 1,320 |

### PICO 4 – What is the value of different diagnostic tests for GERD in infants, children, and adolescents?

|  | PubMed, 11/05/2024 |  |
| --- | --- | --- |
| Search | Query | Results |
| #1 | gastroesophageal reflux OR gastro esophageal reflux OR gastrooesophageal reflux OR gastro oesophageal reflux OR GER OR GERD OR GOR OR GORD OR (gastric[Title/Abstract] AND (acid[Title/Abstract] OR reflux[Title/Abstract])) OR (reflux[Title/Abstract] AND (oesophagitis[Title/Abstract] OR esophagitis[Title/Abstract])) OR (erosive[Title/Abstract] AND (oesophag*[Title/Abstract] OR esophag*[Title/Abstract])) OR (regurgitation[Title/Abstract] NOT (aortic[Title/Abstract] OR mitral[Title/Abstract] OR tricuspid[Title/Abstract] OR valve[Title/Abstract] OR valvular[Title/Abstract] OR paravalvular[Title/Abstract] OR pulmonary [Title/Abstract])) OR "acid reflux"[Title/Abstract] | 95,570 |
| #2 | child OR children OR pediatric OR pediatrics OR paediatric OR paediatrics OR infant OR infants OR infancy OR newborn OR newborns OR adolescent OR adolescents OR juvenile OR youth OR toddler OR toddlers OR kid OR kids OR boy OR boys OR girl OR girls OR baby OR babies OR teen OR teens OR preteen OR preteens OR teenager OR teenagers OR pubescen* OR prepubescen* OR neonate OR neonates OR (allchild[Filter] OR newborn[Filter] OR allinfant[Filter] OR infant[Filter] OR child[Filter] OR adolescent[Filter] OR preschoolchild[Filter]) | 6,804,615 |
| #3 | "Diagnostic Techniques, Digestive System"[Mesh] OR ("Gastroesophageal Reflux/diagnosis"[Mesh] OR "Gastroesophageal Reflux/diagnostic imaging"[Mesh]) OR "Esophageal ph Monitoring"[Mesh] OR "barium swallow"[Title/Abstract] OR "barium esophagram"[Title/Abstract] OR "Esophagoscopy"[Mesh] OR "Gastroscopy"[Mesh] OR "Biopsy"[Mesh] OR "Electric Impedance"[Mesh] OR "Ultrasonography"[Mesh] OR "Manometry"[Mesh] OR "Radionuclide Imaging"[Mesh] OR "Endoscopy, Digestive System"[Mesh] OR "Endoscopy, Gastrointestinal"[Mesh] OR (barium[Title/Abstract] OR oesophagogastroduodenoscop*[Title/Abstract] OR esophagogastroduodenoscop*[Title/Abstract] OR esophagogastroscop*[Title/Abstract] OR oesophagogastroscop*[Title/Abstract] OR oesophago-gastroduodenoscop*[Title/Abstract] OR esophago-gastroduodenoscop*[Title/Abstract] OR esophago-gastroscop*[Title/Abstract] OR oesophago-gastroscop*[Title/Abstract] OR oesophago-gastro-duodenoscop*[Title/Abstract] OR esophago-gastro-duodenoscop*[Title/Abstract] OR gastroscop*[Title/Abstract] OR esophagoscop*[Title/Abstract] OR oesophagoscop*[Title/Abstract] OR biops*[Title/Abstract] OR pH-metry[Title/Abstract] OR pH-MII[Title/Abstract] OR impedance[Title/Abstract] OR manometr*[Title/Abstract] OR motility[Title/Abstract] OR HRM[Title/Abstract] OR HRIM[Title/Abstract] OR scintigraph*[Title/Abstract] OR ultraso*[Title/Abstract]) OR ((oesophag*[Title/Abstract] OR esophagi*[Title/Abstract] OR PH[Title/Abstract]) AND (monitoring*[Title/Abstract] OR recording*[Title/Abstract] OR measure*[Title/Abstract])) OR "Biomarkers"[Mesh] OR (biomarker*[Title/Abstract] OR biological marker*[Title/Abstract] OR pyloric feed*[Title/Abstract] OR pyloric food*[Title/Abstract]) OR "Saliva"[Mesh] OR "Sputum"[Mesh] OR (saliva*[Title/Abstract] OR sputum[Title/Abstract]) OR ((esophag*[Title/Abstract] OR oesophag*[Title/Abstract] OR ear[Title/Abstract] OR gastr*[Title/Abstract]) AND (fluid*[Title/Abstract])) | 3,263,689 |
| #4 | "Sensitivity and Specificity"[Mesh] OR "Diagnosis"[Mesh] OR "Diagnosis, Differential"[Mesh] OR "Diagnostic Techniques, Digestive System"[Mesh] OR "Gastric Acidity Determination"[Mesh] OR "Gastroesophageal Reflux/diagnosis"[Mesh] OR "Gastrointestinal Motility"[Mesh] OR "Reproducibility of Results"[Mesh] OR (sensitiv*[Title/Abstract] OR specific*[Title/Abstract] OR value*[Title/Abstract] OR diagnos*[Title/Abstract] OR accura*[Title/Abstract]) | 16,241,954 |
| #5 | #1 AND #2 AND #3 AND #4 | 7,861 |
| #6 | #5 AND ((clinicalstudy[Filter] OR clinicaltrial[Filter] OR comparativestudy[Filter] OR controlledclinicaltrial[Filter] OR meta-analysis[Filter] OR observationalstudy[Filter] OR randomizedcontrolledtrial[Filter] OR systematicreview[Filter]) AND (humans[Filter]) AND english[Filter]) | 1,390 |

| Embase, 11/05/2024 | | |
| --- | --- | --- |
| Search | Query | Results |
| #1 | 'gastroesophageal reflux'/exp OR 'gastroesophageal reflux':ti,ab,kw OR 'gastro esophageal reflux':ti,ab,kw OR 'gastrooesophageal reflux':ti,ab,kw OR 'gastro oesophageal reflux':ti,ab,kw OR ger:ti,ab,kw OR gerd:ti,ab,kw OR gor:ti,ab,kw OR gord:ti,ab,kw OR (gastric:ti,ab,kw AND (acid:ti,ab,kw OR reflux:ti,ab,kw)) OR (reflux:ti,ab,kw AND (oesophagitis:ti,ab,kw OR esophagitis:ti,ab,kw)) OR (erosive:ti,ab,kw AND (oesophag*:ti,ab,kw OR esophag*:ti,ab,kw)) OR (regurgitation:ti,ab,kw NOT (aortic:ti,ab,kw OR mitral:ti,ab,kw OR tricuspid:ti,ab,kw OR valve:ti,ab,kw OR valvular:ti,ab,kw OR paravalvular:ti,ab,kw OR pulmonary:ti,ab,kw)) OR 'acid reflux':ti,ab,kw | 143,723 |
| #2 | child:ti,ab,kw OR children:ti,ab,kw OR pediatric:ti,ab,kw OR pediatrics:ti,ab,kw OR paediatric:ti,ab,kw OR paediatrics:ti,ab,kw OR infant:ti,ab,kw OR infants:ti,ab,kw OR infancy:ti,ab,kw OR newborn:ti,ab,kw OR newborns:ti,ab,kw OR adolescent:ti,ab,kw OR adolescents:ti,ab,kw OR juvenile:ti,ab,kw OR youth:ti,ab,kw OR toddler:ti,ab,kw OR toddlers:ti,ab,kw OR kid:ti,ab,kw OR kids:ti,ab,kw OR boy:ti,ab,kw OR boys:ti,ab,kw OR girl:ti,ab,kw OR girls:ti,ab,kw OR baby:ti,ab,kw OR babies:ti,ab,kw OR teen:ti,ab,kw OR teens:ti,ab,kw OR preteen:ti,ab,kw OR preteens:ti,ab,kw OR teenager:ti,ab,kw OR teenagers:ti,ab,kw OR pubescen*:ti,ab,kw OR prepubescen*:ti,ab,kw OR neonate:ti,ab,kw OR neonates:ti,ab,kw | 3,532,866 |
| #3 | 'digestive system examination'/exp OR 'esophageal pH monitoring'/exp OR 'barium swallow':ti,ab,kw OR 'barium esophagram':ti,ab,kw OR 'esophagoscopy'/exp OR 'gastroscopy'/exp OR 'biopsy'/exp OR 'impedance'/exp OR 'ultrasonography'/exp OR 'manometry'/exp OR 'scintiscanning'/exp OR 'digestive tract endoscopy'/exp OR 'gastrointestinal endoscopy'/exp OR (barium:ti,ab,kw OR oesophagogastroduodenoscop*:ti,ab,kw OR esophagogastroduodenoscop*:ti,ab,kw OR esophagogastroscop*:ti,ab,kw OR oesophagogastroscop*:ti,ab,kw OR oesophago-gastroduodenoscop*:ti,ab,kw OR esophago-gastroduodenoscop*:ti,ab,kw OR esophago-gastroscop*:ti,ab,kw OR oesophago-gastroscop*:ti,ab,kw OR oesophago-gastro-duodenoscop*:ti,ab,kw OR esophago-gastro-duodenoscop*:ti,ab,kw OR gastroscop*:ti,ab,kw OR esophagoscop*:ti,ab,kw OR oesophagoscop*:ti,ab,kw OR biops*:ti,ab,kw OR pH-metry:ti,ab,kw OR pH-MII:ti,ab,kw OR impedance:ti,ab,kw OR manometr*:ti,ab,kw OR motility:ti,ab,kw OR HRM:ti,ab,kw OR HRIM:ti,ab,kw OR scintigraph*:ti,ab,kw OR ultraso*:ti,ab,kw) OR ((oesophag*:ti,ab,kw OR esophagi*:ti,ab,kw OR PH:ti,ab,kw) AND (monitoring*:ti,ab,kw OR recording*:ti,ab,kw OR measure*:ti,ab,kw)) OR 'biological marker'/exp OR (biomarker*:ti,ab,kw OR biological marker*:ti,ab,kw OR pyloric feed*:ti,ab,kw OR pyloric food*:ti,ab,kw) OR 'saliva'/exp OR 'sputum'/exp OR (saliva*:ti,ab,kw OR sputum:ti,ab,kw) OR ((esophag*:ti,ab,kw OR oesophag*:ti,ab,kw OR ear:ti,ab,kw OR gastr*:ti,ab,kw) AND (fluid*:ti,ab,kw)) | 4,041,747 |
| #4 | 'sensitivity and specificity'/exp OR 'diagnosis'/exp OR 'differential diagnosis'/exp OR 'stomach pH'/exp OR 'gastrointestinal motility'/exp OR 'reproducibility'/exp OR (sensitiv*:ti,ab,kw OR specific*:ti,ab,kw OR value*:ti,ab,kw OR diagnos*:ti,ab,kw OR accura*:ti,ab,kw) | 17,660,415 |
| #5 | #1 AND #2 AND #3 AND #4 | 6,305 |
| #6 | #5 AND ([article]/lim OR [article in press]/lim OR [review]/lim) AND [english]/lim AND [humans]/lim AND ('clinical article'/de OR 'clinical trial'/de OR 'comparative study'/de OR 'cross sectional study'/de OR 'diagnostic test accuracy study'/de OR 'longitudinal study'/de OR 'meta analysis'/de OR 'observational study'/de OR 'randomized controlled trial'/de OR 'systematic review'/de) | 1,570 |

| Web of Science, 11/05/2024 | | |
| --- | --- | --- |
| Search | Query | Results |
| #1 | TS=(gastroesophageal reflux OR gastro esophageal reflux OR gastrooesophageal reflux OR gastro oesophageal reflux OR GER OR GERD OR GOR OR GORD OR (gastric AND (acid OR reflux)) OR (reflux AND (oesophagitis OR esophagitis)) OR (erosive AND (oesophag* OR esophag*)) OR (regurgitation NOT (aortic OR mitral OR tricuspid OR valve OR valvular OR paravalvular OR pulmonary )) OR "acid reflux") | 97,816 |
| #2 | TS=(child OR children OR pediatric OR pediatrics OR paediatric OR paediatrics OR infant OR infants OR infancy OR newborn OR newborns OR adolescent OR adolescents OR juvenile OR youth OR toddler OR toddlers OR kid OR kids OR boy OR boys OR girl OR girls OR baby OR babies OR teen OR teens OR preteen OR preteens OR teenager OR teenagers OR pubescen* OR prepubescen* OR neonate OR neonates) | 3,331,552 |
| #3 | TS=(diagnostic techniques digestive system OR ((gastroesophageal reflux AND diagnosis) OR (gastroesophageal reflux AND diagnostic imaging)) OR esophageal ph monitoring OR "barium swallow" OR "barium esophagram" OR esophagoscopy OR gastroscopy OR biopsy OR electric Impedance OR ultrasonography OR manometry OR radionuclide imaging OR endoscopy digestive system OR endoscopy gastrointestinal OR (barium OR oesophagogastroduodenoscop* OR esophagogastroduodenoscop* OR esophagogastroscop* OR oesophagogastroscop* OR oesophago-gastroduodenoscop* OR esophago-gastroduodenoscop* OR esophago-gastroscop* OR oesophago-gastroscop* OR oesophago-gastro-duodenoscop* OR esophago-gastro-duodenoscop* OR gastroscop* OR esophagoscop* OR oesophagoscop* OR biops* OR pH-metry OR pH-MII OR impedance OR manometr* OR motility OR HRM OR HRIM OR scintigraph* OR ultraso*) OR ((oesophag* OR esophagi* OR PH) AND (monitoring* OR recording* OR measure*)) OR biomarkers OR (biomarker* OR biological marker* OR pyloric feed* OR pyloric food*) OR saliva OR sputum OR ((esophag* OR oesophag* OR ear OR gastr*) AND (fluid*))) | 2,605,503 |
| #4 | TS=(gastric acidity determination OR gastrointestinal motility OR reproducibility OR sensitiv* OR specific* OR value* OR diagnos* OR accura*) | 16,996,672 |
| #5 | #1 AND #2 AND #3 AND #4 | 3,192 |
| #6 | #5 AND Article or Review Article (Document Types) AND English (Languages) AND TS=(observational OR prospective OR retrospective OR longitudinal OR cohort OR cross-sectional OR case-control OR systematic review OR meta-analysis OR prevalence study) | 1,076 |

## PRISMA Flow diagrams

### Search strategy to identify guidelines and consensus statements on gastroesophageal reflux (GER) and gastroesophageal reflux disease (GERD) in infants, children, and adolescents

Records identified from:

Databases (n = 300)

- PubMed (n = 97)
- Embase (n = 110)
- Web of Science (n = 93)

Google Scholar (n = 50)

Records removed *before screening*:

Duplicate records removed (n = 133)

Records screened

(n = 217)

Records excluded

(n = 187)

Reports sought for retrieval

(n = 30)

Reports not retrieved

(n = 0)

Reports assessed for eligibility

(n = 30)

Reports excluded (n = 27):

- Wrong population (n = 2)
- Wrong study design (n= 22)
- Duplicates (n = 3)

Studies included in review

(n = 3)

Reports of included studies

(n = 3)

**Identification of studies via databases and registers**

**Identification**

**Screening**

**Included**

### PICO 1 – What is the definition of gastroesophageal reflux (GER) and gastroesophageal reflux disease (GERD) in infants, children, and adolescents?


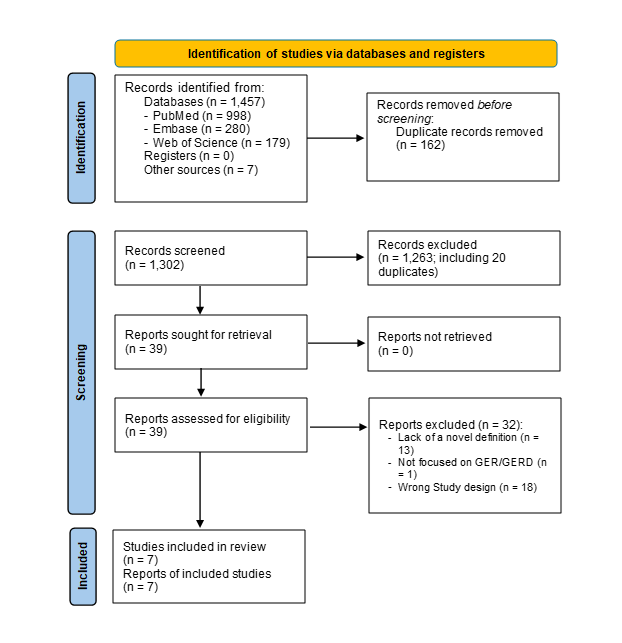


### PICO 2– What are the signs and symptoms indicative of GERD in infants, children, and adolescents?

Records identified from:

Databases (n = 4,717)

- PubMed (n = 1,349)
- Embase (n = 1,646)
- Web of Science (n = 1,722)

Registers (n = 0)

Records removed *before screening*:

Duplicate records removed (n = 846)

Records screened

(n = 3,871)

Records excluded

(n = 3,750; including 244 duplicates)

Reports sought for retrieval

(n = 121)

Reports not retrieved

(n = 4)

Reports assessed for eligibility

(n = 117)

Reports excluded: (n = 58):

- Population (n = 33)
- Outcome (n = 4)
- Study design (n = 4)
- Study objective (n = 15)
- Language (n = 1)
- Duplicate reports (n = 1)

Studies included in review

(n = 59)

Reports of included studies

(n = 59)

**Identification of studies via databases and registers**

**Identification**

**Screening**

**Included**

### PICO 3 - What are the risk factors for GERD in infants, children, and adolescents?

Records identified from:

Databases (n = 4,201)

- PubMed (n = 1,169)
- Embase (n = 1,712)
- Web of Science (n = 1,320)

Registers (n = 0)

Records removed *before screening*:

Duplicate records removed (n = 598)

Records screened

(n = 3,603)

Records excluded

(n = 3,558; including 199 duplicates)

Reports sought for retrieval

(n = 45)

Reports not retrieved

(n = 0)

Reports assessed for eligibility

(n = 45)

Reports excluded (n = 27):

- Missing data (n = 3)
- Wrong study design (n = 5)
- Wrong population (n = 7)
- Wrong comparator (n = 6)
- Wrong outcome (n = 6)

Studies included in review

(n = 18)

Reports of included studies

(n = 18)

**Identification of studies via databases and registers**

**Identification**

**Screening**

**Included**

### PICO 4 – What is the value of different diagnostic tests for GERD in infants, children, and adolescents?

Records identified from:

Databases (n = 4,036)

- PubMed (n = 1,390)
- Embase (n = 1,570)
- Web of Science (n = 1,076)

Registers (n = 0)

Records removed *before screening*:

Duplicate records removed (n = 388)

Records screened

(n = 3,648)

Total records excluded (n = 3,549; including 129 duplicates)

Reports sought for retrieval

(n = 99)

Reports not retrieved

(n = 1)

Reports assessed for eligibility

(n = 98)

Reports excluded (n = 59)

- Wrong study design (n = 4)
- Wrong population (n = 6)
- Wrong intervention (n = 1)
- Wrong comparator (n = 12)
- Wrong outcome (n = 34)
- Duplicate report (n = 1)
- Not in English (n = 1)

Studies included in review

(n = 39)

Reports of included studies

(n = 39)

**Identification of studies via databases and registers**

**Identification**

**Screening**

**Included**

## Excluded studies

### Search strategy to identify guidelines and consensus statements on gastroesophageal reflux (GER) and gastroesophageal reflux disease (GERD) in infants, children, and adolescents

| Study | Reason for exclusion |
| --- | --- |
| (2003). "IPEG guidelines for surgical treatment of pediatric gastroesophageal reflux disease (GERD)." Pediatric Endosurgery and Innovative Techniques 7(2): 210-213. | Wrong study design |
| (2008). "IPEG guidelines for the surgical treatment of pediatric gastroesophageal reflux disease (GERD)." J Laparoendosc Adv Surg Tech A 18(6): x-xiii. | Wrong study design |
| Ayerbe, J. I. G., et al. (2019). "Diagnosis and Management of Gastroesophageal Reflux Disease in Infants and Children: from Guidelines to Clinical Practice." Pediatric Gastroenterology Hepatology & Nutrition 22(2): 107-121. | Wrong study design |
| Barfield, E. and M. W. Parker (2019). "Management of Pediatric Gastroesophageal Reflux Disease." JAMA Pediatr 173(5): 485-486. | Wrong study design |
| Butler, N. (2009). "National Guidelines at a glance: GORD." SA Pharmaceutical Journal 76(9): 32-36. | Wrong study design |
| Chang, A. B., et al. (2019). "Chronic Cough and Gastroesophageal Reflux in Children: CHEST Guideline and Expert Panel Report." Chest 156(1): 131-140. | Wrong population |
| Davies, I., et al. (2015). "Gastro-oesophageal reflux disease in children: NICE guidance." Bmj 350: g7703. | Wrong study design |
| Daza, W., et al. (2017). "Methodological quality of clinical practice guidelines to management and/or treatment gastroesophageal reflux disease in paediatrics." J Pediatr Gastroenterol Nutr 64: 362. | Wrong study design |
| Fuchs, K. H., et al. (2014). "EAES recommendations for the management of gastroesophageal reflux disease." Surg Endosc 28(6): 1753-1773. | Wrong population |
| Gold, B. and P. Sherman (2008). "A global, evidence-based consensus on the definition of pediatric gastroesophageal reflux disease (GERD)." American Journal of Gastroenterology 103: S36-S36. | Wrong study design |
| Kane, T. D., et al. (2009). "Position paper on laparoscopic antireflux operations in infants and children for gastroesophageal reflux disease. American Pediatric Surgery Association." J Pediatr Surg 44(5): 1034-1040. | Wrong study design |
| Lightdale 2013. Gastroesophageal Reflux: Management Guidance for the Pediatrician. www.pediatrics.org/cgi/doi/10.1542/peds.2013-0421 doi:10.1542/peds.2013-0421 | Wrong study design |
| Lopez, R. N. and D. A. Lemberg (2020). "Gastro-oesophageal reflux disease in infancy: a review based on international guidelines." Med J Aust 212(1): 40-44. | Wrong study design |
| Mohan, N., et al. (2021). "Diagnosis and Management of Gastroesophageal Reflux Disease in Children: Recommendations of Pediatric Gastroenterology Chapter of Indian Academy of Pediatrics, Indian Society of Pediatric Gastroenterology, Hepatology and Nutrition (ISPGHAN)." Indian Pediatr 58(12): 1163-1170. | Wrong study design |
| Papachrisanthou, M. M. and R. L. Davis (2015). "Clinical Practice Guidelines for the Management of Gastroesophageal Reflux and Gastroesophageal Reflux Disease: Birth to 1 Year of Age." J Pediatr Health Care 29(6): 558-564. | Wrong study design |
| Papachrisanthou, M. M. and R. L. Davis (2016). "Clinical Practice Guidelines for the Management of Gastroesophageal Reflux and Gastroesophageal Reflux Disease: 1 Year to 18 Years of Age." J Pediatr Health Care 30(3): 289-294. | Wrong study design |
| Randel, A. (2014). "AAP releases guideline for the management of gastroesophageal reflux in children." Am Fam Physician 89(5): 395-397. | Wrong study design |
| Rerksuppaphol, S. and G. Barnes (2002). "Guidelines for evaluation and treatment of gastroesophageal reflux in infants and children: recommendations of the North American Society for Pediatric Gastroenterology and Nutrition." J Pediatr Gastroenterol Nutr 35(4): 583. | Wrong study design |
| Rodgers, A. (2021). "Gastro-oesophageal reflux in preterm infants: American Academy of Pediatrics guideline 2018." Arch Dis Child Educ Pract Ed 106(2): 107. | Wrong study design |
| Rudolph, C. D., et al. (2001). "Guidelines for evaluation and treatment of gastroesophageal reflux in infants and children: Recommendations of the North America Society for Pediatric Gastroenterology and Nutrition." J Pediatr Gastroenterol Nutr 32(SUPPL. 2): S1-S31. | Wrong study design |
| Sherman, P. (2009). "A global, evidence-based consensus on the definition of gastroesophageal reflux disease (GERD) in the pediatric population." Canadian Journal of Gastroenterology 23. | Wrong study design |
| Sherman, P. M., et al. (2009). "A Global, Evidence-Based Consensus on the Definition of Gastroesophageal Reflux Disease in the Pediatric Population." American Journal of Gastroenterology 104(5): 1278-1295. | Wrong study design |
| Vandenplas, Y., et al. (2023). "Infant gastroesophageal reflux disease management consensus." Acta Paediatrica. | Wrong study design |
| Vandenplas, Y., et al. (2009). "Pediatric gastroesophageal reflux clinical practice guidelines: Joint recommendations of the North American Society for Pediatric Gastroenterology, Hepatology, and Nutrition (NASPGHAN) and the European Society for Pediatric Gastroenterology, Hepatology, and Nutrition (ESPGHAN)." J Pediatr Gastroenterol Nutr 49(4): 498-547. | Wrong study design |
| (2009). "IPEG guidelines for the surgical treatment of pediatric gastroesophageal reflux disease (GERD)." J Laparoendosc Adv Surg Tech A 19 Suppl 1: x-xiii. | Duplicate |
| (2018). 2018 surveillance of gastro-oesophageal reflux disease in children and young people: diagnosis and management (NICE guideline NG1). London, National Institute for Health and Care Excellence (NICE). Copyright © NICE 2018. | duplicate |
| Gonzalez Ayerbe, J. I., et al. (2019). "Diagnosis and Management of Gastroesophageal Reflux Disease in Infants and Children: from Guidelines to Clinical Practice." Pediatr Gastroenterol Hepatol Nutr 22(2): 107-121. | Duplicate |

### PICO 1– What is the definition of gastroesophageal reflux (GER) and gastroesophageal reflux disease (GERD) in infants, children, and adolescents?

| Study | Reason for exclusion |
| --- | --- |
| Adamiak, T. and K. F. Plati (2018). "Pediatric Esophageal Disorders: Diagnosis and Treatment of Reflux and Eosinophilic Esophagitis." Pediatr Rev 39(8): 392-402. | Wrong study design |
| Aktaş, A., et al. (1999). "The relation between the degree of gastro-oesophageal reflux and the rate of gastric emptying." Nucl Med Commun 20(10): 907-910. | Lack of a novel definition |
| Al Saadi, T., et al. (2016). "Epidemiology and risk factors of uninvestigated dyspepsia, irritable bowel syndrome, and gastroesophageal reflux disease among students of Damascus University, Syria." J Epidemiol Glob Health 6(4): 285-293. | Wrong study design |
| Bardhan, K. D., et al. (2006). "Reflux rising! An essay on witnessing a disease in evolution." Dig Liver Dis 38(3): 163-168. | Lack of a novel definition |
| Bhatia, J. and A. Parish (2009). "GERD or not GERD: the fussy infant." J Perinatol 29 Suppl 2: S7-11. | Wrong study design |
| Chandrasoma, P. (2003). "Pathological basis of gastroesophageal reflux disease." World J Surg 27(9): 986-993. | Wrong study design |
| DeVault, K., et al. (2013). "Defining esophageal landmarks, gastroesophageal reflux disease, and Barrett's esophagus." Ann N Y Acad Sci 1300: 278-295. | Wrong study design |
| Diaz-Oliva, S. E., et al. (2020). "Oesophageal eosinophilia and oesophageal diseases in children: are the limits clear?" BMJ Paediatr Open 4(1): e000680. | Wrong study design |
| Eastburn, M. M., et al. (2007). "Defining the relationship between gastroesophageal reflux and cough: probabilities, possibilities and limitations." Cough 3: 4. | Wrong study design |
| Fedorak, R. N., et al. (2010). "Canadian Digestive Health Foundation Public Impact Series: Gastroesophageal reflux disease in Canada: Incidence, prevalence, and direct and indirect economic impact." Canadian Journal of Gastroenterology 24(7): 431-434. | Lack of a novel definition |
| Furuta, G. T., et al. (2007). "Eosinophilic esophagitis in children and adults: a systematic review and consensus recommendations for diagnosis and treatment." Gastroenterology 133(4): 1342-1363. | Not focused on GER/GERD |
| Gold, B. D. (2003). "Outcomes of pediatric gastroesophageal reflux disease:: In the first year of life, in childhood, and in adults... Oh, and should we really leave <i>Helicobacter pylori</i> alone?" J Pediatr Gastroenterol Nutr 37: S33-S39. | Wrong study design |
| Goudswaard, E., et al. (2018). "Epidemiology of gastroesophageal reflux disease in children: A systematic review." J Pediatr Gastroenterol Nutr 66: 426-427. | Lack of a novel definition |
| Harris, J., et al. (2022). "Clinical Practice Guidelines on Pediatric Gastroesophageal Reflux Disease: A Systematic Quality Appraisal of International Guidelines." Pediatric Gastroenterology Hepatology & Nutrition 25(2): 109-120. | Lack of a novel definition |
| He, J., et al. (2010). "A population-based survey of the epidemiology of symptom-defined gastroesophageal reflux disease: the Systematic Investigation of Gastrointestinal Diseases in China." BMC Gastroenterol 10: 94. | Lack of a novel definition |
| Henry, S. M. (2004). "Discerning differences: gastroesophageal reflux and gastroesophageal reflux disease in infants." Adv Neonatal Care 4(4): 235-247. | Wrong study design |
| Hirsch, S. and R. Rosen (2023). "The Real Relevance of Nonacid Reflux in Pediatric Patients." J Clin Gastroenterol 57(8): 754-759. | Wrong study design |
| Jolley, S. G., et al. (1981). "The significance of gastroesophageal reflux patterns in children." J Pediatr Surg 16(6): 859-865. | Lack of a novel definition |
| Katzka, D. A. (2014). "The complex relationship between eosinophilic esophagitis and gastroesophageal reflux disease." Dig Dis 32(1-2): 93-97. | Wrong study design |
| Koppen, I. J. N., et al. (2017). "The pediatric Rome IV criteria: what's new?" Expert Rev Gastroenterol Hepatol 11(3): 193-201. | Lack of a novel definition |
| Nikaki, K. and D. Sifrim (2022). "Pathophysiology of Pediatric Gastroesophageal Reflux Disease: Similarities and Differences With Adults." J Clin Gastroenterol 56(2): 99-113. | Wrong study design |
| Rameau, A. and A. Mudry (2020). "When did gastro-esophageal reflux become a disease? A historical perspective on GER(D) nomenclature." Int J Pediatr Otorhinolaryngol 137: 110214. | Wrong study design |
| Salem, S. B., et al. (2009). "The potential impact of contemporary developments in the management of patients with gastroesophageal reflux disease undergoing an initial gastroscopy." Can J Gastroenterol 23(2): 99-104. | Lack of a novel definition |
| Sherman, P. (2009). "A global, evidence-based consensus on the definition of gastroesophageal reflux disease (GERD) in the pediatric population." Canadian Journal of Gastroenterology 23. | Lack of a novel definition |
| Sifrim, D. (2004). "Acid, weakly acidic and non-acid gastro-oesophageal reflux: differences, prevalence and clinical relevance." Eur J Gastroenterol Hepatol 16(9): 823-830. | Wrong study design |
| Singendonk, M. M. J., et al. (2019). "Development of a Core Outcome Set for Infant Gastroesophageal Reflux Disease." J Pediatr Gastroenterol Nutr 68(5): 655-661. | Lack of a novel definition |
| Sondheimer, J. (2002). "Expanding the definition of GE reflux." J Pediatr Gastroenterol Nutr 34(5): 511-512. | Lack of a novel definition |
| Sondheimer, J. (2002). "Expanding the definition of GE reflux." J Pediatr Gastroenterol Nutr 34(5): 511-512. | Wrong study design |
| Vakil, N. (2010). "Disease definition, clinical manifestations, epidemiology and natural history of GERD." Best Pract Res Clin Gastroenterol 24(6): 759-764. | Wrong study design |
| Vandenplas, B. Hauser, An updated review on gastro-esophageal reflux in pediatrics, Expet Rev. Gastroenterol. Hepatol. 9 (12) (2015) 1511–1521, https:// doi.org/10.1586/17474124.2015.1093932 | Wrong study design |
| Wagner, A., et al. (2014). "Relationship of gastro-esophageal reflux (GER) characteristics and management patterns in nicu infants: Effects on feeding success and feeding failure." Gastroenterology 146(5): S-748. | Lack of a novel definition |
| Zalzal, G. H. and L. P. Tran (2000). "Pediatric gastroesophageal reflux and laryngopharyngeal reflux." Otolaryngol Clin North Am 33(1): 151-161. | Wrong study design |

### PICO 2– What are the signs and symptoms indicative of GER and GERD in infants, children, and adolescents?

| Study | Reason for exclusion |
| --- | --- |
| Aceves, S. S., et al. (2009). "A symptom scoring tool for identifying pediatric patients with eosinophilic esophagitis and correlating symptoms with inflammation." Annals of Allergy, Asthma and Immunology 103(5): 401-406. | Wrong population |
| Alatas, F. S., et al. (2023). "Association Between Pediatric Gastroesophageal Reflux Disease Symptom and Quality of Life Questionnaire Score, Endoscopy and Biopsy in Children with Clinical Gastroesophageal Reflux Disease: A Prospective Study." Pediatric Gastroenterology Hepatology & Nutrition 26(4): 173-180. | Wrong study objective |
| Alfaro, E. V., et al. (2008). "Oral implications in children with gastroesophageal reflux disease." Current Opinion in Pediatrics 20(5): 576-583. | Wrong study design |
| Al-Khawari, H. A., et al. (2002). "Diagnosis of gastro-oesophageal reflux in children. Comparison between oesophageal pH and barium examinations." Pediatr Radiol 32(11): 765-770. | Wrong study objective |
| Alonso-Bermejo, C., et al. (2022). "Functional gastrointestinal disorders frequency by Rome IV criteria." Anales de Pediatria 96(5): 441-447. | Wrong population |
| Bolier, E. A., et al. (2015). "Systematic review: questionnaires for assessment of gastroesophageal reflux disease." Dis Esophagus 28(2): 105-120. | Wrong study objective |
| Carr, M. M., et al. (2001). "Correlation of findings at direct laryngoscopy and bronchoscopy with gastroesophageal reflux disease in children -: <i>A prospective study</i>." Archives of Otolaryngology-Head & Neck Surgery 127(4): 369-374. | Wrong study objective |
| Chang, A. B., et al. (2019). "Chronic Cough and Gastroesophageal Reflux in Children: CHEST Guideline and Expert Panel Report." Chest 156(1): 131-140. | Wrong study objective |
| Chen, G., et al. (2021). "An epidemiological survey of gastroesophageal reflux disease at the digestive endoscopy center in Guangzhou." Eur Arch Otorhinolaryngol 278(12): 4901-4908. | Wrong population |
| Chitkara, D. K., et al. (2007). "Incidence of presentation of common functional gastrointestinal disorders in children from birth to 5 years: A cohort study." Clinical Gastroenterology and Hepatology 5(2): 186-191. | Wrong population |
| Cleveland, R. H., et al. (1983). "GASTROESOPHAGEAL REFLUX IN CHILDREN - RESULTS OF A STANDARDIZED FLUOROSCOPIC APPROACH." American Journal of Roentgenology 141(1): 53-56. | Wrong population |
| Condino, A. A., et al. (2006). "Evaluation of gastroesophageal reflux in pediatric patients with asthma using impedance-pH monitoring." Journal of Pediatrics 149(2): 216-219.e211. | Wrong population |
| Condino, A. A., et al. (2006). "Evaluation of infantile acid and nonacid gastroesophageal reflux using combined pH monitoring and impedance measurement." J Pediatr Gastroenterol Nutr 42(1): 16-21. | Wrong population |
| Curien-Chotard, M. and P. Jantchou (2020). "Natural history of gastroesophageal reflux in infancy: New data from a prospective cohort." BMC Pediatr 20(1). | Wrong population |
| de Benedictis, F. M. and A. Bush (2018). "Respiratory manifestations of gastro-oesophageal reflux in children." Arch Dis Child 103(3): 292-+. | Wrong study design |
| De Oliveira, P. A., et al. (2016). "Dental Erosion in Children with Gastroesophageal Reflux Disease." Pediatr Dent 38(3): 246-250. | Wrong population |
| de Veer, A. J., et al. (2008). "Symptoms of gastroesophageal reflux disease in severely mentally retarded people: a systematic review." BMC Gastroenterol 8: 23. | Wrong population |
| Deal, L., et al. (2005). "Age-specific questionnaires distinguish GERD symptom frequency and severity in infants and young children: Development and initial validation." J Pediatr Gastroenterol Nutr 41(2): 178-185. | Wrong population |
| Dellon, E. S., et al. (2009). "Clinical, Endoscopic, and Histologic Findings Distinguish Eosinophilic Esophagitis From Gastroesophageal Reflux Disease." Clinical Gastroenterology and Hepatology 7(12): 1305-1313. | Wrong population |
| Demir, A. M. (2022). "Quality of Life in Children and Adolescents with Gastroesophageal Reflux Disease." Turkish Journal of Pediatric Disease 16(3): 235-241. | Wrong population |
| Duncan, D. R., et al. (2021). "Overlapping Symptoms of Gastroesophageal Reflux and Aspiration Highlight the Limitations of Validated Questionnaires." J Pediatr Gastroenterol Nutr 72(3): 372-377. | Wrong study objective |
| El Mouzan, M. I., et al. (2001). "Pattern of gastroesophageal reflux in children." Saudi Medical Journal 22(5): 419-422. | Wrong population |
| Falah, S., et al. (2022). "Prevalence of Recurrent Hospital Admission in Children with Recurrent Wheezing in Babylon Province." Open Access Macedonian Journal of Medical Sciences 10(B): 168-172. | Wrong study objective |
| Farahmand, F., et al. (2013). "Gastroesophageal reflux disease and tooth erosion: A cross-sectional observational study." Gut Liver 7(3): 278-281. | Wrong population |
| Friesen, C. A., et al. (2016). "Prevalence of overlap syndromes and symptoms in pediatric functional dyspepsia." BMC Gastroenterol 16(1). | Wrong population |
| Friesen, L. R., et al. (2017). "Is histologic esophagitis associated with dental erosion: a cross-sectional observational study?" BMC Oral Health 17(1): 116. | Wrong study objective |
| Ganesh, M., et al. (2016). "Acid Rather Than Nonacid Reflux Burden Is a Predictor of Tooth Erosion." J Pediatr Gastroenterol Nutr 62(2): 309-313. | Wrong study objective |
| Ghezzi, M., et al. (2011). "Acid and weakly acid gastroesophageal refluxes and type of respiratory symptoms in children." Respir Med 105(7): 972-978. | Wrong population |
| Gunasekaran, T. S., et al. (2008). "Prevalence and associated features of gastroesophageal reflux symptoms in a Caucasian-predominant adolescent school population." Dig Dis Sci 53(9): 2373-2379. | Wrong population |
| Hegar, B., et al. (2004). "Investigation of regurgitation and other symptoms of gastroesophageal reflux in Indonesian infants." World J Gastroenterol 10(12): 1795-1797. | Wrong population |
| Heine, R. G., et al. (2006). "Clinical predictors of pathological gastro-oesophageal reflux in infants with persistent distress." J Paediatr Child Health 42(3): 134-139. | Wrong population |
| Imtiaz, A., et al. (2019). "PREVALENCE AND ETIOLOGY OF ASTHMA AND ITS ASSOCIATION WITH GASTRO -ESOPHAGEAL REFLEX DISEASE. A SYSTEMATIC REVIEW STUDY." Indo American Journal of Pharmaceutical Sciences 6(5): 10164-10170. | Wrong population |
| Juzaud, M., et al. (2019). "Correlation Between Clinical Signs and High-resolution Manometry Data in Children." J Pediatr Gastroenterol Nutr 68(5): 642-647. | Wrong study objective |
| Kabakus, N. and A. Kurt (2006). "Sandifer Syndrome: A continuing problem of misdiagnosis." Pediatrics International 48(6): 622-625. | Wrong population |
| Karkos, P. D., et al. (2004). "Pediatric middle ear infections and gastroesophageal reflux." Int J Pediatr Otorhinolaryngol 68(12): 1489-1492. | Wrong study design |
| Karkos, P. D., et al. (2009). "Reflux and sleeping disorders: a systematic review." J Laryngol Otol 123(4): 372-374. | Wrong study design |
| Kato, D., et al. (2024). "A systematic review of Sandifer syndrome in children with severe gastroesophageal reflux." Pediatr Surg Int 40(1): 91. | Wrong study objective |
| Kaveh, M., et al. (2021). "Non-Surgical causes of bilious vomiting in neonates admitted in a Tertiary center." Acta Med Iran 59(5): 308-311. | Wrong population |
| Lee, J. A., et al. (2020). "Impact of early gastroesophageal reflux disease on childhood otologic outcomes." Int J Pediatr Otorhinolaryngol 134. | Wrong population |
| Magistà, A. M., et al. (2007). "Multichannel intraluminal impedance to detect relationship between gastroesophageal reflux and apnoea of prematurity." Digestive and Liver Disease 39(3): 216-221. | Wrong population |
| Malaeb, D., et al. (2020). "Factors associated with wheezing among Lebanese children: Results of a cross-sectional study." Allergol Immunopathol (Madr) 48(6): 523-529. | Wrong outcome |
| Megale, S., et al. (2006). "Gastroesophageal reflux disease: Its importance in ear, nose, and throat practice." Int J Pediatr Otorhinolaryngol 70(1): 81-88. | Wrong population |
| Mikel, S. B., et al. (2008). "Assessment and management of infants with apparent life-threatening events in the paediatric emergency department." European Journal of Emergency Medicine 15(4): 203-208. | Duplicate |
| Nelson, S. P., et al. (2000). "Prevalence of symptoms of gastroesophageal reflux during childhood -: <i>A pediatric practice-based survey</i>." Arch Pediatr Adolesc Med 154(2): 150-154. | Wrong population |
| Nunn, J. H., et al. (2003). "Dental erosion -- changing prevalence? A review of British National childrens' surveys." International journal of paediatric dentistry / the British Paedodontic Society [and] the International Association of Dentistry for Children 13(2): 98-105. | Wrong outcome |
| Olleta, L., et al. (2013). "Laryngitis recurrent childhood: Evaluation with multichannel intraluminal impedance (MII)." Acta Gastroenterologica Latinoamericana 43(1): 9-11. | Not in English |
| Orenstein, S. R., et al. (1996). "Reflux symptoms in 100 normal infants: diagnostic validity of the infant gastroesophageal reflux questionnaire." Clin Pediatr (Phila) 35(12): 607-614. | Wrong population |
| Ozmen, S., et al. (2012). "Role of laryngoscopy in children with respiratory complaints and suspected reflux." Allergol Immunopathol (Madr) 40(4): 204-209. | Wrong study objective |
| Pace, F., et al. (2008). "Systematic review: gastro-oesophageal reflux disease and dental lesions." Aliment Pharmacol Ther 27(12): 1179-1186. | Wrong population |
| Rosen, R., et al. (2013). "The utility of endoscopy and multichannel intraluminal impedance testing in children with cough and wheezing." Pediatr Pulmonol. | Wrong study objective |
| Ruigómez, A., et al. (2010). "Gastroesophageal reflux disease in children and adolescents in primary care." Scand J Gastroenterol 45(2): 139-146. | Wrong population |
| Saad, K., et al. (2021). "Prevalence and associated risk factors of recurrent otitis media with effusion in children in Upper Egypt." World Journal of Otorhinolaryngology - Head and Neck Surgery 7(4): 280-284. | Wrong study objective |
| Santiago-Burruchaga, M., et al. (2008). "Assessment and management of infants with apparent life-threatening events in the paediatric emergency department." European Journal of Emergency Medicine 15(4): 203-208. | Wrong outcome |
| Sarath Kumar, K. S., et al. (2018). "Oral manifestations of gastroesophageal reflux disease in children: A preliminary observational study." J Indian Soc Pedod Prev Dent 36(2): 125-129. | Wrong population |
| Sidhwa, F., et al. (2017). "Diagnosis and Treatment of the Extraesophageal Manifestations of Gastroesophageal Reflux Disease." Ann Surg 265(1): 63-67. | Wrong population |
| Taniguchi, M. H. and R. S. Moyer (1994). "Assessment of risk factors for pneumonia in dysphagic children significance of videofluoroscopic swallowing evaluation." Dev Med Child Neurol 36(6): 495-502. | Wrong outcome |
| Wu, Z. H., et al. (2021). "The Relationship Between Otitis Media With Effusion and Gastroesophageal Reflux Disease: A Meta-analysis." Otol Neurotol 42(3): e245-e253. | Wrong population |
| Young, R. J., et al. (2007). "A retrospective, case-control pilot study of the natural history of pediatric gastroesophageal reflux." Dig Dis Sci 52(2): 457-462. | Wrong study objective |

### PICO 3 - What are the risk factors for GERD in infants, children, and adolescents?

| Study | Reason for exclusion |
| --- | --- |
| Akinola, E., et al. (2004). "Gastroesophageal reflux in infants &lt;32 weeks gestational age at birth:: Lack of relationship to chronic lung disease." Am J Perinatol 21(2): 57-62. | Wrong population |
| Araújo, T. D. B., et al. (2022). "Oral health-related quality of life in children and adolescents with cerebral palsy: paired cross-sectional study." European Archives of Paediatric Dentistry 23(3): 391-398. | Wrong outcome |
| Baker, S. S., et al. (2008). "Infantile gastroesophageal reflux in a hospital setting." BMC Pediatr 8. | Wrong population |
| Barriga-Rivera, A., et al. (2015). "Inter-reflux and bolus clearance times in non-pathologic pediatric patients: Data support computational models." Diseases of the Esophagus 28(2): 138-144. | Wrong population |
| Bayram, A. K., et al. (2016). "Misdiagnosis of gastroesophageal reflux disease as epileptic seizures in children." Brain and Development 38(3): 274-279. | Wrong population |
| Benden, C., et al. (2005). "High prevalence of gastroesophageal reflux in children after lung transplantation." Pediatr Pulmonol 40(1): 68-71. | Wrong comparator |
| Chawla, S., et al. (2006). "Gastroesophageal reflux disorder: a review for primary care providers." Clin Pediatr (Phila) 45(1): 7-13. | Wrong study design |
| Cohen, S. (1976). "The diagnosis and management of gastroesophageal reflux." Adv Intern Med 21: 47-75. | Wrong study design |
| Colletti, R. B. and C. Di Lorenzo (2003). "Overview of pediatric gastroesophageal reflux disease and proton pump inhibitor therapy." J Pediatr Gastroenterol Nutr 37 Suppl 1: S7-s11. | Wrong study design |
| Funderburk, A., et al. (2016). "Temporal association between reflux-like behaviors and gastroesophageal reflux in preterm and term infants." J Pediatr Gastroenterol Nutr 62(4): 556-561. | Wrong outcome |
| Groben, P. A., et al. (1987). "Gastroesophageal reflux and esophagitis in infants and children." Perspect Pediatr Pathol 11: 124-151. | Wrong study design |
| Gunasekaran, T. S. and M. Dahlberg (2011). "Prevalence of gastroesophageal reflux symptoms in adolescents: Is there a difference in different racial and ethnic groups?" Diseases of the Esophagus 24(1): 18-24. | Wrong population |
| Gunasekaran, T. S., et al. (2008). "Prevalence and associated features of gastroesophageal reflux symptoms in a Caucasian-predominant adolescent school population." Dig Dis Sci 53(9): 2373-2379. | Wrong population |
| Gupta, S., et al. (2018). "Asthma, GERD and Obesity: Triangle of Inflammation." Indian J Pediatr 85(10): 887-892. | Wrong outcome |
| Ibáñez, J., et al. (1992). "Gastroesophageal reflux in intubated patients receiving enteral nutrition: effect of supine and semirecumbent positions." JPEN J Parenter Enteral Nutr 16(5): 419-422. | Wrong comparator |
| Kase, J. S., et al. (2009). "Risks for common medical conditions experienced by former preterm infants during toddler years." Journal of Perinatal Medicine 37(2): 103-108. | Missing data |
| Lang, J. E., et al. (2016). "Gastro-oesophageal reflux and worse asthma control in obese children: A case of symptom misattribution?" Thorax 71(3): 238-246. | Wrong comparator |
| Mathai, S. S., et al. (2012). "A study of the effect of nasal modes of ventilation on the incidence of gastro-oesophageal reflux in preterm neonates." Medical Journal Armed Forces India 68(1): 12-15. | Missing data |
| Mendes, T. B., et al. (2008). "Risk factors for gastroesophageal reflux disease in very low birth weight infants with bronchopulmonary dysplasia." J Pediatr (Rio J) 84(2): 154-159. | Wrong outcome |
| Mirić, M., et al. (2014). "Lung diffusion capacity in children with respiratory symptoms and untreated GERD." Medical Science Monitor 20: 774-781. | Wrong comparator |
| Monajemzadeh, M., et al. (2013). "Is There any Association Between Passive Smoking and Esophagitis in Pediatrics?" Iranian Journal of Pediatrics 23(2): 194-198. | Wrong outcome |
| Orenstein, S. (2001). "Regurgitation & GERD." J Pediatr Gastroenterol Nutr 32 Suppl 1: S16-18. | Wrong study design |
| Peetsold, M. G., et al. (2010). "Congenital Diaphragmatic Hernia: Long-term Risk of Gastroesophageal Reflux Disease." J Pediatr Gastroenterol Nutr 51(4): 448-453. | Wrong comparator |
| Quitadamo, P., et al. (2020). "Apnea in preterm neonates: what's the role of gastroesophageal reflux? A systematic review." Dig Liver Dis 52(7): 723-729. | Wrong comparator |
| Rodriguez, L., et al. (2022). "Evaluation of Gastroesophageal Reflux Disease in Children on the Autism Spectrum: A Study Evaluating the Tolerance and Utility of the BRAVO Wireless pH Monitoring." J Pediatr Gastroenterol Nutr 75(4): 450-454. | Missing data |
| Ruigómez, A., et al. (2005). "Gastroesophageal reflux disease and asthma -: A longitudinal study in UK general practice." Chest 128(1): 85-93. | Wrong population |
| Ruigómez, A., et al. (2010). "Gastroesophageal reflux disease in children and adolescents in primary care." Scand J Gastroenterol 45(2): 139-146. | Wrong outcome |

### PICO 4 – What is the value of different diagnostic tests for GERD in infants, children, and adolescents?

| Study | Reason for exclusion |
| --- | --- |
| (1991). "The area under pH curve: a single-figure parameter representative of esophageal acid exposure." J Pediatr Surg 26(10): 1259-1260. | Wrong comparator |
| Alatas, F. S., et al. (2023). "Association Between Pediatric Gastroesophageal Reflux Disease Symptom and Quality of Life Questionnaire Score, Endoscopy and Biopsy in Children with Clinical Gastroesophageal Reflux Disease: A Prospective Study." Pediatric Gastroenterology Hepatology & Nutrition 26(4): 173-180. | Wrong outcome |
| Alfattouh, R., et al. (2018). "Investigations for infants admitted with apparent life-threatening event (ALTE): Necessity or liability? Assessment at a tertiary care hospital." Journal of the Pakistan Medical Association 68(10): 1513-1516. | Wrong outcome |
| Andrews, T. M. and N. Orobello (2013). "Histologic versus pH probe results in pediatric laryngopharyngeal reflux." Int J Pediatr Otorhinolaryngol 77(5): 813-816. | Wrong outcome |
| Arasu, T. S., et al. (1980). "The gastroesophageal (GE) scintiscan in detection of GE reflux and pulmonary aspiration in children." Ann Radiol (Paris) 23(3): 187-192. | Wrong outcome |
| Arasu, T. S., et al. (1980). "Gastroesophageal reflux in infants and children comparative accuracy of diagnostic methods." J Pediatr 96(5): 798-803. | Wrong population |
| Baird, D. C., et al. (2015). "Diagnosis and Treatment of Gastroesophageal Reflux in Infants and Children." Am Fam Physician 92(8): 705-714. | Wrong study design |
| Barabino, A., et al. (1995). "Reliability of short-term esophageal pH monitoring versus 24-hour study." J Pediatr Gastroenterol Nutr 21(1): 87-90. | Wrong comparator |
| Berger, D., et al. (1985). "Esophageal and pulmonary scintiscanning in gastroesophageal reflux in children." Prog Pediatr Surg 18: 68-77. | Wrong comparator |
| Blumhagen, J. D. and D. L. Christie (1979). "Gastroesophageal reflux in children: evaluation of the water siphon test." Radiology 131(2): 345-349. | Wrong outcome |
| Carré, I. J. (1985). "Comparison of barium swallow and ultrasound in diagnosis of gastro-oesophageal reflux in children." Br Med J (Clin Res Ed) 291(6495): 606-607. | Wrong study desing |
| Charoenwat, B., et al. (2018). "Transcutaneous esophageal ultrasonography in children with suspected gastroesophageal reflux disease." Journal of the Medical Association of Thailand 101(4): S1-S7. | Wrong outcome |
| Ciorba, A., et al. (2007). "Gastroesophageal reflux and its possible role in the pathogenesis of upper aerodigestive tract disorders." Minerva Gastroenterol Dietol 53(2): 171-180. | Wrong study design |
| Cohen, S. (1976). "The diagnosis and management of gastroesophageal reflux." Adv Intern Med 21: 47-75. | Wrong study design |
| Condino, A. A., et al. (2006). "Evaluation of infantile acid and nonacid gastroesophageal reflux using combined pH monitoring and impedance measurement." J Pediatr Gastroenterol Nutr 42(1): 16-21. | Wrong comparator |
| Corvaglia, L., et al. (2009). "Combined oesophageal impedance-pH monitoring in preterm newborn: comparison of two options for layout analysis." Neurogastroenterol Motil 21(10): 1027-e1081. | Wrong oucome |
| Cresi, F., et al. (2012). "Combined esophageal intraluminal impedance, pH and skin conductance monitoring to detect discomfort in GERD infants." PLoS One 7(8). | Wrong outcome |
| Dalby, K., et al. (2010). "Gastroesophageal reflux disease and eosinophilic esophagitis in infants and children. A study of esophageal pH, multiple intraluminal impedance and endoscopic ultrasound." Scand J Gastroenterol 45(9): 1029-1035. | Wrong outcome |
| Dalby, K., et al. (2007). "Reproducibility of 24-hour combined multiple intraluminal impedance (MII) and pH measurements in infants and children. Evaluation of a diagnostic procedure for gastroesophageal reflux disease." Dig Dis Sci 52(9): 2159-2165. | Wrong comparator |
| Darling, D. B., et al. (1982). "THE CHILD WITH PEPTIC ESOPHAGITIS - A CORRELATION OF RADIOLOGIC SIGNS WITH ESOPHAGEAL PATHOLOGY." Radiology 145(3): 673-676. | Wrong outcome |
| Elbl, B., et al. (2011). "Upper gastrointestinal tract scintigraphy and ultrasonography in diagnosis of gastroesophageal reflux in children." Polish Journal of Radiology 76(1): 63-67. | Wrong outcome |
| Forget, P. P. and M. Meradji (1976). "Contribution of fibreoptic endoscopy to diagnosis and management of children with gastro-oesophageal reflux." Arch Dis Child 51(1): 60-66. | Wrong outcome |
| Formánek, M., et al. (2015). "Comparison of Three Methods Used in the Diagnosis of Extraesophageal Reflux in Children with Chronic Otitis Media with Effusion." Gastroenterology Research and Practice 2015. | Wrong outcome |
| Francavilla, R., et al. (2010). "Comparison of esophageal pH and multichannel intraluminal impedance testing in pediatric patients with suspected gastroesophageal reflux." J Pediatr Gastroenterol Nutr 50(2): 154-160. | Wrong outcome |
| Fujino, J. (2014). "Evaluation of gastroesophageal reflux and gastroesophageal reflux disease with esophageal endoscopy and histology in children." Dokkyo Journal of Medical Sciences 41(2): 155-165. | Wrong population |
| Gomes, H., et al. (2003). "Ultrasonography and gastric emptying in children: Validation of a sonographic method and determination of physiological and pathological patterns." Pediatr Radiol 33(8): 522-529. | Wrong outcome |
| Gomes, H. and B. Menanteau (1991). "Gastro-esophageal reflux: comparative study between sonography and pH monitoring." Pediatr Radiol 21(3): 168-174. | Wrong outcome |
| Gonçalves, E. S., et al. (2020). "Multichannel intraluminal impedance-pH and psychometric properties in gastroesophageal reflux: systematic review." J Pediatr (Rio J) 96(6): 673-685. | Wrong outcome |
| Hojsak, I., et al. (2016). "The role of combined 24-h multichannel intraluminal impedance-pH monitoring in the evaluation of children with gastrointestinal symptoms suggesting gastro-esophageal reflux disease." Neurogastroenterol Motil 28(10): 1488-1493. | Wrong comparator |
| Iannella, G., et al. (2015). "Investigation of pepsin in tears of children with laryngopharyngeal reflux disease." Int J Pediatr Otorhinolaryngol 79(12): 2312-2315. | Wrong population |
| Johnston, B. T., et al. (1996). "Comparison of barium radiology with esophageal pH monitoring in the diagnosis of gastroesophageal reflux disease." Am J Gastroenterol 91(6): 1181-1185. | Wrong population |
| Khatami, A., et al. (2015). "A comparison between gastroesophagheal ultrasonography vs. barium swallow in determining the pattern of gastroesophageal reflux in a pediatric population." Med Ultrason 17(1): 22-27. | Wrong outcome |
| Kwiecień, J., et al. (2010). "The usefulness of esophageal manometry in diagnosing gastroesophageal reflux disease in children." Pediatria Wspolczesna 12(4): 158-162. | Not in English |
| Lupu, V. V., et al. (2018). "Correlation between esophageal pH-metry and esophagitis in gastroesophageal reflux disease in children." Medicine (Baltimore) 97(37): e12042. | Wrong comparator |
| Maholarnkij, S., et al. (2020). "Detection of reflux-symptom association in children with esophageal atresia by video-pH-impedance study." World J Gastroenterol 26(28): 4159-4169. | Wrong population |
| Mancini, V., et al. (2012). "Oesophageal mucosal intercellular space diameter and reflux pattern in childhood erosive and non-erosive reflux disease." Digestive and Liver Disease 44(12): 981-987. | Wrong outcome |
| Mantegazza, C., et al. (2020). "Laryngeal signs and pH-multichannel intraluminal impedance in infants and children: The missing ring: LPR and MII-pH in children." Digestive and Liver Disease 52(9): 1011-1016. | Wrong comparator |
| Meyers, W. F., et al. (1985). "Value of tests for evaluation of gastroesophageal reflux in children." J Pediatr Surg 20(5): 515-520. | Wrong outcome |
| Molle, L. D., et al. (2009). "Nocturnal reflux in children and adolescents with persistent asthma and gastroesophageal reflux." J Asthma 46(4): 347-350. | Wrong outcome |
| Mousa, H., et al. (2005). "Testing the association between gastroesophageal reflux and apnea in infants." J Pediatr Gastroenterol Nutr 41(2): 169-177. | Wrong outcome |
| Nicolai, T. (2001). "Association of apnea and nonacid gastroesophageal reflux in infants: Investigations with the intraluminal impedance technique." Pediatr Pulmonol 31(2): 144-149. | Wrong outcome |
| Nijevitch, A. A., et al. (2004). "Helicobacter pylori Infection and Reflux Esophagitis in Children with Chronic Asthma." J Clin Gastroenterol 38(1): 14-18. | Wrong outcome |
| Orenstein, S. R., et al. (1993). "Scintigraphy versus pH probe for quantification of pediatric gastroesophageal reflux: a study using concurrent multiplexed data and acid feedings." J Nucl Med 34(8): 1228-1234. | Wrong outcome |
| Patra, S., et al. (2011). "Diagnostic modalities for gastro-esophageal reflux in infantile wheezers." J Trop Pediatr 57(2): 99-103. | Wrong outcome |
| Rosen, R., et al. (2014). "The sensitivity of acoustic cough recording relative to intraesophageal pressure recording and patient report during reflux testing." Neurogastroenterol Motil 26(11): 1635-1641. | Wrong outcome |
| Rosen, R., et al. (2013). "The utility of endoscopy and multichannel intraluminal impedance testing in children with cough and wheezing." Pediatr Pulmonol. | Wrong outcome |
| Rosen, R., et al. (2008). "Lipid-laden macrophage index is not an indicator of gastroesophageal reflux-related respiratory disease in children." Pediatrics 121(4): e879-884. | Wrong outcome |
| Rosen, R., et al. (2017). "The Edematous and Erythematous Airway Does Not Denote Pathologic Gastroesophageal Reflux." J Pediatr 183: 127-131. | Wrong intervention |
| Saglani, S., et al. (2006). "Investigation of young children with severe recurrent wheeze: Any clinical benefit?" European Respiratory Journal 27(1): 29-35. | Wrong comparator |
| Salvatore, S., et al. (2016). "Low mean impedance in 24-hour tracings and esophagitis in children: A strong connection." Diseases of the Esophagus 29(1): 10-14. | Wrong outcome |
| Shin, M. S., et al. (2012). "Impedance-pH monitoring and conventional pH monitoring are complementary methods to detect association between gastroesophageal reflux and apnea-related symptoms in preterm infants and neonates." Journal of Maternal-Fetal and Neonatal Medicine 25(11): 2406-2410. | Wrong comparator |
| Skopnik, H., et al. (1996). "Gastroesophageal reflux in infants: Evaluation of a new intraluminal impedance technique." J Pediatr Gastroenterol Nutr 23(5): 591-598. | Wrong outcome |
| Soyer, T., et al. (2013). "Pepsin levels and oxidative stress markers in exhaled breath condensate of patients with gastroesophageal reflux disease." J Pediatr Surg 48(11): 2247-2250. | Wrong outcome |
| Stephen, T. C., et al. (1994). "Diagnosis of gastroesophageal reflux in pediatrics." Journal of the Kentucky Medical Association 92(5): 188-191. | Wrong outcome |
| Thompson, J. K., et al. (1994). "Detection of gastroesophageal reflux: value of barium studies compared with 24-hr pH monitoring." AJR Am J Roentgenol 162(3): 621-626. | Wrong population |
| Tolia, V., et al. (1990). "Lack of correlation between extended pH monitoring and scintigraphy in the evaluation of infants with gastroesophageal reflux." J Lab Clin Med 115(5): 559-563. | Wrong outcome |
| Uslu Kızılkan, N., et al. (2016). "Comparison of multichannel intraluminal impedance-pH monitoring and reflux scintigraphy in pediatric patients with suspected gastroesophageal reflux." World J Gastroenterol 22(43): 9595-9603. | Duplicate |
| Van Der Pol, R. J., et al. (2013). "Diagnostic accuracy of tests in pediatric gastroesophageal reflux disease." Journal of Pediatrics 162(5): 983-987.e984. | Wrong comparator |
| Woodley, F. W. and H. Mousa (2006). "Acid gastroesophageal reflux reports in infants: a comparison of esophageal pH monitoring and multichannel intraluminal impedance measurements." Dig Dis Sci 51(11): 1910-1916. | Wrong outcome |

## Extended Methods for the Systematic Review

### Objective

The objective of this guideline is to provide evidence-based recommendations for the diagnosis and management of gastroesophageal reflux (GER) and gastroesophageal reflux disease (GERD) in infants, children, and adolescents. The guideline aims to support healthcare professionals in delivering consistent and effective care to pediatric patients with GER or GERD, by integrating current evidence and expert consensus.

The primary health intents of this guideline are diagnosis, treatment, and management of GER/GERD in the pediatric population.

The expected benefits include improved accuracy of diagnosis, appropriate use of pharmacological and non-pharmacological treatments, reduction of unnecessary testing, and optimized patient outcomes.

The guideline is intended for use by pediatricians, pediatric gastroenterologists, general practitioners, and other healthcare professionals involved in the care of children with suspected or confirmed GER or GERD.

### Participants and structure

A multidisciplinary panel of 16 physicians with expertise in the diagnosis and management of pediatric GER and GERD and pediatric guidelines was convened by the Presidents of the Italian Society of Pediatrics (SIP) (A.S.) and of Italian Society of Pediatric Gastroenterology Hepatology and Nutrition (SIGENP) (C.R.). The President (S.C.) of the Italian Association of Neonates with reflux (AINER) also participated to the first online meetings providing comments on the content of this guideline, on the identified PICOs and on unmet needs of young patients. She also read and commented on the draft of the document and the final manuscript. An external Agency (CREA Sanità) with experience in data analysis and applied healthcare research participated to all the meetings and the Evidence Review Team (ERT), composed of experts in evidence synthesis conducted a preliminary search of international guidelines on the diagnosis and management of GER and GERD in children to support the panel in the development of PICO questions. A systematic literature search was performed in PubMed, Embase, Web of Science, and Google Scholar on February 22, 2024 (Additional File 1). The quality of the identified guidelines was assessed using the AGREE II tool [1]. PICO questions from the included guidelines were extracted, analyzed, and presented to the panel, which then used a Delphi process to prioritize them and define a final list of key questions considered critical to address. The panel identified eight key questions concerning infants, children and adolescents (Table 1):

**Table 1. List of clinical questions identified as relevant for this guideline**

| Questions | PICO |
| --- | --- |
| 1. What is the definition of GER and GERD? | NO |
| 2. What are the signs and symptoms associated with GER and GERD? | YES |
| 3. What are the risk factors for GERD? | YES |
| 4. What is the value of different diagnostic testing for GERD? | YES |
| 5. What is the evidence of effectiveness of pharmacologic treatment for GER and GERD? | YES |
| 6. What is the effectiveness of different non-pharmacologic treatment options for GER and GERD? | YES |
| 7. What is the indication and the effectiveness of different surgical/endoscopic treatment options for GERD? | YES |
| 8. What is the prognosis of GER and GERD and what are prognostic factors? | NO |

Questions 2 through 6 were structured using the PICO format, while questions 1 and 8 were formulated as narrative clinical questions, due to their descriptive nature.

Subsequently, the ERT, in collaboration with the panel, developed search strategies tailored to each key question. Systematic literature searches were conducted in PubMed, Embase, and Web of Science on May 11–12, 2024. The complete search strategies and strings with MESH terms are provided in Additional File 1.

Manual retrieval of additional original studies or reviews from references of the papers identified by the systematic review was also performed by all authors and, whenever deemed useful for the purpose of this document the studies were included in the related PICO evidence summary.

### Systematic review of international guidelines on GER and GERD

The study selection process was carried out independently by two reviewers in two phases. Initially, titles and abstracts were screened according to predefined inclusion criteria: English-language guidelines focusing on the diagnosis of GER and GERD in pediatric populations. Subsequently, potentially eligible full-text articles were assessed. Any disagreements between reviewers were resolved through discussion. Data extraction was performed by one reviewer and verified by a second. The methodological quality of guidelines was assessed by three independent reviewers through the AGREE II tool. The literature selection process is illustrated in Additional File 1 (PRISMA 2020 Flow Diagram). A list of excluded studies with reasons for exclusion is provided in the Additional File 1.

The literature search initially identified 350 records. After removing duplicates, 217 records were screened by title and abstract, and 30 full-text articles were assessed for eligibility. Of these, 27 were excluded, and 3 [2-4] were included in the final analysis. The first document [3] dated 2018, was a clinical practice guideline on pediatric GER and GERD, jointly developed by the North American Society for Pediatric Gastroenterology, Hepatology, and Nutrition (NASPGHAN) and the European Society for Pediatric Gastroenterology, Hepatology, and Nutrition (ESPGHAN). This work represents an update of their previous joint guideline published in 2009 [5].

The second guideline identified was developed by the National Institute for Health and Care Excellence (NICE) in 2015 [2], and updated in 2019 [6]. It addresses the diagnosis and management of GERD in children and young people.

The third document [4] was a guideline by the Society of American Gastrointestinal and Endoscopic Surgeons (SAGES), focusing on the surgical treatment of GERD in both adult and pediatric patients.

The methodological quality of the three guidelines varied. The NASPGHAN/ESPGHAN guideline was rated as low quality (AGREE II total score: 55/100; Domain 3 score: 40/100). The SAGES guideline was considered to be of moderate quality (AGREE II total score: 60/100; Domain 3 score: 59/100). The NICE guideline received the highest rating, judged to be of good quality (AGREE II total score: 73/100; Domain 3 score: 72/100).

### Systematic reviews on the eight key questions

Eight systematic reviews were conducted, one for each key question. A study protocol for these systematic reviews was registered in the PROSPERO database (CRD420251041380). All reviews were conducted following Cochrane methodology [7], and reported in accordance with the PRISMA 2020 statement [8, 9]. The guideline itself was reported in accordance with the AGREE Reporting Checklist [10].

#### Eligibility criteria

**Population**: for key questions 1-4, we included infants, children, and adolescents (0–18 years) presenting with signs and symptoms suggestive of GER or GERD, irrespective of formal diagnosis. For key questions 5-8, we included infants, children, and adolescents (0–18 years) with a confirmed diagnosis of GER or GERD, according to any recognized clinical or diagnostic criteria.

**Interventions or exposures**:

Key Question 1 (Definition): no specific intervention or exposure. We included studies providing definitions of GER or GERD.

PICO Question 2 (Signs and symptoms): the exposure was the presence of specific signs or symptoms potentially associated with GER/GERD, including: heartburn, difficulty swallowing, nausea, chronic cough, bloating, refusal to feed, regurgitation, chest pain, wheezing, sore throat, dysphagia, laryngitis, vomiting, weight loss, pneumonia, dental erosion, otitis, apnea, and Sandifer syndrome.

PICO Question 3 (Risk factors): the exposure was the presence of potential risk factors associated with GERD development, including: neurodevelopmental disorders, cerebral palsy, prematurity, family history of GERD, surgical or congenital abnormalities, congenital heart disease, chronic lung disease, and obesity.

PICO Question 4 (Diagnostic testing): the intervention was the use of diagnostic tests for GERD (e.g., esophageal pH monitoring, pH-impedance (pH-MII), endoscopy, esophageal manometry, scintigraphy, ultrasonography, and clinical diagnostic tools or questionnaires).

PICO Question 5 (Pharmacological treatment): the intervention was pharmacologic therapy for GERD (e.g., proton pump inhibitors, H2-receptor antagonists, prokinetic agents, antacids).

PICO Question 6 (Non-pharmacological treatment): the intervention included non-pharmacological approaches (e.g., dietary modifications, feeding interventions, positional therapy, behavioral therapy, alginates, massage therapy, and complementary medicine).

PICO Question 7 (Surgical/endoscopic treatment): the intervention consisted of surgical or endoscopic treatments for GERD (e.g., fundoplication, endoscopic procedures).

Key Question 8 (Prognosis): no specific intervention. We included studies evaluating the natural history or prognostic factors of GER/GERD.

**Comparators or controls**

Where applicable, comparators included placebo, no treatment, usual care, or alternative diagnostic or therapeutic interventions.

**Study design**

Key Question 1 (Definition): Guidelines, consensus statements, and observational studies (cohort, case-control, cross-sectional).

PICO Questions 2-3 (Signs/symptoms, Risk factors): Systematic reviews; observational studies (cohort, case-control, cross-sectional).

PICO Question 4 (Diagnostic testing): Systematic reviews/meta-analyses of diagnostic accuracy studies or RCTs; primary diagnostic accuracy studies (cross-sectional or cohort) and RCTs.

PICO Questions 5–7 (Pharmacologic, Non-Pharmacologic, Surgical/Endoscopic): Systematic reviews/meta-analyses of RCTs; RCTs.

Key Question 8 (Prognosis): Systematic reviews and observational studies (cohort, case-control, cross-sectional).

**Outcomes**

Key Question 1 (Definition): Definitions and criteria used to define GER and GERD in infants, children, and adolescents.

PICO Question 2 (Signs and symptoms): Diagnostic accuracy (e.g., sensitivity, specificity) of individual signs and symptoms associated with GER/GERD.

PICO Question 3 (Risk factors): Association between potential risk factors and GERD development (measured as odds ratios [OR], relative risks [RR], or hazard ratios [HR]).

PICO Question 4 (Diagnostic testing): Diagnostic accuracy of different tests (e.g., sensitivity, specificity, positive and negative predictive values, likelihood ratios).

PICO Question 5 (Pharmacological treatment): Effectiveness and safety of pharmacological treatments in improving GERD symptoms or healing esophagitis (measured by symptom resolution rates, esophagitis healing rates, adverse events).

PICO Question 6 (Non-pharmacological treatment): Effectiveness and safety of non-pharmacological treatments in reducing GERD symptoms or preventing GERD complications (measured by symptom improvement rates, quality of life scores, adverse events).

PICO Question 7 (Surgical/endoscopic treatment): Effectiveness and safety of surgical and endoscopic interventions (e.g., symptom resolution, esophagitis healing, reintervention rates, adverse events).

Key Question 8 (Prognosis): Natural history and prognosis of GER/GERD (e.g., rates of spontaneous resolution, persistence, or progression; factors associated with prognosis).

**Context**

Studies conducted in any healthcare setting (e.g., hospital, outpatient clinic, primary care, community settings) were included. No restrictions were applied based on geographic location, healthcare system, or country income level.

#### Review process

The literature selection process was performed independently by pairs of reviewers for each key question. The first selection was based on title and abstract screening. Articles selected in this phase were subsequently assessed for eligibility. In both phases, disagreements were resolved by consensus. Data extraction and quality assessment were performed by one reviewer and verified by another reviewer. Quality assessment was performed using tools specific to study design. The quality of observational studies and systematic reviews was assessed by the JBI checklists [11], while RCTs were evaluated using the Cochrane RoB 2 tool [12].

Subgroups of at least two participating authors focused on the different PICOs and topics, reviewed the provided list of documents and related references, produced a written text on the summary of evidence with recommendations that were discussed by all authors during the meetings and voted online.

#### Statistical analysis

Data for all key questions were synthesized using GRADE evidence profile tables and narrative summaries. When studies were sufficiently homogeneous in terms of population, interventions, comparators, and outcomes for each comparison, meta-analyses were performed using random-effects models. Effect measures included risk ratios (RR), odds ratios (OR), hazard ratios (HR), mean differences (MD), or standardized mean differences (SMD), as reported in the included studies. When meta-analyses were conducted, statistical heterogeneity was assessed using the I² statistic. Subgroup and sensitivity analyses were not performed due to insufficient data being available. P-values < 0.05 were considered statistically significant.

#### Certainty assessment

The GRADE (Grading of Recommendations Assessment, Development and Evaluation) approach [13] was applied to assess the certainty of evidence for PICO questions 2 through 7. The certainty of evidence was rated as high, moderate, low, or very low, based on the domains of risk of bias, inconsistency, indirectness, imprecision, and publication bias.

Evidence from RCTs was initially considered high certainty and downgraded by one level for serious limitations, or by two levels for very serious limitations in any of the above domains. Conversely, evidence from observational studies started at low certainty.

For the risk of bias domain, we downgraded by one level if at least one study was judged having some concerns, while we downgraded by two levels if most of studies had a high risk of bias in more than one domain. Inconsistency was evaluated based on the presence of unexplained heterogeneity across study results. For indirectness, we evaluated whether the populations, interventions, comparators and outcomes considered in the included studies correspond to those planned in the PICO questions. We downgraded for imprecision if the effect estimates are from studies with a small sample size and wide confidence intervals. Publication bias was not assessed due to the limited number of studies for each comparison.

Factors that may increase the certainty level, such as large magnitude of an effect, dose-response gradient, and effect of plausible residual confounding, were also considered. GRADE Evidence profile tables were created by GRADEpro GDT software [14].

#### Evidence to Decision framework

A formal Evidence to Decision (EtD) framework was applied for PICO questions 5 to 7. This approach was not deemed applicable to the remaining key questions. In accordance with the EtD methodology, the following domains were systematically evaluated:

- Problem: Is the health problem a priority?
- Desirable Effects: How substantial are the desirable anticipated effects?
- Undesirable Effects: How substantial are the undesirable anticipated effects?
- Certainty of evidence: What is the overall certainty of the evidence of effects?
- Values: Is there important uncertainty about or variability in how much people value the main outcomes?
- Balance of effects: Does the balance between desirable and undesirable effects favor the intervention or the comparison?
- Resources required: What resources are needed to implement the intervention?
- Certainty of evidence of required resources: What is the certainty of the evidence of resource requirements (costs)?
- Cost effectiveness: Does the cost-effectiveness of the intervention favor the intervention or the comparison?
- Equity: What would be the impact on health equity?
- Acceptability: Is the intervention acceptable to key stakeholders?
- Feasibility: Is the intervention feasible to implement?

Based on the judgements given on these domains, the expert panel proposed the strength of recommendations (SoR) as strong or weak against or weak or strong in favor of the intervention.

#### Consensus process

Recommendations, including their direction and strength, were developed through a combination of structured discussion and iterative voting. A two-round Delphi process was conducted between April and May 2025. Consensus was defined as ≥80% agreement or disagreement (scores 7–9 or scores 1-3 on a 9-point Likert scale).

The strength and direction of each recommendation were also explored through voting, with participants selecting one of the four predefined categories:

- Strong recommendation against the intervention
- Weak recommendation against the intervention
- Weak recommendation for the intervention
- Strong recommendation for the intervention

Final consensus on both the content and the strength of each recommendation was achieved through discussion during the plenary session, with all panel members participating.

A total of 40 recommendations related to PICO questions 2 through 7 were formulated and finalized through this combined process.

#### External review

An external review was conducted to ensure the validity, applicability, and clarity of both the guideline text and the recommendations. The draft guideline was shared with three pediatric external experts on GERD (Osvaldo Borrelli, Yvan Vandenplas, Mario C. Vieira) and with the Presidents of Italian Federation of Societies of Digestive Diseases (FISMAD), of the Italian Pediatric Society of Neonatology (SIN) and of Respiratory Diseases (SIMRI) and of an Association of Italian Family Pediatricians who were not involved in the development process. These included primary care and hospital pediatricians, pediatric gastroenterologists, adult gastroenterologists and representatives from relevant Italian scientific societies.

Reviewers were selected based on their clinical or methodological expertise and institutional role.

They were invited to provide comments and suggestions, focusing on the clarity, relevance, feasibility, and potential impact of each recommendation.

All feedback received was summarized and discussed during a plenary meeting of the guideline panel. Where appropriate, relevant suggestions and criticisms were incorporated into the final version of the recommendations.

#### Facilitators and Barriers to Application

Facilitators and barriers to the implementation of the guideline recommendations were considered qualitatively by the panel during the development process, particularly within the Evidence to Decision (EtD) framework for PICO questions 5 to 7. Although no formal pilot testing or surveys with stakeholders were conducted, the panel drew on their clinical experience across diverse healthcare settings in Italy, including tertiary hospitals, community hospitals, and outpatient pediatric services.

Facilitators identified included:

- Broad availability of diagnostic tools (e.g., esophageal pH-impedance, endoscopy) in referral centers
- Existing clinical awareness of GER/GERD in pediatrics
- High relevance and clarity of the structured recommendations
- Multidisciplinary interest in standardized management approaches

Barriers noted included:

- Limited access to specialized testing in some geographic areas
- Variability in healthcare professionals' familiarity with non-pharmacological management strategies
- Potential overuse or misuse of pharmacological treatments
- Lack of awareness of updated evidence among general practitioners

These considerations were discussed during the formulation of recommendations and helped guide decisions on the strength of recommendations, as well as the inclusion of non-pharmacological and context-sensitive options where evidence supported them. Where barriers were identified, recommendations were worded to allow flexibility based on available resources and clinical judgment.

#### Implementation Tools and Advice

To support the practical application of this guideline, a summary table was developed (Table…) presenting each PICO question alongside the corresponding recommendations and their strength and direction. Two figures (Fig… and Fig …) were created to represent diagnostic and therapeutic algorithm for pediatric GERD and strength of recommendations for or against different treatment in infants, children and adolescents. These tools are intended to facilitate quick reference and integration of the guideline content into clinical decision-making.

The summary was designed for use by pediatric healthcare professionals across various settings, including hospitals and outpatient services. It can assist in translating evidence-based recommendations into practice and in promoting consistency in the management of GER and GERD in infants, children, and adolescents.

#### Resource Implications

No formal systematic search of economic evaluations or cost-effectiveness studies was conducted or retrieved as part of this guideline. However, considerations regarding potential resource implications were discussed by the panel during the formulation of recommendations related to PICO questions 5–7 (pharmacological, non-pharmacological, and surgical/endoscopic treatments). These aspects were addressed within the Evidence to Decision (EtD) tables, which included a domain specifically focused on resource use. The panel considered, when applicable, the availability and costs of interventions in the Italian healthcare context, including drug acquisition, hospital-based procedures, and access to specialized services. While no quantitative cost data were formally incorporated, qualitative judgments about the feasibility and sustainability of implementing each intervention were explicitly documented and contributed to the final formulation of the recommendations.

#### Monitoring and Auditing Criteria

At the time of publication, no formal set of monitoring or auditing tools has been developed for this guideline. However, the panel acknowledges the importance of tracking the implementation and real-world impact of the recommendations. To this end, a set of core process and outcome indicators will be proposed during the dissemination phase, in collaboration with national pediatric and gastroenterology societies and patients’ associations.

Potential indicators could include:

- Adherence to recommended diagnostic pathways (e.g., appropriate use of pH-impedance monitoring or endoscopy)
- Appropriate prescription of pharmacological treatments (e.g., proton pump inhibitors limited to evidence-based indications and durations)
- Reduction in the use of ineffective or non-recommended interventions
- Surgical referrals based on agreed clinical criteria

These indicators can be used by institutions for local audits, quality improvement initiatives, and benchmarking practices. Data may be collected through surveys, retrospective chart reviews, electronic medical record queries, or standardized data collection forms.

Where feasible, annual or biennial audits are encouraged to assess adherence and identify implementation barriers. The panel supports the integration of these indicators into national and international networks, to facilitate continuous monitoring.

#### Updating procedure

This guideline will be updated regularly to ensure its continued relevance in light of emerging evidence and evolving clinical practice. The development group has established a plan to review and update the guideline every five years, or earlier if substantial new evidence becomes available that may warrant changes to current recommendations.

The same methodology used for the present guideline—based on systematic reviews, GRADE assessments, and the Evidence to Decision (EtD) framework—will be applied during the updating process.

The literature searches will be updated starting from the date of the last search conducted for this guideline. In the update process, the working group will also assess the continued relevance of the existing PICO questions, and may decide to revise, remove, or introduce new questions based on clinical need and stakeholder input.

The update will ideally be carried out by the current guideline development group or an appointed successor team, in collaboration with methodological experts.

# References

1. Brouwers MC, Kho ME, Browman GP, Burgers JS, Cluzeau F, Feder G, et al. AGREE II: advancing guideline development, reporting and evaluation in health care. Cmaj. 2010;182(18):E839-42.

2. Davies I, Burman-Roy S, Murphy MS. Gastro-oesophageal reflux disease in children: NICE guidance. Bmj. 2015;350:g7703.

3. Rosen R, Vandenplas Y, Singendonk M, Cabana M, DiLorenzo C, Gottrand F, et al. Pediatric Gastroesophageal Reflux Clinical Practice Guidelines: Joint Recommendations of the North American Society for Pediatric Gastroenterology, Hepatology, and Nutrition and the European Society for Pediatric Gastroenterology, Hepatology, and Nutrition. J Pediatr Gastroenterol Nutr. 2018;66(3):516-54.

4. Slater BJ, Dirks RC, McKinley SK, Ansari MT, Kohn GP, Thosani N, et al. SAGES guidelines for the surgical treatment of gastroesophageal reflux (GERD). Surg Endosc. 2021;35(9):4903-17.

5. Vandenplas Y, Rudolph CD, Di Lorenzo C, Hassall E, Liptak G, Mazur L, et al. Pediatric gastroesophageal reflux clinical practice guidelines: Joint recommendations of the North American Society for Pediatric Gastroenterology, Hepatology, and Nutrition (NASPGHAN) and the European Society for Pediatric Gastroenterology, Hepatology, and Nutrition (ESPGHAN). J Pediatr Gastroenterol Nutr. 2009;49(4):498-547.

6. NICE. Gastro-oesophageal reflux disease in children and young people: diagnosis and management. NICE guideline. Reference number: NG1. Published: 14 January 2015. Last updated: 09 October 2019. Available at: <https://www.nice.org.uk/guidance/ng1> (accessed: 25.05.2025). 2015.

7. Higgins JPT, Thomas J, Chandler J, Cumpston M, Li T, Page MJ, Welch VA (editors). Cochrane Handbook for Systematic Reviews of Interventions version 6.5 (updated August 2024). Cochrane, 2024. Available from [www.training.cochrane.org/handbook](http://www.training.cochrane.org/handbook) (accessed: 25.05.2025).

8. Page MJ, McKenzie JE, Bossuyt PM, Boutron I, Hoffmann TC, Mulrow CD, et al. The PRISMA 2020 statement: an updated guideline for reporting systematic reviews. Bmj. 2021;372:n71.

9. Page MJ, Moher D, Bossuyt PM, Boutron I, Hoffmann TC, Mulrow CD, et al. PRISMA 2020 explanation and elaboration: updated guidance and exemplars for reporting systematic reviews. Bmj. 2021;372:n160.

10. Brouwers MC, Kerkvliet K, Spithoff K. The AGREE Reporting Checklist: a tool to improve reporting of clinical practice guidelines. Bmj. 2016;352:i1152.

11. Aromataris E, Lockwood C, Porritt K, Pilla B, Jordan Z, editors. JBI Manual for Evidence Synthesis. JBI; 2024. Available from: <https://synthesismanual.jbi.global>. <https://doi.org/10.46658/JBIMES-24-01> (accessed: 25.05.2025).

12. Sterne JAC, Savović J, Page MJ, Elbers RG, Blencowe NS, Boutron I, et al. RoB 2: a revised tool for assessing risk of bias in randomised trials. Bmj. 2019;366:l4898.

13. Schünemann HJ, Higgins JP, Vist GE, Glasziou P, Akl EA, Skoetz N, et al. Chapter 14: Completing ‘Summary of findings’ tables and grading the certainty of the evidence. In: Higgins JPT, Thomas J, Chandler J, Cumpston M, Li T, Page MJ, Welch VA (editors). Cochrane Handbook for Systematic Reviews of Interventions version 6.5 (updated August 2024). Cochrane, 2024. Available from [www.training.cochrane.org/handbook](http://www.training.cochrane.org/handbook), accessed 10 May 2025.

14. McMaster University (developed by Evidence Prime) GRADEpro GDT. Hamilton (ON): McMaster University (developed by Evidence Prime), accessed 10 May 2025. Available at [www.gradepro.org](http://www.gradepro.org).
