# Supplementary material for: Guidelines on diagnosis and management of gastroesophageal reflux disease in infants, children and adolescents: a joint consensus from Italian pediatric societies (SIP and SIGENP) -part I. diagnosis
Source: Ital J Pediatr. 2026 Apr 11;52:91. doi: 10.1186/s13052-026-02218-5 (PMC13185181; doi:10.1186/s13052-026-02218-5)
Supplement: Supplementary file 2 — Supplementary Material 2 [file 13052_2026_2218_MOESM2_ESM.docx]

# Additional File 2

## Characteristics of included studies

### PICO 1– What is the definition of gastroesophageal reflux (GER) and gastroesophageal reflux disease (GERD) in infants, children, and adolescents?

| Study ID | Country | Society/Organization | Study Design | Study Outcome (Definition) |
| --- | --- | --- | --- | --- |
| Sherman 2009 |  |  | Global, Evidence-Based Consensus (systematic review + Delphi + grade assessment) | GERD is present when reflux of gastric content causes troublesome symptoms and / or complications, but this definition is complicated by unreliable reporting of symptoms in children under the age of ~ 8 years |
| Singendonk 2017 |  |  | Systematic Review | - |
| Vakil 2006 | Multiple countries | Global Consensus Group | Global Evidence-Based Consensus (systematic review + Delphi) | GERD is a condition which develops when the reflux of stomach contents causes troublesome symptoms and/or complications. |
| Davies et al., 2015 | United Kingdom | NICE (National Institute for Health and Care Excellence) | Guideline | "Gastro-esophageal reflux (GOR) is the passage of gastric contents into the esophagus. It is a common physiological event that can happen at all ages from infancy to old age and is often asymptomatic. It occurs more frequently after feeds/meals. In many infants, GOR is associated with a tendency to ‘overt regurgitation’ – the visible regurgitation of feeds. Gastro-oesophageal reflux disease (GORD) refers to gastro-oesophageal reflux that causes.  symptoms (for example, discomfort or pain) severe enough to merit medical treatment, or to gastro-oesophageal reflux-associated complications (such as oesophagitis or pulmonary aspiration). In adults, the term GORD is often used more narrowly, referring specifically to reflux oesophagitis" |
| Rosen et al., 2018* | USA/Europe | NASPGHAN/ESPGHAN | Guideline | "GER: the passage of gastric contents into the esophagus with or without regurgitation and vomiting.  GERD: when GER leads to troublesome symptoms that affect daily functioning and/or complications" |
| Gold and Sherman 2008 | USA (Global) | - | Consensus | GERD is present when reflux of gastric contents causes troublesome symptoms and/or complications, but this definition is complicated by unreliable reporting of symptoms by children under 8 years |
| Vandenplas 1994 | Europe | European Society of Paediatric Gastroenterology and Nutrition | Consensus | GER is best defined as the involuntary passage of gastric contents into the esophagus. The origin of the gastric contents can vary, including saliva, ingested food and drink, gastric secretions, and pancreatic or biliary secretions that have first refluxed into the stomach (duodenogastric reflux). The regurgitated or vomited material can be considered the tip of the iceberg of GER. |
| *Update of Vandenplas et al., 2009 (JPGN 49:498–547, 2009.) | | | | |
| Abbreviations: ESPGHAN: European Society for Paediatric Gastroenterology Hepatology and Nutrition; GER: Gastroesophageal reflux; GERD: gastroesophageal reflux disease; GOR: Gastro-esophageal reflux; NASPGHAN: North American Society for Pediatric Gastroenterology, Hepatology & Nutrition; NICE: National Institute for Health and Care Excellence. | | | | |

### PICO 2– What are the signs and symptoms indicative of GERD in infants, children, and adolescents?

| Study ID | Country | Study Design | Sample Size | Population | Intervention (Sign or symptom of interest) | Outcome |
| --- | --- | --- | --- | --- | --- | --- |
| Ashorn 2002 | Finland | Observational: retrospective chart review | 76 | Children (mean age 6.8 yr; range 2–17 yr) | Abdominal pain, heartburn, regurgitation and vomiting | Abdominal pain (63%), heartburn (34%), regurgitation (22%), vomiting (16%) retrosternal pain (18%), respiratory symptoms (29%). pH-recording was normal in 17/67 subjects, slightly pathological in 33 and severe reflux was diagnosed in 13 patients. |
| Bellomo-Brandao 2021 | Brazil | Observational: cross-sectional study | 54 | Infants (median age: 36 days) | Brief resolved unexplained events (BRUE) | GERD diagnosed by the pHmetry was found in one third of infants that experiencing high-risk BRUE. |
| Borrelli 2011 | United Kingdom | Observational prospective study | 45 | Children (1-16 yr) | Chronic cough | 24 children had abnormal tests based on quantitative and/or qualitative analysis and were classified as having cough-related-reflux (CRR). |
| Bouchard 1999 | Canada | Observational: retrospective chart review | 105 | Children | Stridor, laryngomalacia, recurrent otitis media, laryngitis, and dysphonia | Overall, 41% of the pH study results were positive for gastroesophageal reflux. Only patients referred to stridor (58%), laryngomalacia (61%), and laryngitis (56%) had greater than 50% incidence of GER demonstrated on pH study. Patients with recurrent otitis media, dysphonia, and laryngeal papillomatosis had GER in only l%, 14%, and 25% of the cases, respectively. |
| Campanozzi 2009 | Italy | Observational: prospective survey | 313 | Children (3.8 ± 2.7 months) | Children with infant regurgitation | 313 children (12%; 147 girls) received the diagnosis of infant regurgitation. Vomiting was present in 34 of 313 patients. A follow-up evaluation was available for 210 children. 1 (0.5%) of 210 patients had developed GER disease with esophagitis endoscopically and histologically proven. |
| Chen 1991 | China | Observational prospective study | 23 | Children (3-25 mo) | Children with recurrent pneumonia | 21/23 showed abnormal GER on 24-h Ph monitoring. |
| Chopra 1995 | India | Observational study | 80 | Children (9 mo - 12 yr) | Bronchial asthma | Twenty-five patients had symptoms suggestive of GER in the form of retrosternal pain (in older children), vomiting and exacerbation of symptoms on lying down or after meals. Of thirty-one scintiscan positive patients, fifteen (48.39%) patients had symptoms of GER while ten out of 49 (20.4%) scintiscan negative patients gave similar symtoms. |
| Chougran 2021 | USA | Systematic review and meta-analysis | 15 studies (1435 children) | Children (>5 yr) | Croup | The reported prevalence of GERD in these studies ranges from 5% to 87%. |
| Dahshan 2002 | USA | Observational prospective study | 37 | Children (2-18 yr) | Dental erosion | Endoscopic and histological evaluation of the distal esophageal biopsy specimens revealed that 24 patients had GERD. Of these, dental erosions were present in 20 patients (20/24, 83.3%; P ≤ .001). |
| Ferenchak 1994 | USA | Observational study | 8 | Infants (2-32 weeks) | Discomfort, emission, yawn, stretch, stridor, mouthing, sneezing, hiccupping, thumb-sucking, and coughing-gagging | Tapes from two of the 10 subjects could not be analyzed due to virtually continuous reflux, with lack of onsets and lack of no-reflux time, and are thus not considered further. |
| Fishbein 2012 | USA | Observational: retrospective chart review | 67 | infants (0-1 yr) | Spitting up, gagging, projectile vomiting, back arching, coughing, cyanosis, reactive airway disease, positive pH monitoring, retching, “positive” esophagogastrogram (episode of reflux visualized during examination), or presently treated with antireflux medication (prokinetic, H2 blocker, proton pump inhibitor | In the eight remaining subjects, six of the 10 behaviors (discomfort, emission, yawn, stretch, stridor, and mouthing) were significantly associated with onsets of gastroesophageal reflux episodes when the events were aggregated across subject. Four further behaviors (sneezing, hiccupping, thumb-sucking, and coughing-gagging) were significantly associated with onset of reflux events in one or two subjects each." |
| Foroutan 2002 | Iran | Observational retrospective study | 52 | Children (4 mo - 10 yr) | Chronic cough, recurrent pneumonia, asthma, and respiratory distress | A swallow evaluation was recommended in 39 of 67 infants with abnormalities noted in all. Swallowing abnormalities were more frequent and clustered in infants with GERD-like symptoms vs controls. MBS was abnormal in 16 of 17 qualifying infants. |
| Garza 2011 | USA | Observational retrospective study | 186 | Infants (mean age 5.4 ± 3.4 mo) | Infants suspected of having GERD (frequent regurgitation/vomiting, apparent life-threatening events, feeding disorder, respiratory symptoms) | 24 hour esophageal PH monitoring revealed GER in 22 (42.2%) patients as a cause of their chronic respiratory symptoms, while (30 (57.7%) children did not show any evidence of GER. GER was detected in 11 of 24 (45.7%) patients with chronic cough. Thirty-three patients presented with recurrent pneumonia, 13(39.9%) of whom had GER. In 8 patients with asthma, GER was found in 4 cases. None of the 6 patients with respiratory distress had GERD. |
| Greifer 2012 | USA | Observational: retrospective chart review | 63 | Children (0-21 yr) | Cough/asthma, hoarseness, vocal cord nodules and pharyngitis | A total of 4159 symptoms were recorded during the studies; 1504 (36%) were associated with a reflux event. |
| Gupta 2006 | USA | Observational study | 129 | Children (1-17 yr) | regurgitation/vomiting, abdominal pain, and cough​ | Using the DeMeester pH score for pathologic GER, only six children (9.5%) met this criteria. Using the impedance criteria, only 10 out of the 63 patients (15.8%) had an abnormal evaluation. A symptom index was generated in 46 out of the 63 patients (73%). In the patients where a symptom index was recorded, only seven patients (15.2%) were found to have a positive association between their recorded symptom and a reflux event. |
| Karabel 2014 | Turkey | Observational study | 270 | Children (2 mo - 14 yr) | Chronic cough | The most frequently reported symptoms in patients with​ NEE were abdominal pain (70%), regurgitation/vomiting​ (69%), cough (69%), and heartburn (68%), the majority of​ which were mild to moderate in severity. Among​ patients with EE, the two most frequently reported symptoms​ were regurgitation (69%) and abdominal pain (67%). Other​ commonly reported symptoms in patients with EE were​ cough (51%), anorexia/feed refusal (49%) and heartburn (47%). |
| Keles 2004 | Turkey | Observational prospective study | 25 | Children | Chronic otitis media | The total of 270 patients were included in the study included 43.3% (n = 153) females with a mean age of 6.5 ± 2.3 years (7 months to 17 years). After a 1-year follow-up of patients, we determined that the most common etiologic factors were asthma (27%), asthma-like syndrome (15.5%) and gastroesophageal reflux (10%). |
| Kiran 2023 | India | Observational study | 77 | Children (0-16 yr) | Chronic cough | In the study group, the frequencies of gastroesophageal reflux (GER) were 64%. |
| Kohelet 2004 | Israel | Observational retrospective study | 134 | "Infants  Group 1 (preterm infant group, n=45), Group 2 (term infant group, n=89)" | Presence of one or more persistent signs suggestive of GER: apnea, bradycardia, or cyanosis, vomiting and regurgitation. | Only 1 in 77 children was diagnosed with GERD. |
| Koivusalo 2011 | Finland | Observational retrospective study | 87 | Infants (<12 mo) | Admission for apneic episodes | GER was present in 18/45 (40%) of Group 1 infants, and in 44/89 (49.5%) Group 2 infants (p=0.3). |
| Kosec 2020 | Croatia | Observational retrospective study | 89 | Children (median age: 12 yr) | Pediatric patients with symptoms suggestive of GERD: epigastric pain, occasional nausea, regurgitation, tasting acid in the oral cavity, chronic cough, hoarseness of voice, frequent throat clearing. | Esophageal pH monitoring was done to 58/87 (67%) patients, of whom 53/58 (91%) had a pathological finding. |
| Kotsis 2009 | Greece | Observational study | 187 | Children (mean age: 18 mo) | "Children with symptoms and signs associated with GERD - Group A (control group, without GERD): 49 children - Group B (with mild-moderate GERD): 78 children - Group C (with severe GERD): 60 children" | 52 children had positive diagnostic criteria for GERD, and 50 of these 52 had positive criteria for PLPR. "In Group A (controls without GERD), only 6/49 children (12.2%) had RAOM. In Group B (mild to moderate GERD), 11 /78 children (14.1%) had RAOM before anti-reflux treatment. In Group C (severe GERD), 19/60 children (31.7%) had RAOM (p = 0.014)." |
| Lechien 2020 | Not applicable | Systematic review | 11 clinical studies including 720 reflux and 319 healthy children | Children (1-18 yr) | Association between reflux (GERD or LPR) and the following dental disorders: mucosa inflammation (e.g. gingivitis and periodontitis), dental erosion and caries | The mean prevalence of dental erosion in reflux children was 57% (17–98%), which was higher than the mean prevalence of erosion in healthy children (12%; 6–19%). |
| Li 2022 | Not applicable | Systematic review and meta-analysis | 13 studies included (12 in MA) | Children (1-18 yr) | Association between GERD and dental erosion | There were strong associations between GERD and enamel erosion with the subject as unit (odds ratio 4.46; 95% confidence interval (CI) 1.93–10.30; n = 6774); dentine erosion with the subject as unit (odds ratio 13.39; 95% CI 4.17–42.94; n = 371). |
| Lin 2008 | Taiwan | Observational retrospective study | 103 | Children (mean age, 13 yr; range, 4−17 yr) | Children with chest pain who were evaluated in a pediatric emergency department (ED) | Six (5.8%) patients underwent panendoscopy because of epigastric pain. Gastroesophageal reflux was found in three of them. |
| Lupu 2021 | Romania | Observational retrospective study | 85 | Children (0-18 yr) | Children with recurrent wheezing | Gastroesophageal reflux was present in 71 children (83.5%). |
| Lupu 2023 | Romania | Observational retrospective study | 53 | Children (6-162 mo) | Children with recurrent respiratory tract infections | 41 children were found with a positive Boix-Ochoa score. |
| Mercado-Deane 2001 | USA | Observational prospective study | 472 | Infants (< 1 yr) | Infants with vomiting and respiratory symptoms | 63/472 infants had swallowing dysfunction. 44 had tracheal aspiration (TA) and 19 laryngeal penetrations (LP). GER was found in 79.5% with TA and in 68.4% with LP. |
| Mirić 2014 | Croatia | Observational study | 71 | Children (6-17 yr) | Children with chronic respiratory and other symptoms suggestive for GERD, divided into 2 groups: chronic laryngitis (n=33) and asthma (n=38) | Acid GER was found in 92.1% of patients in the asthmatic group and 90.9% in the chronic laryngitis group. |
| Mittal 2013 | USA | Observational prospective study | 300 | Infants (median: 50 days) | Infants with ALTE | 110 admitted infants had pneumography, of which 29 were positive for GER |
| Miura 2012 | Not applicable | Systematic review | 15 included studies (1513 children) | Children (mean age across the studies: 1-7 yr) | Children with otitis media with effusion (OME) or acute otitis media (AOM) | The mean prevalence of GERD in children with chronic otitis media with effusion was 48.4% (range, 17.6%-64%) and in children with recurrent acute otitis media was 62.9% (range, 61.5%-64.3%). |
| Mohammad 2020 | Egypt | Observational: cross-sectional study | 76 | Infants and children (median 12.6 mo) | Infants and children presented with vomiting (1 month–17 years) | Gastroesophageal reflux was detected in 19/76 (25%) cases. |
| Monti 2017 | Italy | Observational: retrospective study based on administrative databases | 246 | Infants | Infants with ALTE | Of the 148 cases with clinical co-morbidities, 31% had gastroesophageal reflux. |
| Nair 2012 | India | Observational prospective study | 32 | Children (median: 5 yr) | Children undergoing surgery for OME | 11/32 patients had symptoms suggestive of GERD. Of the 32 patients, 21 were found to be positive for Pepsinogen I (PG1) assay. The association between GERD score and pepsinogen level was not significant (P=0.5). |
| Nandan 2021 | India | Observational retrospective study | 104 | Children (mean 4.4 ± 4.2 yr) | Children with recurrent or persistent pneumonia | Gastro-oesophageal reflux disease was found in 7/104 (6.7%) children. |
| Narayanan 2017 | Australia | Observational retrospective study | 1097 | Children (median: 7 mo; range 0.1–24.1 mo) | Children referred for evaluation of reflux disorders | GERD was diagnosed in 57.5% subjects of the total sample. Vomiting (64.4%) and irritability (74%) were the most common symptoms. |
| Owayed 2000 | Canada | Observational study: retrospective medical record review | 238 | Children (mean age 3.7 yr; range, 2.5 mo-15.6 yr) | Children with recurrent pneumonia | Gastroesophageal reflux was found in 13 children (5%). |
| Patria 2013 | Italy | Observational study: case-control | 146 | Children (mean age 7.9 ± 4.5 yr) | Children with recurrent community-acquired pneumonia (rCAP). Symptoms suggestive of GERD were: regurgitation, nausea, chest and/or abdominal pain, hoarseness and dysphagia | 31/146 children had GERD. |
| Pavic 2018 | Croatia | Observational prospective study | 63 | Children (mean age 4.7 yr) | Children with suspected reflux-related otitis media with effusion (OME) | GERD was found in 22 (35%) children based on impedance results and in only nine (14%) children based solely on pH-metry. |
| Pavic 2016 | Croatia | Observational retrospective study | 150 | Children (mean age 7.5 yr; range 0.3-18.0 yr) | Cough of unknown etiology that lasted for at least 2 months | Overall, 125 (83.3%) of children had cough associated with reflux, 84 (56%) with AR, and 122 (81.3%) with WAR. |
| Pavic 2021 | Croatia | Observational prospective study | 21 | Children (mean age, 4.7 mo; range, 0.9–8.9 mo) | Brief resolved unexplained events (BRUE) | BRUE symptoms associated with GER were found in 10 infants (47.6%). Based on the reflux index (RI) (>10%) on pH-metry alone, only 7 (33.3%) infants were diagnosed with GERD. |
| Pavic 2023 | Croatia | Observational prospective study | 66 | Children (mean age 3.9 yr) | Children with recurrent wheezing | GERD was found in 46 (71%) based on MII and only in 8 (12%) based on pH-metry alone. |
| Rollins 1991 | Northern Ireland | Observational prospective study | 100 | Infants (5-90 days) | Consecutive infants who presented with a history of persistent vomiting aged 5 to 90 days | Out of 100 infants, 44 had pyloric stenosis. In the other 56 patients the vomiting settled in six and a barium examination was performed on the remaining 50. This confirmed gastro-oesophageal reflux of varying degrees (eight mild, 30 moderate, and six severe) in 46 of them. |
| Rommel 2003 | Belgium | Observational study | 700 | Children (25 ± 4 mo) | Children referred for assessment of severe feeding difficulty | GERD was the most frequent GI condition (n= 228) and was diagnosed in 33% (228 of 700) of the total group. |
| Rosen 1983 | USA | Observational study | 26 | Infants (2.1 ± 1.3 SD mo) | Infants with unexplained and apparently life-threatening apnea | GER was seen in the radiographs of 19 infants (73%); 18 of these 19 were the same infants who had a history of vomiting. |
| Semmekrot 2010 | The Netherlands | Observational: surveillance study | 110 | Infants (mean age of 11.4 (SD 13.5) weeks) | Infants with apparent life-threatening events (ALTE) | Significant acid reflux could be demonstrated in 31 of 53 infants (58.5%) who underwent pH-metry. while in 22 of 53 infants (41.5%) no acid GER was detected. |
| Sheikh 1999 | USA | Observational study | 84 | Children (8.7 ± 4.6 mo) | Infants with daily wheezing | 54/84 (64%) had positive esophageal pH studies (GER-positive group), and 24 of them (44%) had no gastrointestinal symptoms suggestive of GER. |
| Singendonk 2019 | Not applicable | Systematic review | 25 studies (487,969 children) | Children (0-21 yr) | Children with GERD symptoms: heartburn and/or regurgitation of any severity or compatible symptoms adjusted by a clinician or according to any GERD symptom questionnaire or diary. | "In infants (0–18 months), the overall pooled prevalence of GERD symptoms was 26.9% (95% CI, 20.1–33.7), and ranged from 23.1% to 40.0%.  In children older than 18 months, GERD symptoms show large variation in prevalence between studies (range 0%-38% of study population) and overall, are present in >10% and in 25% on respectively a weekly and monthly basis." |
| Siti Mazliah 2000 | Malaysia | Observational: cross-sectional study | 44 | Children (mean age 9.1 mo, renage 1-58 mo) | Children with persistent respiratory symptoms, such as wheeze, recurrent. aspiration, recurrent chest infection and stridor | 31/44 (70.5%) children were confirmed to have GOR by 24 hour pH esophageal monitoring. |
| Smits 2014 | Not applicable | Systematic review | 6 studies (289 infants) | Infants (0-12 mo) | Infants with apnea | All but one study included prematurely born infants. One study found an increase of apneic events after GER, the remaining 5 studies did not find an association. Two studies assessed apnea followed by GER as well, but did not find sufficient evidence for association. |
| Tieder 2008 | USA | Observational: retrospective study based on administrative databases | 12,067 | Infants (mean age of 0.75 mo, SD 0.31 mo) | Infants with apparent life-threatening event (ALTE) | Among the most common discharge diagnoses there was GER (36.9%) |
| Tolia 2009 | Not applicable | Systematic review | 18 studies | Pediatric population | Children with extra-oesophageal symptoms: respiratory symptoms (asthma, pneumonia, bronchiectasis, ALTE, general respiratory symptoms); Ear, nose and throat symptoms (ENT) symptoms (sinusitis, otitis media, laryngotracheitis); dental symptoms (dental erosion) | The pooled weighted average prevalence of GERD in asthmatic children was 23%, compared with 4% in healthy controls from the same five studies. The majority of studies evaluating the relationship between apparent life-threatening event (ALTE) and GERD did not suggest a causal relationship. Seven studies reported that respiratory symptoms, sinusitis and dental erosion were significantly more prevalent in children with GERD than in controls. |
| Tolia 2003 | USA | Observational retrospective study | 342 | Children (age < 1 yr) | Infants presenting with symptoms suggestive of GERD: regurgitation, choking, irritability, failure to thrive, apparent life-threatening events, or wheezing | GERD was present in 173 children (50.6%) |
| Tutor 2015 | USA | Observational prospective pilot study | 38 | Children (mean age 12.6 ± 5.2 mo) | Children with swallowing dysfunction | GERD was diagnosed in 23/38 children |
| Usta Guc 2014 | Turkey | Observational prospective study | 156 | Children (age 5–16 yr; mean 8.4 ± 2.6) | Children with chronic cough | 5/156 (3.2%) children were diagnosed with gastroesophageal reflux disease |
| Weir 2007 | Australia | Observational: retrospective chart review | 150 | Children (2 weeks-20 yr) | Children with swallowing dysfunction | 68/150 (45.3%) children were diagnosed with GERD |
| Weiss 2010 | Israel | Observational retrospective study | 69 | Children (1 week-1 yr, mean 8 ± 8.3 weeks) | Infants with apparent life-threatening event (ALTE) | The diagnosis was reached in 25 cases. 15/25 (60%) had gastroesophageal reflux. |
| Wild 2011 | USA | Observational: cross-sectional study | 79 | Children (age 9–17 yr) | Children with symptoms of GER (n=59) (abdominal pain, chest pain or heartburn, difficulty swallowing, nausea and/or vomiting, regurgitation, bitter acid taste, burping or belching, choking while swallowing food, upper abdominal pain after eating) and asymptomatic children (controls) (n=20) | Of the 59 pH probe tests performed on symptomatic children, 45 were positive and 14 were negative for GER. Controlling for age, dietary intake, and oral hygiene, there was no association between GER symptoms and dental erosion, by tooth location or affected surface. |
| Yu 2019 | China | Observational prospective study | 118 | Children (mean age, 9.3 ± 1.1 yr) | Childen with chronic cough (duration ranged from 1 to 76 months) | GERD was diagnosed in 7 (5.9%) children |
| Abbreviations: ALTE: Apparent life-threatening event; AOM: Acute otitis media; BRUE: Brief resolved unexplained events; CI: Confidence interval ; CRR: Cough-related-reflux ; ED: Emergency department; EE: Eosinophilic esophagitis ; ENT: Ear, nose and throat symptoms ; GER: Gastroesophageal reflux ; GERD: Gastroesophageal reflux disease ; GI: Gastrointestinal; LP: Laryngeal penetrations ; LPR: Laryngopharyngeal reflux; OME: Otitis media with effusion ; LP: Laryngeal penetrations; PG1: Pepsinogen I; RAOM: Recurrent acute otitis media; rCAP: Recurrent community-acquired pneumonia; RI: Reflux index ; TA: Tracheal aspiration | | | | | | |

### PICO 3 - What are the risk factors for GERD in infants, children, and adolescents?

| Study ID | Country | Study Design | Population | Sample Size | Risk factor | Outcome |
| --- | --- | --- | --- | --- | --- | --- |
| Abdel-Gawa 2009 | Egypt | Prospective cohort study | Children (0-90 months old) in PICU with mechanical ventilation | 24 children (16 cases; 8 controls) | Ventilator associated pneumonia (VAP) | GER in100% cases VS 75% controls |
| Bellomo 2021 | Brazil | Cross-sectional study | Infants (≤1 year old) | 54 infants (25 cases; 29 controls) | Prematurity | GERD in 66.7% cases VS 33.3% controls |
| Bibi 2001 | Israel | NR | Children (3-34 months old) with chronic respiratory symptoms | 116 children (11 laryngomalacia, 13 tracheomalacia, 16 laringotracheomalacia; 41 controls) | Laryngomalacia, tracheomalacia and laryngotracheomalacia | GER in 63% children with laryngomalacia, 53% with tracheomalacia and 87% with laryngotracheomalacia VS 39% controls |
| de Bethman 1993 | France | NR | Infants (≤1 year old) | 217 infants (160 cases; 157 controls) | Apparent life-Threatening event (ALTE) | GOR in 62% cases VS 42% controls |
| Deurloo 2004 | Netherlands | NR | Infants (≤1 year old) | 197 infants (76 cases; 121 controls) | Prematurity | GOR in 42% cases VS 34% controls |
| Durankus 2020 | Turkey | Cross-sectional study | Children (57-76 months old) in NICU | 282 children (137 cases; 145 controls) | Prematurity | GER symptoms in 16.1% cases VS 5.5% controls |
| Elitsur 2009 | USA | Retrospective cohort study | Children (5-14 years old) | 378 children (161 overweight, 184 obese; 393 controls) | Overweight and obesity | GERD in 69% overweight children, 68% obese children VS 65% controls |
| Foroutan 2002 | Iran | NR | Children (4-120 months old) | 62 children (52 cases; 10 controls) | Chronic respiratory symptoms | GERD in 42,3% cases VS NR controls (statistically significance reported) |
| Koebnick 2011 | USA | Cross-sectional study | Children and adolescents (2-19 years old) | 710949 children and adolescents (127279 overweight, 91258 moderately obese, 45394 extremely obese; 246390 controls) | Overweight and obesity | In patients aged 6 – 11 years and 12 – 19 years, GERD was more frequent in moderately and extremely obese than in normal weight patients (P for linear trend 0.001). |
| Kohelet 2004 | Israel | NR | Infants (≤1 year old) | 134 infants (45 cases; 89 controls) | Prematurity | GER in 40% cases VS 49.5% controls |
| Kumar 2012 | India | Prospective case-control study | Neonates (age NR) in NICU | 24 children (14 cases; 10 controls) | Prematurity | GER in 57% cases VS 70% controls |
| Murthy 2018 | USA | Retrospective cohort study | Premature infants (≤1 year old) | 83 premature infants (41 cases; 42 controls) | Naso-gastric tube | No significant difference in acidic events per hour and acid exposure time between the two groups. |
| Pados 2021 | USA | Cross-sectional study | Infants (≤1 year old) | 582 infants (49 born at <32 weeks, 65 born at 32-36 weeks; 468 controls) | Prematurity | Infants born <32 weeks had significantly more symptoms of GER than infants born at 32-36 weeks (P<0.01) and infants born ≥37 weeks (P<0.001) |
| Pashankar 2009 | USA | Prospective cohort study | Children and adolescents (7-16 years old) | 337 children and adolescents (236 obese; 101 controls) | Obesity | GER in 13.1% cases VS 2% controls |
| Pooli 2012 | USA | Cross-sectional study | Children (≤ 2 years old) | 174 children (87 cases; 87 controls) | Vesicouretheral Reflux (VUR) | GER in children aged 0-6 months (40% cases VS 47% controls); GER in children aged 7-12 months (43.6% cases VS 47% controls); GER in children aged 13-18 months (10.9% cases VS 5.8%); GER in children aged 19-24 months (5.4% cases VS 0% controls) |
| Sakaguchi 2014 | Japan | Cross-sectional study | Students (12-15 years old) | 1840 students (260 cases; 1580 controls) | Problem behaviors | GERD in 23.8% cases VS 4.2% controls |
| Størdal 2006 | Norway | NR | Children and adolescents (7-16 years old) | 1136 children (872 cases; 264 controls) | Asthma | GERD in 19.7% cases VS 8.5% controls |
| Thakkar 2010 | USA | Systematic review | Pediatric patients (<18 years old) | Study 1 (Petersen, 1989): 39 patients (24 cases; 15 controls) ; Study 2 (Gustafssen, 1990): 69 patients (42 cases; 27 controls); Study 3 (Chopra, 1995): 90 patients (80 cases; 10 controls); Study 4 (Debley, 2006): 1806 patients (296 cases; 1510 controls); Study 5 (Størdal, 2006): 1136 children (872 cases; 264 controls) | Asthma | Study 1 (Petersen, 1989): GER in 33.3% cases VS 6.7% controls ; Study 2 (Gustafssen, 1990): GER in 50% cases VS 14.8% controls ; Study 3 (Chopra, 1995): GER in 38.8% cases VS 0% controls ; Study 4 (Debley, 2006): GER in 19.3% cases VS 2.5% controls ; Study 5 (Størdal, 2006): GERD in 19.7% cases VS 8.5% controls |
| Abbreviations: ALTE: Apparent life-threatening event; GER: Gastroesophageal reflux; GERD: Gastroesophageal reflux disease; GOR: Gastro-esophageal reflux; NICU: Neonatal Intensive Care Unit; NR: Not reported; VAP: Ventilator associated pneumonia; VUR: Vesicoureteral Reflux. | | | | | | |

### PICO 4 – What is the value of different diagnostic tests for GERD in infants, children, and adolescents?

| Study ID | Country | Study Design | Population (age, characteristics) | Sample Size | Index test | Reference standard | Outcome (diagnostic accuracy or differential diagnosis) |
| --- | --- | --- | --- | --- | --- | --- | --- |
| Abdallah 2017 | Egypt | Prospective cohort study | Infants (7.7±2.2 months old, wheezing) | 38 patients (25 M; 13 F) | MII-pH | Lipid-laden macrophage index (LLMI) | Sensitivity MII-pH: 77%; Specificity MII-pH: 80% |
| Abdollahi 2011 | Iran | Cross-sectional study | Children and adolescents (3-18 years old, symptoms suggestive of GERD) | 263 patients (129 M; 134 F) | Rapid urease test | Endoscopy | Differential diagnosis between GERD and H. pylori infection (31.5% GERD; 22.4% H. Pylori) |
| Aksglæde 2003 | Denmark | NR | Infants (0.6–8.4 months old, symptoms suggestive of GERD) | 21 patients (13 M; 8 F) | Barium swallow (BS) | 24h pH monitoring | Sensitivity BS: 29%; Specificity BS: 50% |
| Al-Khawari 2002 | Kuwait | NR | Children and adolescents (1.3 weeks-17 years old, symptoms suggestive of GERD) | 169 patients (gender NR) | Barium swallow (BS) | 24h pH monitoring | Sensitivity BS: 42%; Specificity BS: 57%; Accuracy BS: 45% |
| Altay 2022 | Turkey | Prospective cross-sectional study | Children and adolescents (2.5 months-17 years old, symptoms suggestive of GERD) | 50 patients (24 M; 26 F): Group 1 (<24 months, n=20), Group 2 (>24 months, n=30) | MII-pH | 24h pH monitoring | Agreement Group 1 pH/MII-pH (+): 20%; Agreement Group 1 pH/MII-pH (-): 35%; Agreement Group 2 pH/MII-pH (+): 13.3%; Agreement Group 2 pH/MII-pH (-): 40% |
| Balson 1998 | USA | NR | Children and adolescents (2-17 years old, asthma) | 79 patients (gender NR) | Gastroesophageal scintigraphy (GES), Barium swallow (BS) | 24h pH monitoring | Sensitivity BS: 46.1%; Specificity BS: 82%; PPV BS: 82%; NPV BS: 51%; Sensitivity GES: 15%; Specificity GES: 72.7%; PPV GES: 50%; NPV GES: 32% |
| Black 1990 | USA | NR | Infants (2 weeks-24 months old, symptoms suggestive of GERD) | 35 patients (22 M; 13 F) | Biopsy | 24h pH monitoring | Sensitivity basal cell thickness: 89%; NPV basal cell thickness: 73%; Sensitivity papillary height: 30%; NPV papillary height: 30%; Sensitivity epithelial eosinophils: 26%; NPV epitelial eosinophils: 29%; Sensitivity lamina propria eosinophils: 41%; NPV lamina propria eosinophils: 33%; Sensitivity epithelial neutrophils: 15%; NPV epithelial neutrophils: 26% |
| Blumhagen 1980 | USA | NR | Children (age NR, symptoms suggestive for GERD) | 65 patients (gender NR) | Radionuclide gastroesophagography (GEG) | Acid reflux test | Sensitivity GEG: 75%; Specificity GEG: 71% |
| Dy 2016 | USA | Prospective cross-sectional study | Children and adolescents (1-19 years old, symptoms suggestive of GERD) | 50 patients (34 M; 16 F) | Salivary pepsin test (SPT) | MII-pH | Sensitivity SPT: 42%; Specificity SPT: 58%; Accuracy SPT: 50% |
| Farina 2008 | Italy | NR | Infants (1 month-2 years old, symptoms suggestive for GERD) | 120 patients (68 M; 52 F) | Contrast enhanced colour-Doppler ultrasound (CDUS) | 24h pH monitoring | Sensitivity CDUS: 98%; Accuracy CDUS: 94% |
| Fortunato 2016 | USA | NR | Children and adolescents (0-18 years old, symptoms suggestive of GERD) | 90 patients (54 M; 36 F) | Pepsin score (ELISA analysis of 8 saliva samples) | MII-pH | Sensitivity Pepsin score ≥1: 100.0%; Specificity Pepsin score ≥1: 18.6%; PPV Pepsin score ≥1: 26.0%;NPV Pepsin score ≥1: 100.0%; Sensitivity Pepsin score ≥3: 80.0%; Specificity Pepsin score ≥3: 45.7%; PPV Pepsin score ≥3: 29.6%;NPV Pepsin score ≥1: 88.9%; Sensitivity Pepsin score ≥4: 70.0%; Specificity Pepsin score ≥4: 52.8%; PPV Pepsin score ≥4: 29.8%;NPV Pepsin score ≥4: 86.0%; Sensitivity Pepsin score ≥5: 50.0%; Specificity Pepsin score ≥5: 61.4%; PPV Pepsin score ≥5: 27.0%;NPV Pepsin score ≥5: 81.1%; |
| Haase 1987 | USA | NR | Children and adolescents (0-18 years old, symptoms suggestive of GERD) | 38 patients (gender NR) | Barium swallow (BS) | 24h pH monitoring | Sensitivity pH monitoring: 87%; Specificity pH monitoring: 93%; Accuracy pH monitoring: 92%; Sensitivity BS: 60%; Specificity BS: 93%; Accuracy BS: 64% |
| Haddad 2019 | USA | Prospective cross-sectional study | Children and adolescents (0-18 years old, symptoms suggestive of GERD) | 52 patients (28 M; 24 F): 17 on PPI, 35 untreated | Salivary pepsin test (SPT) | MII-pH | Sensitivity SPT: 43%; Specificity SPT: 50%; PPV SPT: 24%; NPV SPT: 70% |
| James 1999 | UK | NR | Infants (24-31 weeks old, symptoms suggestive of GERD) | 23 patients (14 M; 9 F) | Acid in the oro-pharyngeal secretions (OPS) | 24h pH monitoring | Sensitivity OPS: 88%; Specificity OPS: 80%; PPV OPS: 94%; NPV OPS: 67% |
| Jang 2000 | South Korea | NR | Children (2 months-10 years old, symptoms suggestive for GERD) | 54 patients (27 M; 27 F) | Color Doppler sonoghraphy | 24h pH monitoring | Agreement : 81.5% |
| Macharia 2012 | UK | Retrospective cohort study | Children and adolescents (0.4-18.2 years old, symptoms suggestive of GERD) | 116 patients (gender NR) | Upper gastro-intestinal series with Barium/Iodine (UGIS) | MII-pH | Sensitivity UGIS: 51%; Specificity UGIS: 58%; PPV UGIS: 75%; NPV UGIS: 32% |
| Matrunola 2003 | Italy | NR | Children (2 months-6 years old, symptoms suggestive of GERD) | 21 patients (gender NR) | Ultrasound (US), upper gastrointestinal series (UGIS) | 24h pH monitoring | Sensitivity US: 80%; Specificity US: 50%; PPV US: 15-18%; NPV US: 71%; Sensitivity UGIS: 65%; Specificity UGIS: 40%; PPV UGIS: 10-15%; NPV UGIS: 22% |
| Naik 1985 | USA | NR | Children and adolescents (4 days-16 years old, symptoms suggestive of GERD) | 51 patients (gender NR) | Ultrasound (US) | Barium swallow (BS) | Agreement US and BS: 78.4% |
| Patwari 2002 | India | NR | Children (1-72 months old, symptoms suggestive for GERD) | 60 patients (47 M; 13 F) | Gastroesophageal Scintigraphy (GES), endoscopy, biopsy | 24h pH monitoring | Sensitivity GES: 65.4%; Specificity GES: 97.1%; PPV GES: 94.4%; NPV GES: 78.6%; Sensitivity endoscopy: 42.3%; Specificity endoscopy: 100%; PPV endoscopy: 100%; NPV endoscopy: 69.4%; Sensitivity biopsy: 84.6%; Specificity biopsy: 97.1%; PPV biopsy: 95.6%; NPV biopsy: 89.2% |
| Pezzati 2007 | Italy | NR | Preterm infants (<34 weeks of gestation) in NICU (age NR, symptoms suggestive for GER) | 31 patients (gender NR) | Ultrasound (US) | 24h pH monitoring | Sensitivity US: 38%; Specificity US: 100%; PPV US: 100%; NPV US: 43% |
| Ramenofsky 1985 | USA | NR | Infants (1 week-6 months old, recurrent pneumonia and apnea) | 50 patients (20 M; 30 F): Group 1 (recurrent pneumonia, n=28), Group 2 (apnea, n=22) | Barium swallow (BS) | 24h pH monitoring | Accuracy BS: 46%; Accuracy pH monitoring: 100% |
| Ravelli 1994 | UK | Retrospective cohort study | Children and adolescents (0.4 -14.4 years old, symptoms suggestive of GERD) | 50 patients (gender NR) | Electric impedence tomography (EIT) | 24h pH monitoring | Sensitivity EIT: 94.6%; Specificity EIT: 76.9%; PPV EIT: 92%; NPV EIT: 83% |
| Riccabona 1992 | Austria | NR | Infants (21-252 days old, symptoms suggestive of GERD) | 30 patients (15 M; 15 F) | Ultrasound (US) | 24h pH monitoring | Sensitivity US: 100%; Specificity US: 87.5% |
| Ristic 2017 | Serbia | NR | Children and adolescents (0.04 -18.0 years old, symptoms suggestive of GERD) | 218 patients (117 M; 101 F) | Endoscopy | MII-pH | Sensitivity pH monitoring: 59.4%; Sensitivity MII: 60.2%; Sensitivity endoscopy: 32.9%; Specificity endoscopy: 89.2% |
| Rosen 2006 | USA | Prospective case-control study | Children (6.2 ± 5.3 years old, gastrointestinal and respiratory symtoms) | 25 patients (14 M; 16 F) | MII | 24h pH monitoring | Sensitivity pH monitoring: 80.6% ± 18.2%; Sensitivity MII: 76.1% ± 13.5% |
| Rosen 2012 | USA | Prospective cross-sectional study | Children ( 67±43 months old, chronic cough or asthma) | 50 patients (gender NR) | Pepsin in bronchoaleveolar lavage (BAL) | Endoscopy, MII-pH | PPV BAL pepsin: 50%; NPV BAL pepsin: 71%; Sensitivity BAL pepsin (VS endoscopy): 67%; Specificity BAL pepsin (VS endoscopy): 59%; Sensitivity BAL pepsin (VS pH monitoring): 45%; Specificity BAL pepsin (VS pH monitoring): 56%; Sensitivity BAL pepsin (VS MII): 71%; Specificity BAL pepsin (VS MII): 60% |
| Safe 2016 | Australia | Retrospective cohort study | Children and adolescents (21 days-17.78 years old, gastrointestinal and respiratory symtoms) | 177 patients (92 M; 85 F) | MII, pH monitoring, salivary pepsin test (SPT), Barium swallow (BS), endoscopy | MII-pH | Sensitivity pH monitoring: 32.3%; NPV pH monitoring: 38.8%; Sensitivity MII: 91.0%; NPV MII: 82.6%; Sensitivity SPT: 47.7%; Specificity SPT: 75.0%; PPV SPT: 87.5%; NPV SPT: 28.1%; Sensitivity BS: 25.3%; Specificity BS: 87.5%; PPV BS: 84.6%; NPV BS: 30.1%; Sensitivity endoscopy: 44.1%; Specificity endoscopy: 60.0%; PPV endoscopy: 71.9%; NPV endoscopy: 31.5% |
| Salvatore 2005 | Italy, Belgium | NR | Infants (0.5-12 months old, symptoms suggestive of GERD) | 44 patients (gender NR) | Biopsy | 24h pH monitoring | Sensitivity biopsy in RI>10%: 47%; Specificity biopsy in RI>10%: 81%; PPV biopsy in RI>10%: 62%; NPV biopsy in RI>10%: 71%; Sensitivity biopsy in RI>5%: 65%; Specificity biopsy in RI>5%: 62%; PPV biopsy in RI>5%: 52%; NPV biopsy in RI>5%: 74% |
| Salvatore 2009 | Italy, Belgium | Prospective cohort study | Children (1.5-186 months old, gastrointestinal and respiratory symtoms) | 45 patients (24 M; 21 F) | Biopsy | 24h pH monitoring | Agreement: 42% |
| Seibert 1983 | USA | NR | Children (5.7 months-9.1 years old, gastrointestinal and respiratory symtoms) | 49 patients (gender NR) | Barium swallow (BS), gastroesophageal scintigraphy (GES) | 24h pH monitoring | Sensitivity GES: 79%; Specificity GES: 93%; Sensitivity BS: 86%; Specificity BS: 21% |
| Sevencan 2019 | Turkey | NR | Children and adolescents (6-18 years old, symptoms suggestive of GERD) | 74 patients (42 M; 32 F) | Mean platelet volume (MPV) | Endoscopy, pH monitoring | Sensitivity MPV: 89.0%; Specificity MPV: 89.0%; PPV MPV: 94.3%; NPV MPV: 79.5% |
| Siti Mazliah 2000 | Malaysia | Prospective cross-sectional study | Children (1-58 months old, chronic respiratory symptoms) | 44 patients (19 M; 25 F) | Barium swallow (BS) | 24h pH monitoring | Sensitivity BS: 37.9%; Specificity BS: 75.0%; PPV BS: 78.5% |
| Staiano 1995 | Italy | NR | Infants (2-13 months old, symproms suggestive of GERD) | 25 patients (gender NR) | Cellobiose/mannitol permeability study (CMPS) | Endoscopy, biopsy, pH monitoring | Differential diagnosis between GERD and cows' milk intolerance (64% GER; 16% cows' milk intolerance; 16% GER and cows' milk intolerance) |
| Uslu Kızılkan 2016 | Turkey | NR | Children and adolescents (2.5-17.3 years old, symptoms suggestive of GERD) | 60 patients (34 M; 26 F) | Gastroesophageal Scintigraphy (GES) | MII-pH | Agreement GES and pH monitoring: 48.3%; Agreement GES and MII: 61.7%; Agreement GES and MII-pH: 73.3%; Agreement MII and pH monitoring: 60% |
| Vandenplas 1992 | Belgium | NR | Children (3 months-7 years old, chronic respiratory symptoms) | 65 patients (gender NR) | Gastroesophageal Scintigraphy (GES) | 24h pH monitoring | Agreement: 18% |
| Vandenplas 2004 | Belgium | NR | Infants (1-6 months old, severely distressed) | 60 patients (gender NR) | 24h pH monitoring | Biopsy | Sensitivity pH monitoring: 38.5%; Specificity pH monitoring: 70.6%; PPV pH monitoring: 50.0%; NPV pH monitoring: 60.0% |
| Wenzl 2002 | UK | NR | Infants (86 ± 57 days old, symproms suggestive of GERD) | 50 patients (31 M; 19 F) | MII | 24h pH monitoring | Sensitivity MII: 54.3%; PPV MII: 60.7% |
| Wynchank 1997 | South Africa | NR | Children (1.2-114.0 months old, symptoms suggestive of GERD) | 110 patients (62 M; 48 F): Group A (US and GES on the same day), Group B (US and GES 30 days apart) | Ultrasound (US) | Gastroesophageal scintigraphy (GES) | Sensitivity US (Group A): 86%; Specificity US (Group A): 63%; Sensitivity US (Group B): 88%; Specificity US (Group B): 55% |
| Yang 2015 | China, USA, Moldova | Retrospective cohort study | Children and adolescents (1 week-16 years old, symptoms suggestive of GERD) | 266 patients (132 M; 134 F) | Radionuclide salivagram | Gastroesophageal scintigraphy (GES) | Detection rate GES: 1.9%; Detection rate salivagram: 22.2%; Agreement: 78.2% |
| Abbreviations: BAL; Pepsin in bronchoaleveolar lavage ; BS; Barium swallow; CDUS; Contrast enhanced colour-Doppler ultrasound ; CMPS; Cellobiose/mannitol permeability study ; EIT; Electric impedence tomography ; ELISA; Enzyme linked immunosorbent assay; GEG; Gastro esophago graphy; GERD; Gastroesophageal reflux disease ; GES; Gastroesophageal scintigraphy ; LLMI; Lipid-laden macrophage index; MPV; Mean platelet volume ; NICU; Neonatal intensive care unit; NPV; Negative predictive value; NR; Not reported; OPS; Acid in the oro-pharyngeal secretions ; PPV; Positive predictive value; RI; Resistive Index; SPT; Salivary pepsin test ; UGIS; Upper gastro-intestinal series with Barium/Iodine ; US; Ultrasound | | | | | | | |
