# Supplementary material for: Guidelines on diagnosis and management of gastroesophageal reflux disease in infants, children and adolescents: a joint consensus from Italian pediatric societies (SIP and SIGENP) -part I. diagnosis
Source: Ital J Pediatr. 2026 Apr 11;52:91. doi: 10.1186/s13052-026-02218-5 (PMC13185181; doi:10.1186/s13052-026-02218-5)
Supplement: Supplementary file 3 — Supplementary Material 3 [file 13052_2026_2218_MOESM3_ESM.docx]

# Additional File 3

## Quality assessment tables

### PICO 2– What are the signs and symptoms indicative of GERD?

#### Case series

| Studies | 1. Were there clear criteria for inclusion in the case series? | 2. Was the condition measured in a standard, reliable way for all participants included in the case series? | 3. Were valid methods used for identification of the condition for all participants included in the case series? | 4. Did the case series have consecutive inclusion of participants? | 5. Did the case series have complete inclusion of participants? | 6. Was there clear reporting of the demographics of the participants in the study? | 7. Was there clear reporting of clinical information of the participants? | 8. Were the outcomes or follow up results of cases clearly reported? | 9. Was there clear reporting of the presenting site(s)/clinic(s) demographic information? | 10. Was statistical analysis appropriate? | Comments | Total | Percentage (%) |
| --- | --- | --- | --- | --- | --- | --- | --- | --- | --- | --- | --- | --- | --- |
| Ashorn 2002 | yes | no | yes | no | yes | no | yes | yes | no | yes | 2) The method of measurement of the condition was not the same for all patients. 4) It is not stated that the patients are consecutive. 6) Only age was reported. 9) Not enough details on the setting | 6/10 | 60 |
| Bellomo-Brandao 2021 | yes | yes | yes | no | no | yes | yes | yes | yes | yes | 4) It is not stated that the patients are consecutive. 5) No initial inclusion of all participants | 8/10 | 80 |
| Borrelli 2011 | yes | yes | yes | yes | yes | yes | yes | yes | no | yes | 9) Not enough details on the setting | 9/10 | 90 |
| Bouchard 1999 | yes | yes | yes | no | yes | yes | yes | yes | yes | no | 4) It is not stated that the patients are consecutive. 10) Statistical analysis is poorly reported. | 8/10 | 80 |
| Chen 1991 | yes | no | yes | yes | no | yes | yes | yes | yes | yes | 2) The method of measurement of the condition was not the same for all patients; 5) only 23 out of the over 110 eligible patients were eventually included in the study due to lack of consent from parents | 8/10 | 80 |
| Chopra 1995 | no | yes | yes | no | no | yes | yes | yes | no | no | 1) inclusion criteria not clearly reported; 4) not clear the time span used for patient recruitment; 5) patient recruitment workflow not reported; 9) the paper does not provide enough information on the study setting; 10) statistical analysis poorly reported | 5/10 | 50 |
| Fishbein 2012 | yes | yes | yes | no | yes | yes | yes | yes | no | yes | 4) It is not stated that the patients are consecutive; 9) study setting is not sufficiently described | 8/10 | 80 |
| Foroutan 2002 | yes | yes | yes | yes | no | yes | yes | yes | yes | yes | 5) the paper does not mention the inclusion of "all" patients with a certain criteria, they start mentioning the inclusion of 52 patients | 9/10 | 90 |
| Greifer 2012 | yes | yes | yes | yes | yes | yes | yes | yes | yes | no | 10) there is no mention of a statistical analysis | 9/10 | 90 |
| Karabel 2014 | yes | no | yes | no | no | yes | yes | yes | yes | yes | 2) patients receive different diagnostic tests and treatments; 4) and 5) there is not mention of a complete and consecutive inclusion of participants | 7/10 | 70 |
| Kiram 2023 | yes | yes | no | no | no | yes | yes | yes | yes | no | 3) methodology not sufficiently described; 4) and 5) they mention convenience sampling 10) statistical analysis i poorly described | 6/10 | 60 |
| Kohelet 2004 | no | yes | yes | yes | yes | yes | yes | yes | yes | yes | 1) inclusion criteria not sufficiently detailed | 9/10 | 90 |
| Koivusalo 2011 | yes | yes | yes | yes | yes | yes | yes | yes | no | yes | 9) study setting is not sufficiently described | 9/10 | 90 |
| Lin 2008 | yes | yes | yes | no | yes | yes | yes | yes | no | yes | 4) It is not stated that the patients are consecutive. 9) the paper does not provide enough information on the study setting. | 8/10 | 80 |
| Lupu 2021 | yes | yes | yes | no | no | yes | yes | yes | yes | yes | 4) and 5) there is not mention of a complete and consecutive inclusion of participants | 8/10 | 80 |
| Lupu 2023 | yes | yes | yes | yes | no | no | yes | yes | yes | yes | 5) there is not mention of a complete inclusion of participants; 6) the demographics of study participants were poorly reported | 8/10 | 80 |
| Mirić 2014 | yes | yes | yes | no | no | yes | yes | yes | no | yes | 4) and 5) It is not stated that the patients are consecutive or complete; 9) study setting is not sufficiently described | 7/10 | 70 |
| Kosec 2020 | yes | yes | yes | yes | no | no | yes | yes | yes | yes | 5) It is not stated that the patients are "all"; 6) demographics are poorly described | 8/10 | 80 |
| Nair 2012 | yes | yes | yes | no | yes | yes | yes | yes | yes | yes | 4) It is not stated that the patients are consecutive. | 9/10 | 90 |
| Nandan 2021 | yes | no | yes | no | yes | yes | yes | yes | yes | no | 2) The method of measurement of the condition was not the same for all patients. 4) It is not stated that the patients are consecutive. 10) Statistical analysis is poorly reported. | 7/10 | 70 |
| Pavic 2018 | yes | yes | yes | no | yes | yes | yes | yes | yes | yes | 4) It is not stated that the patients are consecutive. | 9/10 | 90 |
| Rommel 2003 | yes | yes | yes | yes | no | yes | yes | yes | no | yes | 5) Follow-up data are lacking for 7.9% (55 of 700) of patients. 9) the paper does not provide enough information on the study setting | 8/10 | 80 |
| Rosen 1983 | yes | yes | yes | yes | yes | yes | yes | yes | no | no | 9) the paper does not provide enough information on the study setting. 10) Statistical analysis is poorly reported. | 8/10 | 80 |
| Tolia 2003 | yes | yes | yes | no | yes | yes | yes | yes | yes | yes | 4) It is not stated that the patients are consecutive. | 9/10 | 90 |

#### Cohort studies

| Studies | 1.  Were the two groups similar and recruited from the same population? | 2.  Were the exposures measured similarly to assign people to both exposed and unexposed groups? | 3 . Was the exposure measured in a valid and reliable way? | 4.  Were confounding factors identified? | 5.  Were strategies  to  deal with confounding factors stated? | 6.  Were the groups/ participants free of the outcome at the start of the study (or at the moment of exposure)? | 7.  Were the outcomes measured in a valid and reliable way? | 8.  Was the follow up time reported and sufficient to be long enough for outcomes to occur? | 9.  Was follow up complete, and if not, were the reasons to loss to follow up described and explored? | 10.  Were strategies  to address incomplete follow up utilized? | 11.  Was appropriate statistical analysis used? | Comments | Total | Percentage (%) |
| --- | --- | --- | --- | --- | --- | --- | --- | --- | --- | --- | --- | --- | --- | --- |
| Keles 2004 | yes | yes | yes | no | not applicable | yes | yes | no | not applicable | not applicable | yes | 4) and 5) adjustment for confounders is not mentioned; 8) and 9) and 10) authors do not mention a follow-up despite defining the study as prospective | 6/11 | 55 |
| Campanozzi 2009 | not applicable | not applicable | yes | no | not applicable | yes | yes | yes | no | no | yes | 1) and 2) the study does not inlcude a control group; 4) and 5) the issue of confounders is not discussed; 8) they do not perform an analysis on the initial sample size because they lose patients during the follow-up; 9) and 10) missing follow-up strategies are not described | 5/11 | 45 |
| Ferenchak 1994 | not applicable | not applicable | yes | no | not applicable | yes | yes | no | not applicable | not applicable | no | 1) and 2) the study does not include a control group; 4) and 5) the issue of confounders is not discussed: 9) and 10) missing follow-up strategies are not described; 11) statistical analysis is not described | 3/11 | 27 |
| Kotsis 2009 | yes | yes | yes | yes | yes | no | yes | yes | no | no | yes | 6) patients already had OM and GERD, because the study is mostly focused on evaluating OM-GERD association and OM worsening over time; 9) and 10) incomplete follow-up and strategies to address it are not mentioned nor explored, even if authors mention a range of follow-up time | 8/11 | 73 |
| Mercado-Deane 2001 | yes | yes | yes | no | not applicable | no | yes | no | not applicable | not applicable | no | 4) and 5) the issue of confounders is not discussed; 6) some patients already had symptoms; 8) follow-up time not reported; 9) and 10) missing follow-up strategies are not described; 11) statistical analysis not reported | 4/11 | 36 |
| Mittal 2013 | yes | yes | yes | no | not applicable | yes | yes | yes | yes | not applicable | yes | 4) and 5) the issue of confounders is not discussed | 8/11 | 73 |
| Garza 2011 | yes | yes | yes | yes | yes | no | yes | not applicable | not applicable | not applicable | yes | 6) patients already had GERD symptoms; 8) follow-up time not reported; 9) and 10) missing follow-up strategies are not described | 7/11 | 64 |
| Monti 2017 | yes | yes | yes | yes | yes | yes | yes | not applicable | not applicable | not applicable | yes | 8), 9), 10) Not applicable: it was a retrospective study based on HDR database. | 8/11 | 73 |
| Owayed 2000 | yes | yes | yes | yes | no | yes | yes | not applicable | not applicable | not applicable | yes | 5) no explicit approach is described to address all potential confounding factors through statistical analysis. 8), 9), 10) Not applicable: the study is based on a retrospective analysis of hospital data. | 7/11 | 64 |
| Pavic 2016 | not applicable | not applicable | yes | yes | no | yes | yes | no | not applicable | not applicable | yes | 1) and 2) the study does not inlcude a control group; 5) no explicit approach is described to address all potential confounding factors through statistical analysis. 8), 9), 10): the study does not plan a follow-up after the initial monitoring | 5/11 | 45 |
| Pavic 2021 | not applicable | not applicable | yes | yes | no | yes | yes | yes | yes | not applicable | yes | 1) and 2) the study does not inlcude a control group; 5) no explicit approach is described to address all potential confounding factors through statistical analysis. 10): no lost to follow-up reported. | 7/11 | 64 |
| Pavic 2023 | not applicable | not applicable | yes | yes | yes | yes | yes | yes | yes | not applicable | yes | 1) and 2) the study does not inlcude a control group; 10) no lost to follow-up reported. | 8/11 | 73 |
| Rollins 1991 | not applicable | not applicable | yes | yes | not applicable | yes | yes | no | not applicable | not applicable | yes | 1) and 2) the study does not inlcude a control group; 5) confounders excluded as exclusion criteria; 8), 9), 10) follow-up period not reported. | 5/11 | 45 |
| Sheikh 1999 | not applicable | not applicable | yes | yes | not applicable | yes | yes | yes | yes | not applicable | yes | 1) and 2) the study does not inlcude a control group; 5) confounders excluded as exclusion criteria; 10) no lost to follow-up reported. | 7/11 | 64 |
| Tieder 2008 | not applicable | not applicable | yes | yes | yes | yes | yes | not applicable | not applicable | not applicable | yes | 1) and 2) the study does not inlcude a control group; 5) confounders excluded as exclusion criteria; 8), 9), 10) Not applicable: it was a retrospective study based on HDR database. | 6/11 | 55 |
| Tutor 2015 | not applicable | not applicable | yes | yes | no | yes | yes | yes | no | no | yes | 1) and 2) the study does not inlcude a control group; 5) no strategies to deal with confounding factors reported ; 9) only 17 out of 38 infants returned for the second PFT; 10) strategies to address incomplete follow up not reported. | 6/11 | 55 |
| Usta Guc 2014 | not applicable | not applicable | yes | yes | not applicable | yes | no | yes | not applicable | not applicable | yes | 1) and 2) the study does not inlcude a control group; 5) no strategies to deal with confounding factors reported; 7) GER was evaluated only in 5 patients by scintigraphy; 9), 10) no lost to follow-up reported. | 5/11 | 45 |
| Weir 2007 | not applicable | not applicable | yes | yes | yes | yes | yes | yes | not applicable | not applicable | yes | 1) and 2) the study does not inlcude a control group; 9), 10) Not applicable: it was a retrospective study based on hospital medical records. | 7/11 | 64 |
| Weiss 2010 | yes | yes | yes | no | not applicable | yes | yes | not applicable | not applicable | not applicable | yes | 4) and 5) the issue of confounders is not discussed; 8), 9), 10) Not applicable: it was a retrospective study based on HDR database. | 6/11 | 55 |
| Yu 2019 | not applicable | not applicable | yes | yes | yes | yes | yes | not applicable | not applicable | not applicable | yes | 1) and 2) the study does not inlcude a control group; 8), 9), 10) follow-up period not reported. | 6/11 | 55 |

#### Cross-Sectional Studies

| Studies | 1.  Were the criteria for inclusion in the sample clearly defined? | 2.  Were the study subjects and the setting described in detail? | 3.  Was the exposure measured in a valid and reliable way? | 4.  Were objective, standard criteria used for measurement of the condition? | 5.  Were confounding factors identified? | 6.  Were strategies to deal with confounding factors stated? | 7.  Were the outcomes measured in a valid and reliable way? | 8.  Was appropriate statistical analysis used? | Comments | Total | Percentage (%) |
| --- | --- | --- | --- | --- | --- | --- | --- | --- | --- | --- | --- |
| Dahshan 2002 | yes | no | yes | yes | no | not applicable | yes | yes | 2) setting is poorly described; 5) and 6) there is no mention of a strategy aimed to control for confounders; 6) at the beginning of the study, patients have suspected GERD and are not explicitly free of outcome | 5/8 | 62.5 |
| Gupta 2006 | yes | no | yes | yes | no | not applicable | yes | no | 2) setting is poorly described; 5) and 6) there is no mention of a strategy aimed to control for confounders; 8) statistical analysis is not described | 4/8 | 50.0 |
| Mohammad 2020 | yes | yes | yes | yes | unclear | yes | yes | yes | 5) confounders not clearly stated | 7/8 | 87.5 |
| Narayanan 2017 | yes | yes | yes | yes | no | not applicable | yes | yes | 5) confounders not clearly stated | 6/8 | 75.0 |
| Semmekrot 2010 | yes | yes | yes | yes | no | not applicable | yes | no | 5) confounders not clearly stated; 8) statistical analysis is not described | 5/8 | 62.5 |
| Siti Mazliah 2000 | yes | yes | yes | yes | no | not applicable | yes | yes | 5) confounders not clearly stated | 6/8 | 75.0 |
| Wild 2011 | yes | yes | yes | yes | yes | yes | yes | yes |  | 8/8 | 100.0 |

#### Case-control studies

| Studies | 1. Were the groups comparable other than the presence of disease in cases or the absence of disease in controls? | 2. Were cases and controls matched appropriately? | 3. Were the same criteria used for identification of cases and controls? | 4. Was exposure measured in a standard, valid and reliable way? | 5. Was exposure measured in the same way for cases and controls? | 6. Were confounding factors identified? | 7. Were strategies to deal with confounding factors stated? | 8. Were outcomes assessed in a standard, valid and reliable way for cases and controls? | 9. Was the exposure period of interest long enough to be meaningful? | 10. Was appropriate statistical analysis used? | Comments | Total | Percentage (%) |
| --- | --- | --- | --- | --- | --- | --- | --- | --- | --- | --- | --- | --- | --- |
| Patria 2013 | yes | yes | yes | yes | yes | yes | yes | yes | yes | yes | - | 10/10 | 100 |

#### Systematic reviews

| Studies | 1. Is the review question clearly and explicitly stated? | 2. Were the inclusion criteria appropriate for the review question? | 3. Was the search strategy appropriate? | 4. Were the sources and resources used to search for studies adequate? | 5. Were the criteria for appraising studies appropriate? | 6. Was critical appraisal conducted by two or more reviewers independently? | 7. Were there methods to minimize errors in data extraction? | 8. Were the methods used to combine studies appropriate? | 9. Was the likelihood of publication bias assessed? | 10. Were recommendations for policy and /or practice supported by the reported data? | 11. Were the specific directives for new research appropriate? | Comments | Total | Percentage (%) |
| --- | --- | --- | --- | --- | --- | --- | --- | --- | --- | --- | --- | --- | --- | --- |
| Coughran 2021 | yes | yes | no | yes | yes | yes | yes | yes | yes | yes | yes | 3) the paper does not provide sufficient detail on search strategies used for other databases than PubMed | 10/11 | 91 |
| Lechien 2020 | no | yes | no | yes | no | no | no | yes | yes | yes | yes | 1) research question not reported 3) the paper does not provide sufficient detail on search strategies (only keywords); 5), 6), 7) screening method is poorly described and there is no explicit statement regarding two reviewers | 6/11 | 55 |
| Li 2022 | yes | yes | yes | yes | yes | yes | yes | yes | yes | yes | yes |  | 11/11 | 100 |
| Miura 2012 | no | yes | yes | yes | yes | yes | yes | yes | no | yes | no | 1) research question not reported; 9) publication bias not assessed; 11) suggestion for future research is not sufficiently explored | 8/11 | 73 |
| Singendonk 2019 | no | yes | yes | yes | yes | no | no | yes | no | yes | yes | 1) research question was not reported; 6), and 7): there is no explicit statement regarding if the procedures were performed by two reviewers; 9) publication bias was not assessed | 7/11 | 64 |
| Smits 2014 | no | yes | no | yes | yes | yes | yes | no | no | yes | yes | 1) research question was not reported; 3) the full search strategy was not reported; 8):studies were not pooled because heterogeneous; however, the statistical analysis paragraph explaining the planned strategy for data synthesis is missing; 9) publication bias not assessed | 7/11 | 64 |
| Tolia 2009 | yes | yes | yes | yes | no | no | no | no | no | yes | yes | 5) quality assessment was not performed; 6), and 7): there is no explicit statement regarding if the procedures were performed by two reviewers; 8) methods for data synthesis is not clearly reported; 9) publication bias was not assessed | 6/11 | 55 |

### PICO 3 - What are the risk factors for GERD?

#### Cohort studies

| Studies | 1.  Were the two groups similar and recruited from the same population? | 2.  Were the exposures measured similarly to assign people to both exposed and unexposed groups? | 3 . Was the exposure measured in a valid and reliable way? | 4.  Were confounding factors identified? | 5.  Were strategies  to  deal with confounding factors stated? | 6.  Were the groups/ participants free of the outcome at the start of the study (or at the moment of exposure)? | 7.  Were the outcomes measured in a valid and reliable way? | 8.  Was the follow up time reported and sufficient to be long enough for outcomes to occur? | 9.  Was follow up complete, and if not, were the reasons to loss to follow up described and explored? | 10.  Were strategies  to address incomplete follow up utilized? | 11.  Was appropriate statistical analysis used? | Tot. | Percentage (%) |
| --- | --- | --- | --- | --- | --- | --- | --- | --- | --- | --- | --- | --- | --- |
| Abdel-Gawa, 2009 | Yes | Yes | Yes | Yes | Yes | Yes | Yes | Not Applicalbe (there is no follow-up) | Not Applicalbe (there is no follow-up) | Not Applicalbe (there is no follow-up) | Yes | 8/11 | 73 |
| Elitsur, 2009 | Yes | Yes | Yes | Yes | Yes | Yes | Yes | Not Applicalbe (there is no follow-up) | Not Applicalbe (there is no follow-up) | Not Applicalbe (there is no follow-up) | Yes | 8/11 | 73 |
| Murthy, 2018 | Yes | Yes | Yes | Yes | Yes | Yes | Yes | Not Applicalbe (there is no follow-up | Not Applicalbe (there is no follow-up) | Not Applicalbe (there is no follow-up) | Yes | 8/11 | 73 |
| Pashankar, 2009 | Yes | Yes | No (no mentions) | Yes | Yes | Yes | Yes | Not Applicalbe (there is no follow-up) | Not Applicalbe there is no follow-up) | Not Applicalbe (there is no follow-up) | Yes | 7/11 | 64 |

#### Cross-Sectional Studies

| Studies | 1.  Were the criteria for inclusion in the sample clearly defined? | 2.  Were the study subjects and the setting described in detail? | 3.  Was the exposure measured in a valid and reliable way? | 4.  Were objective, standard criteria used for measurement of the condition? | 5.  Were confounding factors identified? | 6.  Were strategies to deal with confounding factors stated? | 7.  Were the outcomes measured in a valid and reliable way? | 8.  Was appropriate statistical analysis used? | Tot. | Percentage (%) |
| --- | --- | --- | --- | --- | --- | --- | --- | --- | --- | --- |
| Bellomo, 2021 | Yes | Yes | Yes | Yes | Yes | No (no strategy for confounding factors) | Yes | Yes | 7/8 | 88 |
| Bibi, 2001 | Yes | Yes | Yes | Yes | No (no confounding factors were found) | Not Applicable (no strategy for confounding factors) | Yes | Yes | 6/8 | 75 |
| De Bethman, 1993 | Yes | Yes | Yes | Yes | No (no confounding factors were found) | Not Applicable (no strategy for confounding factors) | Yes | No (no statistical studies were found) | 5/8 | 63 |
| Deurloo, 2004 | Yes | Yes | Yes | Yes | Yes | No (no strategy for confounding factors) | Yes | Yes | 7/8 | 88 |
| Durankus, 2020 | Yes | Yes | Yes | Yes | Yes | No (no strategy for confounding factors) | Yes | Yes | 7/8 | 88 |
| Foroutan, 2002 | Yes | Yes | Yes | No (Use of questionnaires) | No (no confounding factors were found) | Not Applicable (no strategy for confounding factors) | No (Use of questionnaires) | Yes | 4/8 | 50 |
| Koebnick, 2011 | Yes | Yes | Yes | Yes | No (no confounding factors were found) | Not Applicable (no strategy for confounding factors) | Yes | Yes | 6/8 | 75 |
| Kohelet, 2004 | Yes | Yes | Yes | Yes | No (no confounding factors were found) | No (no strategy for confounding factors) | Yes | Yes | 6/8 | 75 |
| Pados, 2021 | Yes | Yes | Yes | No (Use of questionnaires) | Yes | Yes | Yes | Yes | 7/8 | 88 |
| Pooli, 2012 | Yes | Yes | Yes | Yes | Yes | Yes | Yes | Yes | 8/8 | 100 |
| Sakaguchi, 2014 | Yes | Yes | Yes | No (Use of questionnaires) | No (no confounding factors were found) | No  (no strategy for confounding factors) | Yes | Yes | 5/8 | 63 |
| Størdal, 2006 | Yes | Yes | Yes | Yes | Yes | Yes | Yes | Yes | 8/8 | 100 |

#### Case-control studies

| Studies | 1.  Were the groups comparable other than the presence of disease in cases or the absence of disease in controls? | 2.  Were cases and controls matched appropriately? | 3.  Were the same criteria used for identification of cases and controls? | 4.  Was exposure measured in a standard, valid and reliable way? | 5.  Was exposure measured in the same way for cases and controls? | 6.  Were confounding factors identified? | 7.  Were strategies to deal with confounding factors stated? | 8.  Were outcomes assessed in a standard, valid and reliable way for cases and controls? | 9.  Was the exposure period of interest long enough to be meaningful? | 10.  Was appropriate statistical analysis used? | Tot. | Percentage (%) |
| --- | --- | --- | --- | --- | --- | --- | --- | --- | --- | --- | --- | --- |
| Kumar, 2011. | Yes | No (larger group of cases than controls) | Yes | Yes | Yes | No (Unidentified confounding factors) | No (No strategies to limit confounding bias were used) | Yes | Yes | Yes | 7/10 | 70 |

#### Systematic reviews

| Studies | 1.  Is the review question clearly and explicitly stated? | 2 Were the inclusion criteria appropriate for the review question? | 3.  Was the search strategy appropriate? | 4.  Were the sources and resources used to search for studies adequate? | 5.  Were the criteria for appraising studies appropriate? | 6.  Was critical appraisal conducted by two or more reviewers independently? | 7.  Were there methods to minimize errors in data extraction? | 8.  Were the methods used to combine studies appropriate? | 9.  Was the likelihood of publication bias assessed? | 10 Were recommendations for policy and/or practice supported by the reported data? | 11.  Were the specific directives for new research appropriate? | Tot. | Percentage (%) |
| --- | --- | --- | --- | --- | --- | --- | --- | --- | --- | --- | --- | --- | --- |
| Thakkar, 2010 | Yes | Yes | Yes | Yes | Yes | Yes | No  (No strategies were used to avoid errors during data extraction) | Yes | No  (no mentions) | Not Applicable (There are no recommendations) | No (vague hints) | 7/11 | 63 |

### PICO 4 – What is the value of different diagnostic testing for GERD in infants and children?

#### Case series

| Studies | 1. Were there clear criteria for inclusion in the case series? | 2. Was the condition measured in a standard, reliable way for all participants included in the case series? | 3. Were valid methods used for identification of the condition for all participants included in the case series? | 4. Did the case series have consecutive inclusion of participants? | 5. Did the case series have complete inclusion of participants? | 6. Was there clear reporting of the demographics of the participants in the study? | 7. Was there clear reporting of clinical information of the participants? | 8. Were the outcomes or follow up results of cases clearly reported? | 9. Was there clear reporting of the presenting site(s)/clinic(s) demographic information? | 10. Was statistical analysis appropriate? | Total | Percentage (%) |
| --- | --- | --- | --- | --- | --- | --- | --- | --- | --- | --- | --- | --- |
| Staiano 1995 | Yes | Yes | Yes | Yes | Yes | Yes | Yes | Yes | Not Applicable (Not relevant to this study design) | Yes | 9/10 | 90 |

#### Cohort studies

| Studies | 1.  Were the two groups similar and recruited from the same population? | 2.  Were the exposures measured similarly to assign people to both exposed and unexposed groups? | 3 . Was the exposure measured in a valid and reliable way? | 4.  Were confounding factors identified? | 5.  Were strategies  to  deal with confounding factors stated? | 6.  Were the groups/ participants free of the outcome at the start of the study (or at the moment of exposure)? | 7.  Were the outcomes measured in a valid and reliable way? | 8.  Was the follow up time reported and sufficient to be long enough for outcomes to occur? | 9.  Was follow up complete, and if not, were the reasons to loss to follow up described and explored? | 10.  Were strategies  to address incomplete follow up utilized? | 11.  Was appropriate statistical analysis used? | Total | Percentage (%) |
| --- | --- | --- | --- | --- | --- | --- | --- | --- | --- | --- | --- | --- | --- |
| Abdallah 2017 | Not Applicable (only one group considered) | Not Applicable (only one group considered) | Yes | Yes | Yes | Yes | Yes | Not Applicable (no follow-up considered) | Not Applicable (no follow-up considered) | Not Applicable (no follow-up considered) | Yes | 6/11 | 55 |
| Macharia 2012 | Not Applicable (only one group considered) | Not Applicable (only one group considered) | Yes | Yes | No (no strategy used) | Yes | Yes | Not Applicable (no follow-up considered) | Not Applicable (no follow-up considered) | Not Applicable (no follow-up considered) | Yes | 5/11 | 45 |
| Ravelli 1994 | Not Applicable (only one group considered) | Not Applicable (only one group considered) | Yes | No (confounding factors not identified) | No (no strategy used) | Yes | Yes | Not Applicable (no follow-up considered) | Not Applicable (no follow-up considered) | Not Applicable (no follow-up considered) | Yes | 4/11 | 36 |
| Safe 2016 | Not Applicable (only one group considered) | Not Applicable (only one group considered) | Yes | No (confounding factors not identified) | No (no strategy used) | Yes | Yes | Not Applicable (no follow-up considered) | Not Applicable (no follow-up considered) | Not Applicable (no follow-up considered) | Yes | 4/11 | 36 |
| Salvatore 2009 | Not Applicable (only one group considered) | Not Applicable (only one group considered) | Yes | No (confounding factors not identified) | No (no strategy used) | Yes | Yes | Not Applicable (no follow-up considered) | Not Applicable (no follow-up considered) | Not Applicable (no follow-up considered) | Yes | 4/11 | 36 |
| Yang 2015 | Not Applicable (only one group considered) | Not Applicable (only one group considered) | Yes | No (confounding factors not identified) | No (no strategy used) | Yes | Yes | Not Applicable (no follow-up considered) | Not Applicable (no follow-up considered) | Not Applicable (no follow-up considered) | Yes | 4/11 | 36 |

#### Cross-Sectional Studies

| Studies | 1.  Were the criteria for inclusion in the sample clearly defined? | 2.  Were the study subjects and the setting described in detail? | 3.  Was the exposure measured in a valid and reliable way? | 4.  Were objective, standard criteria used for measurement of the condition? | 5.  Were confounding factors identified? | 6.  Were strategies to deal with confounding factors stated? | 7.  Were the outcomes measured in a valid and reliable way? | 8.  Was appropriate statistical analysis used? | Total | Percentage (%) |
| --- | --- | --- | --- | --- | --- | --- | --- | --- | --- | --- |
| Abdollahi 2011 | Yes | Yes | Yes | Yes | Yes | Yes | Yes | Yes | 8/8 | 100 |
| Altay 2022 | Yes | Yes | Yes | Yes | Yes | Yes | Yes | Yes | 8/8 | 100 |
| Dy 2016 | Yes | Yes | Yes | Yes | Yes | No  (No strategy for managing confounding factors) | Yes | Yes | 7/8 | 87.5 |
| Haddad 2019 | Yes | Yes | Yes | Yes | No  (No confounding factors identified) | No  (No strategy for managing confounding factors) | Yes | Yes | 6/8 | 75 |
| Rosen 2012 | Yes | Yes | Yes | Yes | Yes | Yes | Yes | Yes | 8/8 | 100 |
| Siti Mazliah 2000 | Yes | Yes | Yes | Yes | No  (No confounding factors identified) | No  (No strategy for managing confounding factors) | Yes | Yes | 6/8 | 75 |

#### Diagnostic accuracy studies

| Studies | 1. Was a consecutive or random sample of patients enrolled? | 2. Was a case control design avoided? | 3. Did the study avoid inappropriate exclusions? | 4. Were the index test results interpreted without knowledge of the results of the reference standard? | 5. If a threshold was used, was it pre-specified? | 6. Is the reference standard likely to correctly classify the target condition? | 7. Were the reference standard results interpreted without knowledge of the results of the index test? | 8. Was there an appropriate interval between index test and reference standard? | 9. Did all patients receive the same reference standard? | 10. Were all patients included in the analysis? | Total | Percentage (%) |
| --- | --- | --- | --- | --- | --- | --- | --- | --- | --- | --- | --- | --- |
| Aksglæde 2003 | Yes | Yes | Yes | Unclear (Not reported) | Yes | Yes | Unclear (Not reported) | Unclear (Not reported) | Yes | Yes | 7/10 | 70 |
| Al-Khawari 2002 | Yes | Yes | Unclear (Not reported) | Unclear (Not reported) | Yes | Yes | Yes | Unclear (Not reported) | Yes | Yes | 7/10 | 70 |
| Balson 1998 | Yes | Yes | No (Exclusions not described) | Unclear (Not reported) | Yes | Yes | Yes | Unclear (Not reported) | Yes | Yes | 7/10 | 70 |
| Black 1990 | Unclear (Not reported) | Yes | No (Exclusions not described) | Yes | Yes | Yes | Yes | Unclear (Not reported) | Yes | Yes | 7/10 | 70 |
| Blumhagen 1980 | Unclear (Not reported) | Yes | Unclear (Not reported) | Unclear (Not reported) | Yes | Yes | Unclear (Not reported) | Unclear (Not reported) | Yes | Yes | 5/10 | 50 |
| Farina 2008 | Unclear (Not reported) | Yes | No (patients with milk intoleranceexluded) | Yes | Yes | Yes | Yes | Yes | Yes | Yes | 8/10 | 80 |
| Fortunato 2016 | Unclear (Not reported) | No (There are cases and controls) | No (Exclusions not described) | Unclear (Not reported) | No (threshold not stated) | Yes | Unclear (Not reported) | Unclear (Not reported) | Yes | Yes | 3/10 | 30 |
| Haase 1987 | No (Non-consecutive patients) | Yes | Unclear (Not reported) | Unclear (Not reported) | Yes | Yes | Unclear (Not reported) | Unclear (Not reported) | Yes | Yes | 5/10 | 50 |
| James 1999 | Yes | No (There are cases and controls) | Yes | Unclear (Not reported) | Yes | Yes | Unclear (Not reported) | Unclear (Not reported) | Yes | No (not all patients included) | 5/10 | 50 |
| Jang 2000 | Unclear (Not reported) | Yes | Unclear (Not reported) | Unclear (Not reported) | Yes | Yes | Unclear (Not reported) | Unclear (Not reported) | Yes | Yes | 5/10 | 50 |
| Matrunola 2003 | Unclear (Not reported) | Yes | Unclear (Not reported) | Unclear (Not reported) | Unclear (Not reported) | Yes | Unclear (Not reported) | Unclear (Not reported) | Yes | Yes | 4/10 | 40 |
| Naik 1985 | Unclear (Not reported) | Yes | Unclear (Not reported) | Yes | Unclear (Not reported) | Yes | Yes | Yes | Yes | Yes | 7/10 | 70 |
| Patwari 2002 | No (Non-consecutive patients) | Yes | Unclear (Not reported) | Unclear (Not reported) | Yes | Yes | Unclear (Not reported) | Unclear (Not reported) | Yes | Yes | 5/10 | 50 |
| Pezzati 2007 | Yes | Yes | Yes | Yes | Yes | Yes | Yes | Yes | Yes | Yes | 10/10 | 100 |
| Ramenofsky 1985 | Yes | Yes | Unclear (Not reported) | Unclear (Not reported) | Yes | Yes | Unclear (Not reported) | Unclear (Not reported) | Yes | Yes | 6/10 | 60 |
| Riccabona 1992 | Yes | Yes | Unclear (Not reported) | Yes | Yes | Yes | Unclear (Not reported) | Yes | Yes | Yes | 8/10 | 80 |
| Ristic 2017 | Yes | Yes | Yes | Unclear (Not reported) | Yes | Yes | Unclear (Not reported) | Unclear (Not reported) | Yes | Yes | 7/10 | 70 |
| Rosen 2006 | Yes | Yes | Yes | Unclear (Not reported) | Yes | Yes | Unclear (Not reported) | Yes | Yes | Yes | 8/10 | 80 |
| Salvatore 2005 | No (Non-consecutive patients) | Yes | Yes | Unclear (Not reported) | Yes | Yes | Unclear (Not reported) | Unclear (Not reported) | Yes | Yes | 6/10 | 60 |
| Seibert 1983 | Yes | Yes | Yes | Unclear (Not reported) | Yes | Yes | Unclear (Not reported) | Yes | Yes | Yes | 8/10 | 80 |
| Sevencan 2019 | Unclear (Not reported) | No (There are cases and controls) | Yes | Unclear (Not reported) | Yes | Yes | Unclear (Not reported) | Unclear (Not reported) | Yes | Yes | 5/10 | 50 |
| Uslu Kızılkan 2016 | Yes | Yes | Yes | Unclear (Not reported) | Yes | Yes | Unclear (Not reported) | Yes | Yes | Yes | 8/10 | 80 |
| Vandenplas 1992 | Unclear (Not reported) | Yes | Yes | Unclear (Not reported) | Yes | Yes | Unclear (Not reported) | Yes | Yes | Yes | 7/10 | 70 |
| Vandenplas 2004 | Yes | Yes | Yes | Yes | Yes | Yes | Yes | Yes | Yes | Yes | 10/10 | 100 |
| Wenzl 2002 | Unclear (Not reported) | Yes | Unclear (Not reported) | No (index and reference were simultaneous) | Yes | Yes | No (index and reference were simultaneous) | Yes | Yes | Yes | 6/10 | 60 |
| Wynchank 1997 | Unclear (Not reported) | Yes | Unclear (Not reported) | Yes | Yes | Yes | Yes | Yes | Yes | Yes | 8/10 | 80 |
