# Supplementary material for: Guidelines on diagnosis and management of gastroesophageal reflux disease in infants, children and adolescents: a joint consensus from Italian pediatric societies (SIP and SIGENP) -part I. diagnosis
Source: Ital J Pediatr. 2026 Apr 11;52:91. doi: 10.1186/s13052-026-02218-5 (PMC13185181; doi:10.1186/s13052-026-02218-5)
Supplement: Supplementary file 4 — Supplementary Material 4 [file 13052_2026_2218_MOESM4_ESM.docx]

# Additional File 4

## GRADE

### PICO 2– What are the signs and symptoms indicative of GERD?

|  | **Study Design** | **Risk of bias (1)** | **Inconsistency (2)** | **Indirectness (3)** | **Imprecision (4)** | **Other  considerations (5)** | **Quality** | **Notes** |
| --- | --- | --- | --- | --- | --- | --- | --- | --- |
| **ALTE** | | | | | | | | |
| Mittal 2013 | Observational prospective study | not serious | not serious | not serious | not serious | none | high |  |
| Monti 2017 | Observational: retrospective study based on administrative databases | not serious | not serious | not serious | not serious | none | high |  |
| Rosen 1983 | Observational study | not serious | not serious | not serious | serious | none | moderate | 4) < 100 patients |
| Semmekrot 2010 | Observational: surveillance study | serious | not serious | not serious | not serious | none | moderate | 1) 50-69% |
| Tieder 2008 | Observational: retrospective study based on administrative databases | serious | not serious | not serious | not serious | none | moderate | 1) 50-69% |
| Weiss 2010 | Observational retrospective study | serious | not serious | not serious | serious | none | low | 1) 50-69%; 4) < 100 patients |
| **Chronic cough** | | | | | | | | |
| Borrelli 2011 | Observational prospective study | not serious | not serious | not serious | serious | none | moderate | 4) < 100 patients |
| Karabel 2014 | Observational study | not serious | not serious | not serious | not serious | none | high |  |
| Kiran 2023 | Observational study | serious | not serious | not serious | serious | none | low | 1) 50-69%; 4) < 100 patients |
| Pavic 2016 | Observational retrospective study | very serious | not serious | not serious | not serious | none | low | 1) < 50% |
| Usta Guc 2014 | Observational prospective study | very serious | not serious | not serious | not serious | none | low | 1) < 50% |
| Yu 2019 | Observational prospective study | serious | not serious | not serious | not serious | none | moderate | 1) 50-69% |
| **Otitis media** | | | | | | | | |
| Keles 2004 | Observational prospective study | serious | not serious | not serious | serious | none | low | 1) 50-69%; 4) < 100 patients |
| Miura 2012 | Systematic review | not serious | not serious | not serious | not serious | none | high |  |
| Nair 2012 | Observational prospective study | not serious | not serious | not serious | serious | none | moderate | 4) < 100 patients |
| Pavic 2018 | Observational prospective study | not serious | not serious | not serious | serious | none | moderate | 4) < 100 patients |
| **Pneumonia** | | | | | | | | |
| Chen 1991 | Observational prospective study | not serious | not serious | not serious | serious | none | moderate | 4) < 100 patients |
| Nandan 2021 | Observational retrospective study | not serious | not serious | not serious | not serious | none | high |  |
| Owayed 2000 | Observational study: retrospective medical record review | serious | not serious | not serious | not serious | none | moderate | 1) 50-69% |
| Patria 2013 | Observational study: case-control | not serious | not serious | not serious | not serious | none | high |  |
| **Dental erosion** | | | | | | | | |
| Dahshan 2002 | Observational prospective study | serious | not serious | not serious | serious | none | low | 1) 50-69%; 4) < 100 patients |
| Lechien 2020 | Systematic review | serious | not serious | not serious | serious | none | low | 1) 50-69%; 4) wide confidence interval |
| Li 2022 | Systematic review and meta-analysis | not serious | serious | not serious | serious | none | low | 2) high heterogeneity among included studies; 4) wide confidence interval |
| **Wheezing** | | | | | | | | |
| Lupu 2021 | Observational retrospective study | not serious | not serious | not serious | serious | none | moderate | 4) < 100 patients |
| Pavic 2023 | Observational prospective study | not serious | not serious | not serious | serious | none | moderate | 4) < 100 patients |
| Sheikh 1999 | Observational study | serious | not serious | not serious | serious | none | low | 1) 50-69%; 4) < 100 patients |
| **Apnea** | | | | | | | | |
| Koivusalo 2011 | Observational retrospective study | not serious | not serious | not serious | serious | none | moderate | 4) < 100 patients |
| Smits 2014 | Systematic review | serious | not serious | not serious | not serious | none | moderate | 1) 50-69% |
| **BRUE** | | | | | | | | |
| Bellomo-Brandao 2021 | Observational: cross-sectional study | not serious | not serious | not serious | serious | none | moderate | 4) < 100 patients |
| Pavic 2021 | Observational prospective study | serious | not serious | not serious | serious | none | low | 1) 50-69%; 4) < 100 patients |
| **Swallowing dysfunction** | | | | | | | | |
| Tutor 2015 | Observational prospective pilot study | serious | not serious | not serious | serious | none | low | 1) 50-69%; 4) < 100 patients |
| Weir 2007 | Observational: retrospective chart review | serious | not serious | not serious | not serious | none | moderate | 1) 50-69% |
| **Vomiting** | | | | | | | | |
| Mohammad 2020 | Observational: cross-sectional study | not serious | not serious | not serious | serious | none | moderate | 4) < 100 patients |
| Rollins 1991 | Observational prospective study | very serious | not serious | not serious | not serious | none | low | 1) < 50% |
| **Bronchial asthma** | | | | | | | | |
| Chopra 1995 | Observational study | serious | not serious | not serious | serious | none | low | 1) 50-69%; 4) < 100 patients |
| **Chest pain** | | | | | | | | |
| Lin 2008 | Observational retrospective study | not serious | not serious | not serious | not serious | none | high |  |
| **Croup** | | | | | | | | |
| Chougran 2020 | Systematic review and meta-analysis | not serious | serious | not serious | not serious | none | moderate | 2) high heterogeneity among included studies |
| **Feeding difficulty** | | | | | | | | |
| Rommel 2003 | Observational study | not serious | not serious | not serious | not serious | none | high |  |
| **Recurrent RTI** | | | | | | | | |
| Lupu 2023 | Observational retrospective study | not serious | not serious | not serious | serious | none | moderate | 4) < 100 patients |
| **Regurgitation** | | | | | | | | |
| Campanozzi 2009 | Observational: prospective survey | very serious | not serious | not serious | not serious | none | low | 1) < 50% |
| **Mixed symptoms** | | | | | | | | |
| Ashorn 2002 | Observational: retrospective chart review | serious | not serious | not serious | serious | none | low | 1) 50-69%; 4) < 100 patients |
| Bouchard 1999 | Observational: retrospective chart review | not serious | not serious | not serious | not serious | none | high |  |
| Ferenchak 1994 | Observational study | very serious | not serious | not serious | serious | none | very low | 1) < 50%; 4) < 100 patients |
| Fishbein 2012 | Observational: retrospective chart review | not serious | not serious | not serious | serious | none | moderate | 4) < 100 patients |
| Foroutan 2002 | Observational retrospective study | not serious | not serious | not serious | serious | none | moderate | 4) < 100 patients |
| Garza 2011 | Observational retrospective study | serious | not serious | not serious | not serious | none | moderate | 1) 50-69% |
| Greifer 2012 | Observational: retrospective chart review | not serious | not serious | not serious | serious | none | moderate | 4) < 100 patients |
| Gupta 2006 | Observational study | serious | not serious | not serious | not serious | none | moderate | 1) 50-69% |
| Kohelet 2004 | Observational retrospective study | not serious | not serious | not serious | not serious | none | high |  |
| Kosec 2020 | Observational retrospective study | not serious | not serious | not serious | serious | none | moderate | 4) < 100 patients |
| Kotsis 2009 | Observational study | not serious | not serious | not serious | not serious | none | high |  |
| Mercado-Deane 2001 | Observational prospective study | very serious | not serious | not serious | not serious | none | low | 1) < 50% |
| Mirić 2014 | Observational study | not serious | not serious | not serious | serious | none | moderate | 4) < 100 patients |
| Narayanan 2017 | Observational retrospective study | not serious | not serious | not serious | not serious | none | high |  |
| Singendonk 2019 | Systematic review | serious | serious | not serious | not serious | none | low | 1) 50-69%; 2) high heterogeneity among included studies |
| Siti Mazliah 2000 | Observational: cross-sectional study | not serious | not serious | not serious | serious | none | moderate | 4) < 100 patients |
| Tolia 2009 | Systematic review | serious | not serious | not serious | not serious | none | moderate | 1) 50-69% |
| Tolia 2003 | Observational retrospective study | not serious | not serious | not serious | not serious | none | high |  |
| Wild 2011 | Observational: cross-sectional study | not serious | not serious | not serious | serious | none | moderate | 4) < 100 patients |

### PICO 3 - What are the risk factors for GERD?

|  | **Study Design** | **Risk of bias (1)** | **Inconsistency (2)** | **Indirectness (3)** | **Imprecision (4)** | **Other considerations (5)** | **Quality** | **Notes** |
| --- | --- | --- | --- | --- | --- | --- | --- | --- |
| **Apparent life-Threatening event (ALTE)** | | | | | | | | |
| de Bethman 1993 | Cross-sectional study | Serious | Not serious | Not serious | Not serious | None | Moderate | 1) 50-69% |
| **Asthma** | | | | | | | | |
| Størdal 2006 | Cross-sectional study | Not serious | Not serious | Not serious | Not serious | None | High |  |
| Thakkar 2010 | Systematic review | Serious | Not serious | Not serious | Serious | None | Low | 1) 50-69%; 4) Some studies <100 patients |
| **Chronic respiratory symptoms** | | | | | | | | |
| Foroutan 2002 | Cross-sectional study | Serious | Not serious | Not serious | Serious | None | Low | 1) 50-69%; 4) <100 patients |
| **Laryngomalacia, tracheomalacia and laryngotracheomalacia** | | | | | | | | |
| Bibi 2001 | Cross-sectional study | Not serious | Not serious | Not serious | Not serious | None | High |  |
| **Naso-gastric tube** | | | | | | | | |
| Murthy 2018 | Retrospective cohort study | Not serious | Not serious | Serious | Serious | None | Low | 3) No GER/GERD detection; 4) <100 patients |
| **Overweight and obesity** | | | | | | | | |
| Elitsur 2009 | Retrospective cohort study | Not serious | Not serious | Not serious | Not serious | None | High |  |
| Koebnick 2011 | Cross-sectional study | Not serious | Not serious | Not serious | Not serious | None | High |  |
| Pashankar 2009 | Prospective cohort study | Serious | Not serious | Not serious | Not serious | None | Moderate | 1) 50-69% |
| **Prematurity** | | | | | | | | |
| Bellomo 2021 | Cross-sectional study | Not serious | Not serious | Not serious | Serious | None | Moderate | 4) <100 patients |
| Deurloo 2004 | Cross-sectional study | Not serious | Not serious | Not serious | Not serious | None | High |  |
| Durankus 2020 | Cross-sectional study | Not serious | Not serious | Not serious | Not serious | None | High |  |
| Kohelet 2004 | Cross-sectional study | Not serious | Not serious | Not serious | Not serious | None | High |  |
| Kumar 2012 | Prospective case-control study | Not serious | Not serious | Not serious | Serious | None | Moderate | 4) <100 patients |
| Pados 2021 | Cross-sectional study | Not serious | Not serious | Serious | Not serious | None | Moderate | 3) No GER/GERD detection |
| **Problem behaviours** | | | | | | | | |
| Sakaguchi 2014 | Cross-sectional study | Serious | Not serious | Not serious | Not serious | None | Moderate | 1) 50-69% |
| **Ventilator associated pneumonia (VAP)** | | | | | | | | |
| Abdel-Gawa 2009 | Prospective cohort study | Not serious | Not serious | Not serious | Serious | None | Moderate | 4) <100 patients |
| **Vesicouretheral Reflux (VUR)** | | | | | | | | |
| Pooli 2012 | Cross-sectional study | Not serious | Not serious | Not serious | Not serious | None | High |  |

### PICO 4 – What is the value of different diagnostic testing for GERD in infants and children?

|  | **Study Design** | **Risk of bias (1)** | **Inconsistency (2)** | **Indirectness (3)** | **Imprecision (4)** | **Other considerations (5)** | **Quality** | **Note** |
| --- | --- | --- | --- | --- | --- | --- | --- | --- |
| **MII-pH VS Lipid-laden macrophage index (LLMI)** | | | | | | | | |
| Abdallah, 2017 | Prospective cohort study | Serious | Not serious | Not serious | Serious | None | Low | 1) 50-69%; 4) <100 patients |
| **Rapid Urease Test VS Endoscopy** | | | | | | | | |
| Abdollahi, 2011 | Cross-sectional study | Not serious | Not serious | Not serious | Serious | None | Moderate | 4) No confidence intervals |
| **MII-pH VS 24h pH monitoring** | | | | | | | | |
| Altay, 2022 | Prospective cross-sectional study | Not serious | Not serious | Not serious | Very serious | None | Low | 4) <100 patients and no confidence intervals |
| **Barium swallow (BS) VS 24h pH monitoring** | | | | | | | | |
| Aksglæde, 2003 | Diagnostic accuracy study | Not serious | Not serious | Not serious | Very serious | None | Low | 4) <100 patients and no confidence intervals |
| Al-Khawari, 2002 | Diagnostic accuracy study | Not serious | Not serious | Not serious | Serious | None | Moderate | 4) No confidence intervals |
| Haase, 1987 | Diagnostic accuracy study | Serious | Not serious | Not serious | Very serious | None | Low | 1) 50-69%; 4) <100 patients and no confidence intervals |
| Ramenofsky, 1985 | Diagnostic accuracy study | Serious | Not serious | Not serious | Very serious | None | Low | 1) 50-69%; 4) <100 patients and no confidence intervals |
| Siti Mazliah, 2000 | Prospective cross-sectional study | Not serious | Not serious | Not serious | Very serious | None | Low | 4) <100 patients and no confidence intervals |
| **Biopsy VS 24h pH monitoring** | | | | | | | | |
| Black, 1990 | Diagnostic accuracy study | Not serious | Not serious | Serious | Very serious | None | Very low | 3) Bioptic findings instead of patients; 4) <100 patients and no confidence intervals |
| Salvatore, 2005 | Diagnostic accuracy study | Serious | Not serious | Serious | Serious | None | Very low | 1) 50-69%; 3) Reflux index instead of patients; 4) No confidence intervals |
| Salvatore, 2009 | Prospective cohort study | Very serious | Not serious | Not serious | Very serious | None | Very low | 1) <50%; 4) <100 patients and no confidence intervals |
| Vandenplas, 2004 | Diagnostic accuracy study | Not serious | Not serious | Not serious | Very serious | None | Low | 4) <100 patients and large confidence intervals |
| **Radionuclide gastroesophagography (GEG) VS Acid reflux test** | | | | | | | | |
| Blumhagen, 1980 | Diagnostic accuracy study | Serious | Not serious | Not serious | Very serious | None | Very low | 1) 50-69%; 4) <100 patients and no confidence intervals |
| **Salivary pepsin test (SPT) VS MII-pH** | | | | | | | | |
| Dy, 2016 | Prospective cross-sectional study | Not serious | Not serious | Not serious | Serious | None | Moderate | 4) <100 patients |
| Haddad, 2019 | Prospective cross-sectional study | Not serious | Not serious | Not serious | Very serious | None | Low | 4) <100 patients and no confidence intervals |
| **Contrast enhanced colour-Doppler ultrasound (CDUS) VS 24h pH monitoring** | | | | | | | | |
| Farina, 2008 | Diagnostic accuracy study | Not serious | Not serious | Not serious | Serious | None | Moderate | 4) No confidence intervals |
| **Pepsin score (ELISA analysis of 8 saliva samples) VS MII-pH** | | | | | | | | |
| Fortunato, 2016 | Diagnostic accuracy study | Very serious | Not serious | Not serious | Very serious | None | Very low | 1) <50%; 4) <100 patients and no confidence intervals |
| **Acid in the oro-pharyngeal secretions (OPS) VS 24h pH monitoring** | | | | | | | | |
| James, 1999 | Diagnostic accuracy study | Serious | Not serious | Not serious | Very serious | None | Very low | 1) 50-69%; 4) <100 patients and no confidence intervals |
| **Color Doppler sonoghraphy VS 24h pH monitoring** | | | | | | | | |
| Jang, 2000 | Diagnostic accuracy study | Serious | Not serious | Not serious | Very serious | None | Very low | 1) 50-69%; 4) <100 patients and no confidence intervals |
| **Upper gastro-intestinal series with Barium/Iodine (UGIS) VS 24h pH monitoring** | | | | | | | | |
| Macharia, 2012 | Retrospective cohort study | Very serious | Not serious | Not serious | Serious | None | Very low | 1) <50%; 4) No confidence intervals |
| **Ultrasound (US) VS Barium swallow (BS)** | | | | | | | | |
| Naik, 1985 | Diagnostic accuracy study | Not serious | Not serious | Not serious | Very serious | None | Low | 4) <100 patients and no confidence intervals |
| **Ultrasound (US) VS 24h pH monitoring** | | | | | | | | |
| Pezzati, 2007 | Diagnostic accuracy study | Not serious | Not serious | Not serious | Very serious | None | Low | 4) <100 patients and no confidence intervals |
| Riccabona, 1992 | Diagnostic accuracy study | Not serious | Not serious | Not serious | Very serious | None | Low | 4) <100 patients and no confidence intervals |
| **Electric impedence tomography (EIT) VS 24h pH monitoring** | | | | | | | | |
| Ravelli, 1994 | Retrospective cohort study | Very serious | Not serious | Not serious | Very serious | None | Very low | 1) 50-69%; 4) <100 patients and no confidence intervals |
| **Endoscopy VS MII-pH** | | | | | | | | |
| Ristic, 2017 | Diagnostic accuracy study | Not serious | Not serious | Not serious | Not serious | None | High |  |
| **MII VS 24h pH monitoring** | | | | | | | | |
| Rosen, 2006 | Prospective case-control study | Not serious | Not serious | Not serious | Serious | None | Moderate | 4) <100 patients |
| Wenzl, 2002 | Diagnostic accuracy study | Serious | Not serious | Not serious | Very serious | None | Very low | 1) 50-69%; 4) <100 patients and no confidence intervals |
| **Gastroesophageal Scintigraphy (GES) VS MII-pH** | | | | | | | | |
| Uslu Kızılkan, 2016 | Diagnostic accuracy study | Not serious | Not serious | Not serious | Very serious | None | Low | 4) <100 patients and no confidence intervals |
| **Gastroesophageal Scintigraphy (GES) VS 24h pH monitoring** | | | | | | | | |
| Vandenplas, 1992 | Diagnostic accuracy study | Not serious | Not serious | Not serious | Very serious | None | Low | 4) <100 patients and no confidence intervals |
| **Ultrasound (US) VS Gastroesophageal scintigraphy (GES)** | | | | | | | | |
| Wynchank, 1997 | Diagnostic accuracy study | Not serious | Not serious | Not serious | Very serious | None | Low | 4) <100 patients and no confidence intervals |
| **Radionuclide salivogram VS Gastroesophageal scintigraphy (GES)** | | | | | | | | |
| Yang, 2015 | Retrospective cohort study | Very serious | Not serious | Not serious | Serious | None | Very low | 1) <50%; 4) No confidence intervals |
| **Pepsin in bronchoaleveolar lavage (BAL) VS Endoscopy and MII-pH** | | | | | | | | |
| Rosen, 2012 | Prospective cross-sectional study | Not serious | Not serious | Not serious | Very serious | None | Low | 4) <100 patients and no confidence intervals |
| **Mean platelet volume (MPV) VS Endoscopy and 24h pH monitoring** | | | | | | | | |
| Sevencan, 2019 | Diagnostic accuracy study | Serious | Not serious | Not serious | Serious | None | Low | 1) 50-69%; 4) <100 patients |
| **Cellobiose/mannitol permeability study (CMPS) VS Endoscopy, biopsy and 24h pH monitoring** | | | | | | | | |
| Staiano, 1995 | Case series | Not serious | Not serious | Not serious | Very serious | None | Low | 4) <100 patients and no confidence intervals |
| **More than 1 diagnostic exam VS 24 pH monitoring** | | | | | | | | |
| Balson, 1998 | Diagnostic accuracy study | Not serious | Not serious | Not serious | Very serious | None | Low | 4) <100 patients and no confidence intervals |
| Matrunola, 2003 | Diagnostic accuracy study | Very serious | Not serious | Not serious | Very serious | None | Low | 1) <50%; 4) <100 patients and no confidence intervals |
| Patwari, 2002 | Diagnostic accuracy study | Serious | Not serious | Not serious | Very serious | None | Low | 1) 50-69%; 4) <100 patients and no confidence intervals |
| Seibert, 1983 | Diagnostic accuracy study | Not serious | Not serious | Not serious | Very serious | None | Low | 4) <100 patients and no confidence intervals |
| **More than 1 diagnostic exam VS 24 MII-pH** | | | | | | | | |
| Safe, 2016 | Retrospective cohort study | Very serious | Not serious | Not serious | Serious | None | Very low | 1) <50%; 4) No confidence intervals |
