# Supplementary material for: Guidelines on diagnosis and management of gastroesophageal reflux disease in infants, children and adolescents: a joint consensus from Italian pediatric societies (SIP and SIGENP) -part I. diagnosis
Source: Ital J Pediatr. 2026 Apr 11;52:91. doi: 10.1186/s13052-026-02218-5 (PMC13185181; doi:10.1186/s13052-026-02218-5)
Supplement: Supplementary file 5 — Supplementary Material 5 [file 13052_2026_2218_MOESM5_ESM.doc]

***Professor Giovanni Corsello,***

***PhD, MD, Full Professor of Pediatrics,***

***Università di Palermo, Palermo, Italy***

**Editor-in-Chief of** **Italian Journal of Pediatrics**

**Oct 17th, 2025**

Dear Prof. *Corsello*,

We would like to submit the original manuscript entitled *“***GUIDELINES ON DIAGNOSIS AND MANAGEMENT OF GASTROESOPHAGEAL REFLUX DISEASE IN INFANTS, CHILDREN AND ADOLESCENTS: A JOINT CONSENSUS FROM ITALIAN PEDIATRIC SOCIETIES (SIP AND SIGENP) -PART I. DIAGNOSIS*”.***

This manuscript examines the diagnostic assessment of pediatric GER/GERD, synthesizing a systematic review and the work of a 16-member multidisciplinary panel that used an 8-question PICO framework and a two-round Delphi process to produce 14 evidence-based recommendations and practice points. The guideline clarifies age-specific symptom interpretation; emphasizes that infant regurgitation is common and usually self-limited or diet-responsive; and specifies when esophageal pH-impedance monitoring and endoscopy are indicated to phenotype GERD and detect esophagitis. It supports a short empirical PPI “treat-to-test” trial in children with heartburn or severe neurological impairment, while cautioning against over-attribution of extra-esophageal symptoms to GERD. The consensus also highlights high-risk groups (e.g., esophageal malformations, severe neurological impairment, cystic fibrosis, selected syndromes) that warrant multidisciplinary evaluation. Overall, the work aims to reduce practice variability and provide clear, actionable guidance for timely, appropriate diagnosis across pediatric care settings.

The joint involvement of the SIP (Società Italiana di Pediatria) and SIGENP (Società Italiana di Gastroenterologia Epatologia e Nutrizione Pediatrica) underscores the comprehensive, interdisciplinary approach adopted in formulating these guidelines.

There are no prior publications or submissions with any overlapping information, including studies and patients. This manuscript is being submitted only to ***“Italian Journal of Pediatrics”***, it will not be submitted elsewhere while under consideration, and should it be published in ***“*Italian Journal of Pediatrics*”***, it will not be published elsewhere, either in similar form or verbatim, without permission of the editors.

Each author has seen and approved the submission of this version of the manuscript and takes full responsibility for the manuscript. The authors declare no conflict of interest regards to this paper

We hope that by publishing this manuscript in ***“*Italian Journal of Pediatrics*”,*** we will be able to reach a wide audience and provide useful clinical information regarding the management of gastroesophageal reflux disease in infants, children and adolescents

Thank you for your consideration.

Sincerely

Caterina Strisciuglio

On behalf of all the authors
